# RhoA regulates translation of the Nogo-A decoy SPARC in white matter-invading glioblastomas

**DOI:** 10.1007/s00401-019-02021-z

**Published:** 2019-05-06

**Authors:** Peter Wirthschaft, Julia Bode, Himanshu Soni, Fabio Dietrich, Thomas Krüwel, Bernd Fischer, Christiane B. Knobbe-Thomsen, Giulia Rossetti, Andreas Hentschel, Norman Mack, Kai Schönig, Michael O. Breckwoldt, André Schmandke, Stefan Pusch, Jan Medenbach, Martin Bendszus, Martin E. Schwab, Andreas von Deimling, Marcel Kool, Christel Herold-Mende, Guido Reifenberger, Robert Ahrends, Björn Tews

**Affiliations:** 1 Schaller Research Group at the University of Heidelberg, German Cancer Research Center (DKFZ), Im Neuenheimer Feld 581, 69120 Heidelberg, Germany; 2 Molecular Mechanisms of Tumor Invasion (V077), DKFZ, Im Neuenheimer Feld 581, 69120 Heidelberg, Germany; 3 Computational Genome Biology, German Cancer Research Center (DKFZ), Im Neuenheimer Feld 581, 69120 Heidelberg, Germany; 4 Department of Neuropathology, Heinrich Heine University Düsseldorf, Moorenstrasse 5, 40225 Düsseldorf, Germany; 5 German Cancer Consortium (DKTK), Partner Site Essen/Düsseldorf, 40225 Düsseldorf, Germany; 6 Computational Biomedicine, Institute for Advanced Simulation IAS-5 and Institute of Neuroscience and Medicine INM-9, Forschungszentrum Jülich, Wilhelm-Johnen-Straße, 52428 Jülich, Germany; 7 Jülich Supercomputing Centre (JSC), Forschungszentrum Jülich, Wilhelm-Johnen-Straße, 52428 Jülich, Germany; 8 Department of Oncology, Hematology and Stem Cell Transplantation, RWTH Aachen University, Pauwelsstraße 30, 52074 Aachen, Germany; 9 Leibniz-Institut für Analytische Wissenschaften-ISAS-e.V, Otto-Hahn-Str. 6b, 44227 Dortmund, Germany; 10 Division of Pediatric Neurooncology, DKFZ, Im Neuenheimer Feld 580, 69120 Heidelberg, Germany; 11 Department of Molecular Biology, Central Institute of Mental Health, Medical Faculty Mannheim/Heidelberg University, J 5, 68159 Mannheim, Germany; 12 Neuroradiology Department, University Hospital Heidelberg, Im Neuenheimer Feld 400, 69120 Heidelberg, Germany; 13 Clinical Cooperation Unit Neuroimmunology and Brain Tumor Immunology, DKFZ, Im Neuenheimer Feld 280, 69120 Heidelberg, Germany; 14 Brain Research Institute, University of Zurich, Winterthurerstrasse 190, 8057 Zurich, Switzerland; 15 Department of Health Sciences and Technology, ETH Zurich, Universitätstrasse 2, 8092 Zurich, Switzerland; 16 Present Address: Max Planck Institute for Demographic Research, Konrad-Zuse-Straße 1, 18057 Rostock, Germany; 17 Department of Neuropathology, University of Heidelberg, Im Neuenheimer Feld 224, 69120 Heidelberg, Germany; 18 Clinical Cooperation Unit Neuropathology, German Cancer Research Center (DKFZ) and DKTK, Im Neuenheimer Feld 224, 69120 Heidelberg, Germany; 19 Biochemistry I-Institute for Biochemistry, Genetics and Microbiology, University of Regensburg, Universitätsstraße 31, 93053 Regensburg, Germany; 20 Division of Experimental Neurosurgery, Department of Neurosurgery, Heidelberg University Hospital, Im Neuenheimer Feld 400, 69120 Heidelberg, Germany; 21 Present Address: Björn Tews, AbbVie Deutschland GmbH & Co.KG, Wiesbaden, Germany

**Keywords:** SPARC, Nogo-A, RhoA, IRE1α, AKT, ENTPD5, Glioblastoma, Invasion, Post-transcriptional regulation, White matter

## Abstract

Glioblastomas strongly invade the brain by infiltrating into the white matter along myelinated nerve fiber tracts even though the myelin protein Nogo-A prevents cell migration by activating inhibitory RhoA signaling. The mechanisms behind this long-known phenomenon remained elusive so far, precluding a targeted therapeutic intervention. This study demonstrates that the prevalent activation of AKT in gliomas increases the ER protein-folding capacity and enables tumor cells to utilize a side effect of RhoA activation: the perturbation of the IRE1α-mediated decay of SPARC mRNA. Once translation is initiated, glioblastoma cells rapidly secrete SPARC to block Nogo-A from inhibiting migration via RhoA. By advanced ultramicroscopy for studying single-cell invasion in whole, undissected mouse brains, we show that gliomas require SPARC for invading into white matter structures. SPARC depletion reduces tumor dissemination that significantly prolongs survival and improves response to cytostatic therapy. Our finding of a novel RhoA-IRE1 axis provides a druggable target for interfering with SPARC production and underscores its therapeutic value.

**Electronic supplementary material:**

The online version of this article (10.1007/s00401-019-02021-z) contains supplementary material, which is available to authorized users.

## Introduction

Glioblastomas are highly invasive brain tumors. The current standard therapeutic protocol for patients includes tumor resection, radiation and chemotherapy with temozolomide (TMZ) [59]. Nevertheless, these high-grade gliomas invariably relapse, and median patient survival is only 15 months [43]. The invasive nature of glioblastomas is a primary reason for the failure of the current standard therapy [6]. Glioblastoma cells invade the healthy brain parenchyma along anatomical guide structures such as myelinated nerve fibers, which constitute the white matter [20]. Butterfly glioblastomas are a typical example of tumor cell infiltration of the contralateral brain hemisphere across the *corpus callosum*. This structure is a bundle of extensively myelinated nerve fibers that connect both hemispheres [6]. Myelin of the central nervous system contains inhibitory membrane proteins such as Nogo-A, oligodendrocyte-myelin glycoprotein (OMgp) and myelin-associated glycoprotein (MAG), of which Nogo-A is the most abundant and is primarily responsible for inhibiting migration [49]. This intrinsically disordered transmembrane protein contains two inhibitory domains that bind to distinct cell surface receptors. The Nogo66 domain binds to the Nogo receptor 1 (NgR1) [19], whereas the Δ20 domain binds to the sphingosine 1-phosphate receptor 2 (S1PR2) [29]. Both receptors are strong activators of Ras homolog A (RhoA) [49]. Active RhoA induces lamellipodia collapse and stress fiber formation which stop cell migration [51]. Despite the presence of Nogo-A, glioblastoma cells efficiently invade white matter [17].

Constitutive activity of the RAC-α serine/threonine-protein kinase (AKT) is a common feature of highly invasive gliomas that can be caused by amplification of receptor tyrosine kinases, activating mutations in phosphoinositide 3-kinase (PI3 K) subunits or functional inactivation of the tumor suppressor protein phosphatase and tensin homolog (PTEN) [11]. In recent prostate and lung cancer studies, the ectonucleoside triphosphate diphosphohydrolase 5 (ENTPD5) was discovered as being transcriptionally upregulated by activated AKT signaling [7, 14]. The upregulation of ENTPD5 was suggested to confer most of the cancer phenotypes associated with AKT hyperactivity, including a metabolic shift towards anaerobic glycolysis [14]. ENTPD5 sustains high UDP-glucose levels in the endoplasmic reticulum (ER) that are required for efficient glycosylation of secreted proteins [26]. Cancer cells constantly modulate their microenvironment by secreting matricellular proteins. The upregulation of these proteins has been reported in various cancers, including breast cancer, bladder cancer, and gliomas, and has been correlated with poor prognosis [60]. Secreted protein acidic and rich in cysteine (SPARC), a matricellular protein, is strongly expressed in highly invasive gliomas [47]. However, the function of SPARC in glioma biology is not fully understood.

Our study introduces a novel RhoA-driven mechanism utilized by glioblastoma cells to invade the healthy brain parenchyma along myelinated nerve fibers: glioblastoma cells interact with the myelin protein Nogo-A via the S1PR2 receptor. This initially activates anti-migratory RhoA signaling but decreases the ribonucleic activity of the inositol requiring enzyme 1α (IRE1α). Reduced IRE1α activity enables glioblastoma cells with activated AKT signaling to secrete SPARC, which prevents an intrinsically disordered region of Nogo-A-Δ20 from further inducing RhoA signaling via S1PR2. Our study introduces ultramicroscopy as a powerful tool for quantitating glioblastoma invasion of white matter at the single-cell level in whole, undissected brains. By analyzing infiltration into the *corpus callosum*, we provide in vivo evidence that glioblastoma cells require SPARC to invade white-matter structures. SPARC depletion significantly prolonged survival in several pre-clinical mouse glioma models.

## Methods

Extended and detailed experimental procedures are provided in the Supplementary Information (Online Resource 2).

### Cell culture

Patient-derived low-passage glioblastoma cells [27] and established glioma cell lines [1] were previously described.

### Myelin extraction

Total myelin protein extracts were prepared from brains of C57BL/6 or C57BL/6-*Rtn4*^*tm1Schw*^ mice [50]. Human tissue samples were provided by the tissue bank of the National Center of Tumor Diseases (NCT, Heidelberg, Germany) according to the regulations of the tissue bank and with the approval of the Ethics Committee of Heidelberg University.

### Real-time cell analysis (RTCA)

Migration through myelin-coated and electronically integrated transwells was monitored using an xCELLigence RTCA DP analyzer (Acea Biosciences, USA).

### Recombinant proteins

His-tagged recombinant proteins were mainly produced in BL21 (Novagen, Germany) or SHuffle (NEB, Germany) bacteria; Nogo-A and Nogo-B were produced in CHO cells (provided by C Rösli, DKFZ, Germany). EGFP-tagged SPARC, ECL2-EGFP and ECL3-EGFP did not contain a His-tag and were produced in HEK293 cells (ATCC, USA).

### Ultramicroscopy

Tissues were dehydrated and optically cleared as previously described [2]. Samples were imaged with an UltraMicroscope II (LaVision BioTec, Germany).

### Lectin affinity chromatography (LAC) and nano-LC–MS/MS

Conditioned medium was concentrated, dialyzed and equilibrated for LAC using concanavalin A-conjugated agarose resin (ConA; Sigma-Aldrich, Germany). Isolated proteins were analyzed by nanoscale liquid chromatography coupled to tandem mass spectrometry (nano-LC–MS/MS) followed by label-free data analysis.

### Microscale thermophoresis

Ligand binding was measured by microscale thermophoresis using a Nanotemper Monolith NT.115 (NanoTemper Technologies, Germany) as described previously [29].

### Animal experiments

Male NOD.Cg-*Prkdc*^*scid*^*Il2rg*t^*m1Wjl*^/SzJ (NSG) mice (Jacksons, USA), 10–12 weeks of age, were used for xenografts with LN308^EGFP−2A−FLuc^. Animal procedures were in accordance with the institutional animal research guidelines after approval by the regional commission of Karlsruhe, Baden-Wuerttemberg, Germany (file number G223/14). Survival was compared using the log-rank test.

### Immunohistochemistry

Primary isocitrate dehydrogenase (IDH)-wildtype glioblastoma tissue samples of 27 patients were retrieved from the archive of the Department of Neuropathology, Heinrich Heine University Düsseldorf, Germany, and investigated as approved by the local ethics committee (study number 5513). Tumors were diagnosed according to the current World Health Organization classification of CNS tumors [34] and had been molecularly characterized before [15, 63].

## Results

Glioblastoma cells respond to Nogo-A by activating S1PR2 and its downstream effector RhoA

**Fig. 1:**
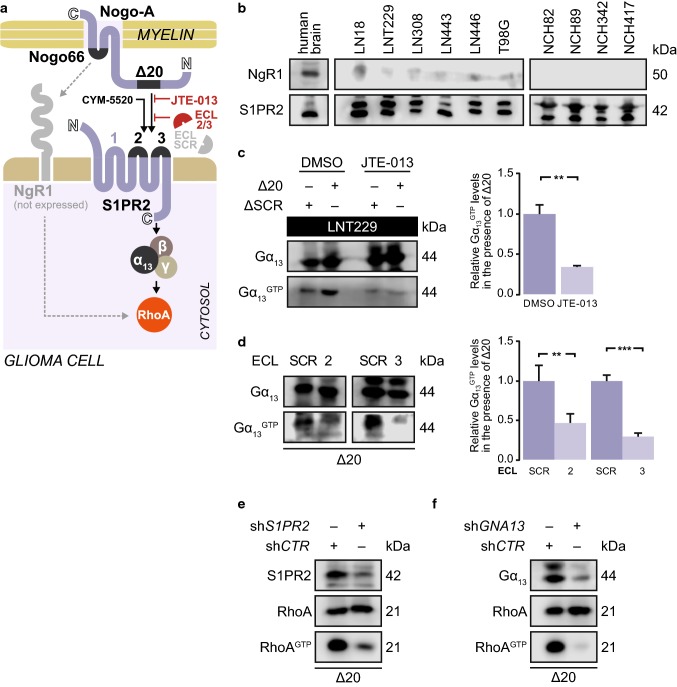
Glioblastoma cells respond to Nogo-A by activating S1PR2 and its downstream effector RhoA. **a** Nogo-A activates S1PR2 via its Δ20 domain. **b** S1PR2 and NgR1 levels in human brain and glioma cell lines (LN18, LNT229, LN308, LN443, LN446, and T98G) and in low passage patient-derived glioblastoma cells (NCH82, NCH89, NCH342, and NCH417).** c**,** d** Gα_13_^GTP^ levels in **c** LNT229 cells treated with 1 µM JTE-013 or ** d** LN308 cells treated with blocking peptides mimicking ECL2 or ECL3 or a scrambled peptide (SCR). Error bars represent the SD, *n* = 3. Unpaired* t*-test, *p ≤ 0.05; **p ≤ 0.01; ***p ≤ 0.001; not significant = *p* > 0.05. **e** S1PR2 activation by Nogo-A induces RhoA. **e**, **f** RhoA^GTP^ levels in LN308 cells. Control shRNA (sh*CTR*); shRNA against *S1PR2* (sh*S1PR2*); shRNA against *GNA13* encoding Gα_13_ (sh*GNA13*).** c**,** d**,** e**,** f** Cells were seeded on Δ20-coated dishes and harvested for activity assays after 30 min (Gα_13_) or 1 h (RhoA). Nogo-A-Δ20 (Δ20); Nogo-A-ΔSCR (ΔSCR)

We have previously demonstrated that Nogo-A activates RhoA by binding via its Δ20 domain (Nogo-A-Δ20) to the Gα_12/13_-coupled receptor S1PR2 [29]. This receptor is expressed in established glioblastoma cell lines and in patient-derived low-passage glioblastoma cells. In contrast, the Nogo-A receptor NgR1 was not expressed by these cells [Fig. 1a, b; Suppl. Figure 1a (Online Resource 1)]. S1PR2 can be activated in glioblastoma cells by the specific receptor agonist CYM-5520, as demonstrated by increased levels of active, GTP-bound Gα_13_ (Gα_13_^GTP^) [Suppl. Figure 1b (Online Resource 1)]. Similarly, Nogo-A-Δ20 activated S1PR2 in glioblastoma cells, and this activation could be attenuated by the receptor antagonist JTE-013. In contrast, a scrambled version of the Δ20 domain, Nogo-A-ΔSCR, did not activate S1PR2 [Fig. 1a, c; Suppl. Figure 1c (Online Resource 1)]. We further confirmed these results in NIH-3T3 fibroblasts, a classical model for studying the inhibitory effects of myelin [3]. NIH-3T3 fibroblasts, which do not express NgR1 [29], activated S1PR2 in the presence of Nogo-A-Δ20, similar to glioblastoma cells [Suppl. Figure 1d (Online Resource 1)]. Moreover, Gα_13_ activation in the presence of Nogo-A-Δ20 was decreased in glioblastoma cells by blocking peptides that mimic extracellular loops (ECL) 2 and 3 of S1PR2 [Fig. 1a, d and Suppl. Figure 1j (Online Resource 1)]. The ECL2/3 peptides physically interact with Nogo-A since EGFP fused to either of these peptides precipitated Nogo-A from myelin extracts [Suppl. Figure 1e (Online Resource 1)]. This interaction required the presence of the Δ20 domain since the Nogo-B isoform, which lacks this domain, did not precipitate the EGFP-ECL2/3 fusion protein [Suppl. Figure 1f (Online Resource 1)].

We have previously shown that Nogo-A-Δ20 repulses neurons and fibroblasts by activating RhoA [29]. Although glioblastoma cells invade myelinated structures [18], they increased the levels of active, GTP-bound RhoA (RhoA^GTP^) when exposed to myelin but only when Nogo-A was present [Fig. 1e; Suppl. Figure 1g (Online Resource 1)]. Furthermore, the Nogo-A-Δ20 domain alone was enough to activate RhoA in glioblastoma cells [Suppl. Figure 1h (Online Resource 1)]; this activation was attenuated by S1PR2 silencing (Fig. 1e). Similar to what occurs in neurons and fibroblasts [29], RhoA activation by Nogo-A-Δ20 was not suppressed by pertussis toxin-mediated inhibition of Gα_i/o_ proteins [Suppl. Figure 1i (Online Resource 1)] but only if Gα_13_-encoding *GNA13* transcripts were silenced (Fig. 1f).

### Glioblastoma cells secrete SPARC upon RhoA activation

**Fig. 2:**
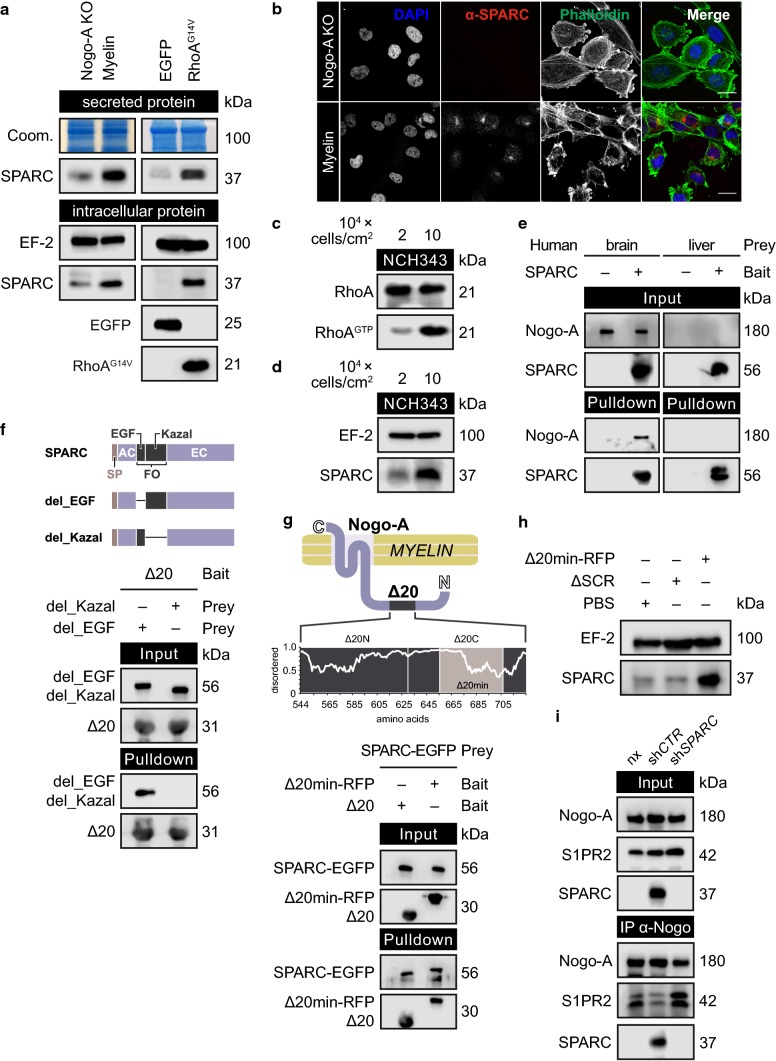
Glioblastoma cells secrete the Nogo-A decoy SPARC upon RhoA activation. **a**, **b**, **h** Cells were grown on protein-coated surfaces for 16 h. **a** SPARC levels in secreted lysates and intracellular protein isolates from LN308 cells. **b** CLSM of LN308 cells. Scale bar: 20 µm. **c**, **d** RhoA^GTP^ or SPARC levels in NCH343 cells grown at either low (2 × 10^4^ cells/cm^2^) or high (10 × 10^4^ cells/cm^2^) density. **e**, **f**, **g** IMAC using **e** TrxA-SPARC and human brain and liver lysates, **f** His-tagged Δ20 and EGFP-tagged SPARC with deletion of either the EGF-like motif (del_EC) or the Kazal-like motif (del_Kazal), or **g** RFP-tagged Δ20 min or Δ20 and SPARC-EGFP. The plot shows the propensity for intrinsic disorder within Nogo-A-Δ20. **h** SPARC levels in the presence of RFP-Δ20 min or a scrambled version. **i** Co-IP of Nogo-A, S1PR2, and SPARC using brain lysates from mice xenografted without glioblastoma cells (nx) or with LN308 glioblastoma cells expressing sh*CTR* or sh*SPARC*. Control shRNA (sh*CTR*); shRNA against *SPARC* (sh*SPARC*). **f**, **g**, **h** Nogo-A(Δ20) (Δ20); RFP-tagged minimal deleted Nogo-A(Δ20) (Δ20 min-RFP); scrambled Nogo-A(Δ20) (ΔSCR)

Since RhoA activation is a key event in inhibitory Nogo-A signaling [49], we expressed constitutively active RhoA (RhoA^G14V^) in glioblastoma cells to identify secreted matricellular proteins that may enable migration. Mass spectrometry data of the RhoA-induced glioma secretome [Suppl. Figure 2a (Online Resource 1), Suppl. Table 1 (Online Resource 3)] were compared with data from a proteome-wide yeast two-hybrid (Y2H) screen, which we had previously conducted to find novel Nogo-A-Δ20 binding partners [29]. We identified SPARC as the only matricellular protein to interact with Nogo-A [Suppl. Figure 2b (Online Resource 1)]. Immunoblotting [Fig. 2a; Suppl. Figure 2c, d (Online Resource 1)] and immunofluorescence staining [Fig. 2b; Suppl. Figure 2e-g (Online Resource 1)] confirmed that glioblastoma cells produced SPARC when exposed to myelin or Nogo-A-Δ20. In these glioblastoma cells, SPARC localized to the ER (co-stained with calnexin; Suppl. Figure 2h) and secretory Golgi vesicles [co-stained with syntaxin-16; Suppl. Figure 2i (Online Resource 1)], indicating a classical secretion pathway. Increased SPARC production in response to Nogo-A was dependent on S1PR2 [Suppl. Figure 2j (Online Resource 1)], which could be stimulated by the receptor agonist CYM-5520 [Suppl. Figure 2k (Online Resource 1)]. While the primary ligand sphingosine 1-phosphate (S1P) was nonessential [Suppl. Figure 2l (Online Resource 1)], an active receptor conformation was required since expression of the conformation-arrested mutant S1PR2^R147C^ [37] prevented SPARC production [Suppl. Figure 2m (Online Resource 1)]. Moreover, SPARC production occurred only when Nogo-A activated S1PR2 in *trans*, whereas overexpression of Nogo-A in glioblastoma cells did not induce SPARC [Suppl. Figure 2n (Online Resource 1)].

SPARC expression has been observed not only in glioblastoma cells in the invasive zone but also in tumor cores with high cell density [45]. In line with this observation and a report of cell density-induced activation of RhoA in mesenchymal stem cells [36], we demonstrate that glioblastoma cells activated RhoA and produced SPARC at high cell density [Fig. 2c, d; Suppl. Figure 2o, p (Online Resource 1)]. In contrast, the non-glioma cell lines A549 and DU145, which have low or absent SPARC expression, did not produce SPARC under conditions of RhoA^G14V^ expression (Suppl. Figure 2q), Nogo-A-Δ20 exposure [Suppl. Figure 2r (Online Resource 1)] or high cell density [Suppl. Figure 2s (Online Resource 1)].

### The Kazal-like module of SPARC interacts with the Δ20 domain of Nogo-A

In validating the Y2H data, we demonstrate that SPARC co-precipitated with Nogo-A in lysate from human brain but not from human liver (Fig. 2e), which lacks Nogo-A expression [57]. Similarly, SPARC co-precipitated with Nogo-A in brain lysates of wild type (WT) but not Nogo-A KO mice [Suppl. Figure 3a (Online Resource 1)]. This interaction depended on the Δ20 domain since Nogo-B, which lacks this domain, did not co-precipitate with SPARC [Suppl. Figure 3b (Online Resource 1)]. Nogo-A-Δ20 alone was enough to precipitate SPARC (Suppl. Figure 3c, d), and the Kazal-like module of the Follistatin domain of SPARC was required for this interaction [Fig. 2f; Suppl. Figure 3e (Online Resource 1)]. Correspondingly, co-precipitation of SPARC with full-length Nogo-A was attenuated when the Kazal-like module was lacking [Suppl. Figure 3f, g (Online Resource 1)].

**Fig. 3:**
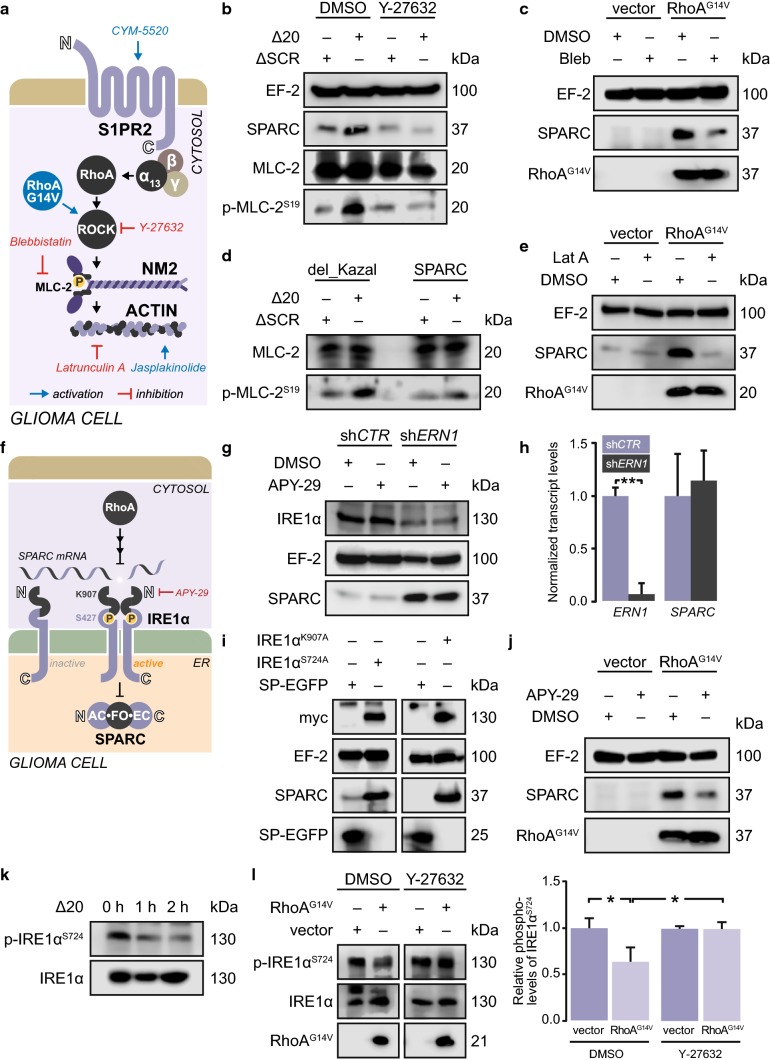
RhoA-induced deactivation of IRE1α initiates SPARC translation. **a** The canonical Rho-ROCK pathway is triggered by Nogo-A-mediated activation of S1PR2. **b** SPARC, MLC-2, and p-MLC-2^S19^ levels in LN308 cells. **c**, **e** SPARC levels in LN443 cells expressing RhoA^G14V^ and treated with either 1 µM blebbistatin (Bleb) or 250 ng/ml latrunculin A (Lat-A) for 16 h. **d** p-MLC-2^S19^ levels in LN308 cells harvested after 1 h. Δ20/ΔSCR was pre-adsorbed with equimolar amounts of either TrxA-SPARC (SPARC) or TrxA-SPARC(del_Kazal) (del_Kazal).** f** Perturbation of IRE1α activity initiates SPARC translation. **g**, **h** SPARC levels in LN308 cells expressing control shRNA (sh*CTR*) or shRNA against *ERN1* (sh*ERN1*), which encodes IRE1α. (h) Gene expression analysis of *ERN1* and *SPARC*. **i** SPARC levels in LN308 cells expressing either IRE1α^S724A^, IRE1α^K907A^ or EGFP fused to an N-terminal signal peptide (SP-EGFP). **j** SPARC levels in LN443 cells expressing RhoA^G14V^ and treated with 1 µM APY-29 for 16 h. **k**, **l** Total- and phospho-IRE1α^S724^ levels in LN308 cells exposed to Δ20 (**k**) or expressing RhoA^G14V^ and treated with 1 µM Y-27632 (**l**). Error bars represent the SD, *n* = 3. Unpaired* t* test, *p ≤ 0.05; **p ≤ 0.01; ***p ≤ 0.001; not significant = *p* > 0.05. **b**, **k** Nogo-A-ΔSCR (ΔSCR); Nogo-A-Δ20 (Δ20)

### A conserved disordered region of Nogo-A-Δ20 is central for its interaction with S1PR2 and SPARC

Nogo-A has been shown to contain intrinsically disordered regions (IDRs) [48] that comprise the Δ20 domain [Suppl. Figure 4a (Online Resource 1)]. IDRs can adapt their structure to interact with different binding partners [10]. Since Nogo-A-Δ20 interacted with both S1PR2 [29] and SPARC [Suppl. Figure 2c (Online Resource 1)], we searched for conserved stretches longer than 20–30 amino acids that could constitute protein–protein recognition motifs within IDRs [52]. Based on the identification of two conserved regions with transient secondary structures, we divided Nogo-A-Δ20 into an N-terminal (Nogo-A-Δ20 N) and a C-terminal (Nogo-A-Δ20C) half [Suppl. Figure 4a (Online Resource 1)]. Only Nogo-A-Δ20C bound to SPARC (Suppl. Figure 4b), which we further narrowed down to a disordered region with transient secondary structures (Nogo-A-Δ20 min) (Fig. 2g). Using ab initio modeling and PROFASI, we found three possible models of Nogo-A-Δ20 min [Suppl. Figure 4c (Online Resource 1)] with similar structural properties and secondary structural propensities [Suppl. Figure 4d, e (Online Resource 1)]. On average, the structural models of Nogo-A-Δ20 min contained 40% α-helices (mainly present in the N-terminal region) and 8% β-sheets (mainly present in the C-terminal region) [Suppl. Figure 4f, g (Online Resource 1)]. Using the 3did database, we found that the interface adopted by binding partners to interact with the Kazal-like module of SPARC contained mostly β-sheets [Suppl. Figure 4h, i, j (Online Resource 1)]. This suggested that β-sheets within Nogo-A-Δ20 min likely represent the binding interface for SPARC. We further demonstrated by affinity chromatography that Nogo-A-Δ20 min is the minimal IDR that is not only required for SPARC binding (Fig. 2g) but also enough to induce SPARC production in glioblastoma cells (Fig. 2h). Moreover, this IDR also interacted with S1PR2: whereas ECL2 bound strongly to Nogo-A-Δ20 N but weakly to Nogo-A-Δ20C [Suppl. Figure 4k (Online Resource 1)], this affinity was reversed for ECL3, which bound more tightly to Nogo-A-Δ20 min than did ECL2 [Suppl. Figure 4l (Online Resource 1)].

**Fig. 4:**
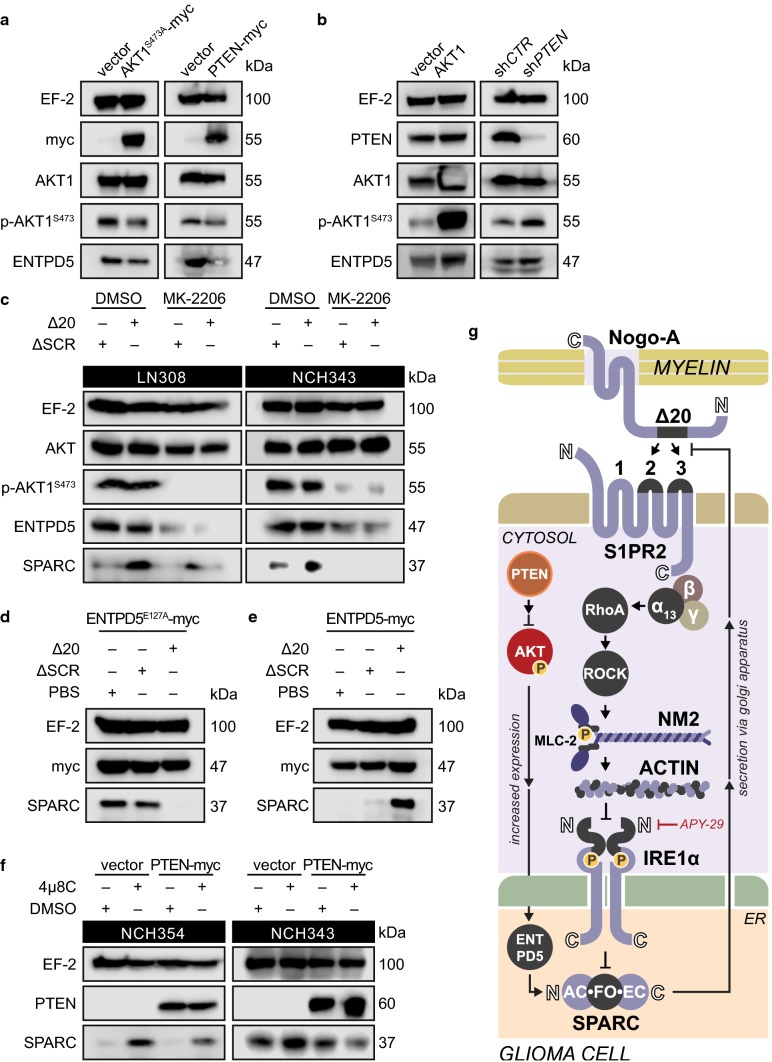
Increased ENTPD5 expression due to high p-AKT levels allows for SPARC production. **a**, **b** ENTPD5 levels in **a** LN308 glioblastoma cells expressing either myc-tagged AKT^S473A^ or PTEN; **b** LNT229 cells expressing either AKT1-myc, control shRNA (sh*CTR*) or shRNA against *PTEN* (sh*PTEN*). **c** ENTPD5 and SPARC levels in glioblastoma cells treated with MK-2206 for 16 h. **d**, **e** SPARC levels in **d** LN308 cells expressing ENTPD5^E127A^-myc or **e** LNT229 cells expressing ENTPD5-myc. **c**, **d**, **e** Cells were exposed to either Nogo-A-Δ20 (Δ20) or Nogo-A-ΔSCR (ΔSCR) for 16 h. **f** SPARC levels in glioblastoma cells expressing PTEN-myc were treated with 1 µM 4µ8C. **g** The RhoA-activated perturbation of IRE1α-regulated mRNA decay (RAPID) pathway

### SPARC is a Nogo-A-decoy that prevents activation of S1PR2

We analyzed whether SPARC competes with S1PR2 for binding to Nogo-A. By microscale thermophoresis, we determined the dissociation constant (K_D_) between SPARC and Nogo-A-Δ20 to be ~ 150 nM, which is within the range of the K_D_ (~ 280 nM) previously determined for the binding of S1PR2 and Nogo-A-Δ20 (Suppl. Figure 4m). We further show that xenografted glioblastoma cells produced SPARC that both co-precipitated with Nogo-A and attenuated the binding of S1PR2 (Fig. 2i), whereas deletion of the Kazal-like module prevented SPARC from co-precipitating with Nogo-A, thus rescuing S1PR2 binding [Suppl. Figure 4n (Online Resource 1)]. Consequently, blocking Nogo-A-Δ20 with recombinant SPARC prevented RhoA activation in glioblastoma cells [Suppl. Figure 4o, p (Online Resource 1)].

### Canonical Rho-ROCK signaling triggers SPARC production

We investigated the molecular mechanism by which glioblastoma cells produce SPARC in response to RhoA activation (Fig. 3a). SPARC production depended on the RhoA effector Rho-associated protein kinase (ROCK) and its downstream target myosin regulatory light chain 2 (MLC-2) because ROCK inhibition with Y-27632 prevented both MLC-2^S19^ phosphorylation and SPARC production in the presence of Nogo-A-Δ20 (Fig. 3b). Accordingly, inhibition of MLC-2 with blebbistatin attenuated RhoA-mediated SPARC production (Fig. 3c). Using the receptor agonist CYM-5520 to activate S1PR2, we confirmed that S1PR2 induced SPARC through the RhoA effector ROCK [Suppl. Figure 5a (Online Resource 1)] and its target MLC-2 [Suppl. Figure 5b (Online Resource 1)]; silencing ROCK or MLC-2 prevented SPARC induction. Similarly, cell density-induced SPARC production was also prevented by inhibiting ROCK or MLC-2 [Suppl. Figure 5c, d (Online Resource 1)]. Moreover, blocking Nogo-A-Δ20 with recombinant SPARC not only prevented RhoA activation [Suppl. Figure 4o, p (Online Resource 1)] but also attenuated the downstream activation of MLC-2 in glioblastoma cells (Fig. 3d). Moreover, we confirmed this RhoA-mediated induction of SPARC in previously reported patient-derived glioblastoma cells which were cultured in the absence of serum [42], thus excluding serum-derived effects [Suppl. Figure 5r (Online Resource 1)].

**Fig. 5:**
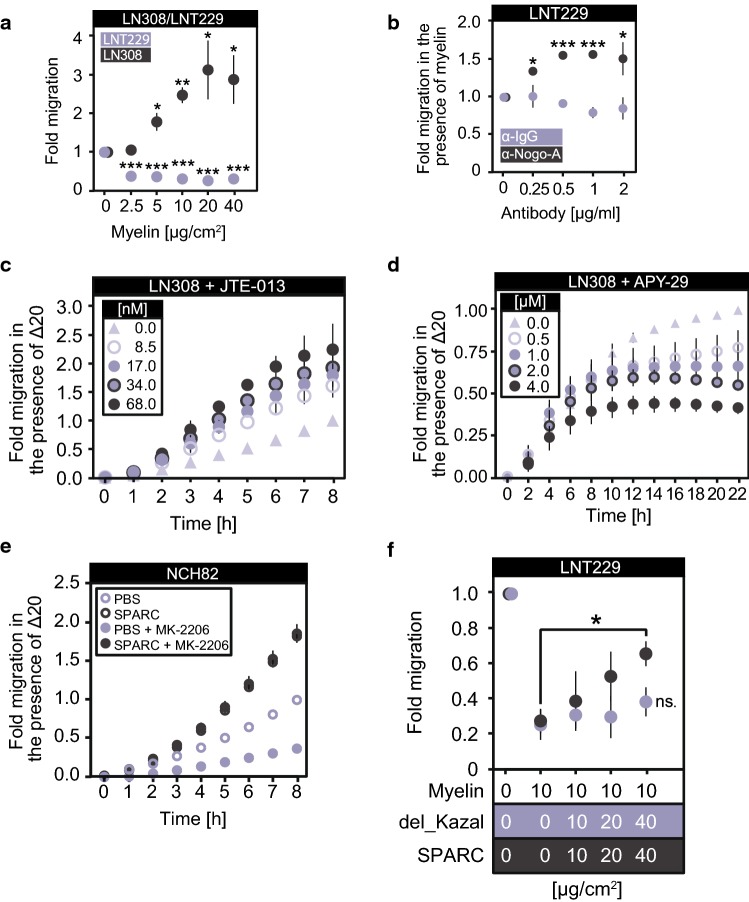
Glioblastoma cells require SPARC to migrate on myelinated structures in vitro. **a**, **b** Migration of **a** LNT229 or LN308 cells in the presence of increasing myelin concentrations or of **b** LNT229 cells in the presence of 20 µg/cm^2^ myelin blocked with increasing concentrations of α-Nogo-A antibody. **c**, **d**, **e** Real-time cell analysis. Transwells were coated with 5 µg/cm^2^ Nogo-A-Δ20 (Δ20). LN308 cell migration in the presence of **c** JTE-013 or **d** APY-29. **e** NCH82 cell migration in the presence of equimolar amounts of SPARC and/or 1 µM MK-2206. **f** LNT229 cell migration in the presence of increasing concentrations of either SPARC or SPARC-del_Kazal (del_Kazal). **a**, **b**, **c**, **d**, **e**, **f** Unpaired *t*-test, error bars represent the SD, *p ≤ 0.05; **p ≤ 0.01; ***p ≤ 0.001; ns. = *p* > 0.05

### RhoA-mediated perturbation of IRE1α-regulated mRNA decay (RAPID) leads to SPARC translation

Rho-ROCK-mediated MLC-2 activation converts non-muscle myosin (NM2) into an assembly-competent form that is required for generating contractile actomyosin bundles called stress fibers [56]. Stress fiber formation in glioblastoma cells in response to RhoA activation induced SPARC which was prevented by interrupting actin assembly with latrunculin A (Lat-A) (Fig. 3e).

Actomyosin contractility was previously linked to the activity of IRE1α [23], a kinase/endoribonuclease that regulates SPARC translation in a mechanism called regulated Ire1-dependent decay of messenger RNA (RIDD). During RIDD, SPARC transcripts are cleaved by IRE1α and further degraded by the RNA exosome complex [25] (Fig. 3f). Glioblastoma cells have been shown to continuously produce high SPARC transcript levels [45]; however, these levels do not correspond with the low SPARC protein level in cells without RhoA activation. Silencing IRE1α or the exosome component 10 (EXOSC10) induced SPARC translation [Fig. 3g; Suppl. Figure 5e (Online Resource 1)], whereas transcript levels remained unchanged [Fig. 3h; Suppl. Figure 5e (Online Resource 1)]. The increase in SPARC translation is also mirrored by an increased polysomal occupancy of SPARC transcripts [Suppl. Figure 5f (Online Resource 1)]. For this posttranscriptional regulation, the RNase activity of IRE1α was required because expression of the RNase-defective, dominant negative mutant IRE1α^K907A^ increased SPARC levels [Fig. 3i; Suppl. Figure 5g (Online Resource 1)]. Consequently, enhancing IRE1α RNase activity with the allosteric activator APY-29 prevented SPARC production when Rho-ROCK signaling was induced by RhoA^G14V^ (Fig. 3J), Nogo-A-Δ20 [Suppl. Figure 5h (Online Resource 1)], or high cell density [Suppl. Figure 5i (Online Resource 1)].

IRE1α phosphorylation has been shown to increase its RNase activity [30]. We demonstrated that S1PR2-induced activation of Rho-ROCK signaling by Nogo-A-Δ20 (Fig. 3k) or CYM-5520 [Suppl. Figure 5j (Online Resource 1)] reduced p-IRE1α^S724^ levels. Similarly, RhoA^G14V^ expression reduced p-IRE1α^S724^ levels, and this was prevented by inhibiting ROCK [Fig. 3l; Suppl. Figure 5q (Online Resource 1)]. We further confirmed that reducing IRE1α phosphorylation increased SPARC production in glioblastoma cells by expressing the phospho-dead mutant IRE1α^S724A^ [Fig. 3i Suppl. Figure 5g (Online Resource 1)]. Moreover, direct induction of stress fiber formation by jasplakinolide also triggered SPARC production which was blunted by enhancing IRE1α activity [Suppl. Figure 5k (Online Resource 1)] and thus, links stress fiber formation to IRE1α activity.

Recently, an IRE1α consensus cleavage site has been identified in a subset of ER-targeted transcripts, often as part of a stem-loop structure [39]. This recognition sequence was also found in the 3′-UTR of *SPARC* and can be cleaved in vitro by recombinant IRE1α if presented as part of a 200 bp oligonucleotide [8]. We probed whether RhoA-induced SPARC translation required the IRE1α recognition site by expressing EGFP-tagged SPARC fused to the 3′-UTR [Suppl. Figure 5m (Online Resource 1)]. SPARC-EGFP (3′-UTR^WT^) was inducible by RhoA activation with Nogo-A-Δ20 similar to endogenous SPARC [Suppl. Figure 5n (Online Resource 1)], whereas EGFP targeted to the ER via an N-terminal signal peptide (SP-EGFP) did not respond [Suppl. Figure 5o (Online Resource 1)]. However, mutated IRE1α recognition sequence (3′-UTR^G1472C^), which disrupted the stem-loop structure, rendered SPARC-EGFP non-inducible and increased the overall SPARC-EGFP levels [Suppl. Figure 5m, p (Online Resource 1)]. This indicates that IRE1α controls SPARC translation endogenously by cleaving the 3′-UTR.

### AKT signaling promotes SPARC production by increasing ENTPD5 expression

Previous studies revealed that phenotypes associated with active, phosphorylated AKT1 (p-AKT1) are mediated through its transcriptional target ENTPD5. This ER-resident enzyme enables a high protein folding capacity of the ER by sustaining UDP-glucose levels [26], which may also be required for the glycosylation and folding of matricellular proteins, such as SPARC. Since AKT signaling is often upregulated in gliomas due to genetic alterations of *PI3* *K* or *PTEN* [4], we investigated whether AKT1-driven ENTPD5 expression is required for glioblastoma cells to produce SPARC.

In glioblastoma cells with high p-AKT1 levels [Fig. 4a; Suppl. Figure 6a (Online Resource 1)], we observed high ENTPD5 expression levels, which were reduced by expressing the phospho-dead mutant AKT1^S473A^. In contrast, glioblastoma cells with low p-AKT1 levels had low ENTPD5 levels, which could be increased by overexpressing AKT1 (Fig. 4b). In PTEN-negative glioblastoma cells, we further show that PTEN re-expression reduced p-AKT1/ENTPD5 levels [Fig. 4a; Suppl. Figure 6a (Online Resource 1)]. However, not all PTEN-positive glioblastoma cells had low p-AKT1/ENTPD5 levels, whereas WT PTEN-expression in LNT229 glioblastoma cells was responsible for their low p-AKT1/ENTPD5 levels (Fig. 4b); LN18 cells retained high p-AKT1/ENTPD5 levels even in the presence of WT PTEN [Suppl. Figure 6b (Online Resource 1)]. This may indicate alternative mechanisms that can circumvent AKT1 suppression, such as gain-of-function mutations of p53 that can modulate WT PTEN activity [33].

We further demonstrate that inhibiting AKT1 phosphorylation with MK-2206 not only reduced ENTPD5 levels but also prevented RhoA-induced SPARC production in glioblastoma cells with high p-AKT1 levels [Fig. 4c; Suppl. Figure 6c (Online Resource 1)]. Likewise, SPARC production was reduced when the enzymatic function of ENTPD5 was disturbed by expressing the dominant negative mutant ENTPD5^E127A^ [Fig. 4d; Suppl. Figure 6d, e (Online Resource 1)] or by re-introducing WT PTEN [Suppl. Figure 6f, g (Online Resource 1)]. Conversely, glioblastoma cells with low AKT1 activity were able to produce SPARC under conditions of RhoA activation when ENTPD5 levels were increased [Fig. 4e; Suppl. Figure 6h, i (Online Resource 1)] or when AKT1 activity was elevated by silencing PTEN [Suppl. Figure 6j (Online Resource 1)]. Moreover, increasing the levels of ENTPD5 compensated for the suppressive effects of PTEN on AKT1 signaling and thus enabled SPARC production [Suppl. Figure 6k (Online Resource 1)]. Similarly, an improved capability to produce SPARC due to increased AKT1 activity after silencing of PTEN was reversed when ENTPD5 was co-silenced [Suppl. Figure 6l (Online Resource 1)]. Finally, we linked IRE1α and AKT1 activity to SPARC production: PTEN-negative glioblastoma cells with high p-AKT1 levels produced SPARC when IRE1α was inactivated with the inhibitor 4µ8C. However, SPARC production due to inactive IRE1α was attenuated when AKT1 activity was reduced by re-expressing PTEN (Fig. 4f). Thus, RhoA-mediated perturbation of IRE1α-regulated mRNA decay (RAPID) induces SPARC production that can only be sustained by high p-AKT1 levels (Fig. 4g).

**Fig. 6:**
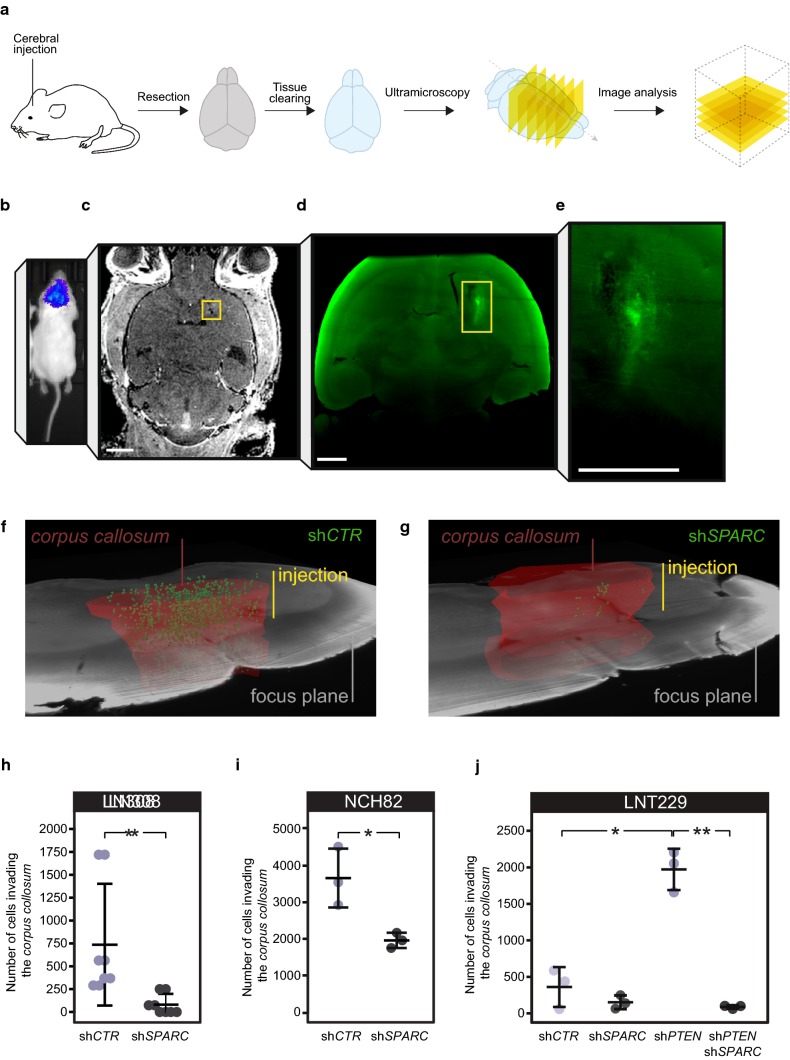
Glioblastoma cells require SPARC to invade white matter in vivo. **a** SPIM workflow for ultramicroscopic analysis of glioma cell invasion. **b**, **c**, **d**, **e** Increased resolution from **b** BLI, **c** MRT with injection site (yellow square); scale bar: 2 mm, **d**, **e** UM; scale bar: 100 μm. **f**, **g** UM analysis of brains xenografted with LN308^EGFP−2A−FLuc^ cells. The focus plane is indicated in gray, and the injection site on the lateral side to the *corpus callosum* (red) is indicated in yellow. **h**, **i**, **j** Quantification of EGFP-labelled **h** LN308 cells, **i** NCH82 cells, and **j** LNT229 cells that invaded the *corpus callosum*. Wilcoxon rank-sum test, error bars represent the SD, *p ≤ 0.05; **p ≤ 0.01; ***p ≤ 0.001; ns. = *p* > 0.05. Control shRNA (sh*CTR*), shRNA against *SPARC* (sh*SPARC*), shRNA against PTEN (sh*PTEN*)

### SPARC secretion enables glioblastoma cells to invade myelinated structures

We showed that glioblastoma cells with high p-AKT1/ENTPD5 levels produced SPARC in response to acute RhoA activation. Therefore, we investigated whether these cells have an advantage in invading myelinated structures. In the presence of myelin or Nogo-A-Δ20, SPARC-producing cells migrated through myelinated transwells [Fig. 5a; Suppl. Figure 7a–c (Online Resource 1)]. In contrast, the invasion of glioblastoma cells with low p-AKT1/ENTPD5/SPARC levels, such as LNT229 cells, was inhibited [Fig. 5a; Suppl. Figure 7a (Online Resource 1)]. These cells invaded through myelinated transwells only when Nogo-A was blocked by an antibody directed against the Δ20 domain (Fig. 5b). Moreover, glioma cell invasion in the presence of Nogo-A-Δ20 was regulated by the activity of S1PR2 and IRE1α. Cell invasion was increased by inhibiting S1PR2 signaling with the antagonist JTE-013 (Fig. 5c) but was diminished when IRE1α was re-activated by APY-29 (Fig. 5d). The superior invasive capability of glioblastoma cells with high p-AKT1/ENTPD5 levels was limited when AKT1 was blocked. However, recombinant SPARC rescued the ability of cells to invade in the presence of Nogo-A-Δ20 [Fig. 5e; Suppl. Figure 7d (Online Resource 1)]. Similarly, glioblastoma cells with low p-AKT1/ENTPD5, and thus low endogenous SPARC production, showed increased invasion through myelinated transwells in the presence of recombinant SPARC but not SPARC (del_Kazal) (Fig. 5f).

To quantify glioma invasion in vivo, we developed an ultramicroscopy (UM)-based approach that allows for the detection of single fluorescence-labelled tumor cells in the undissected mouse brain. To specifically address white-matter invasion, we analyzed cell penetration into the extensively myelinated *corpus callosum* (Fig. 6a). GFP-labelled glioblastoma cells were injected into the basal ganglia and developing tumors were imaged 10 days post implantation. We tracked tumor growth with bioluminescence and magnetic resonance imaging (Fig. 6b, c). UM enabled the precise identification of individual glioblastoma cells (Fig. 6d, e). After 3D analysis of UM-image stacks, we quantified glioblastoma cells (green) that invaded the *corpus callosum* (highlighted in red). These cells either expressed control shRNA (sh*CTR*) (Fig. 6f) or shRNA against *SPARC* (sh*SPARC*) (Fig. 6g). To account for effects related to cell viability and cytotoxicity, we analyzed both cell populations using LDH-, MTT- or RTCA-based experiments which revealed no significant difference in their cell growth [Suppl. Figure 7e, f, g (Online Resource 1)].

We demonstrate that the ability of glioblastoma cells with high p-AKT1/ENTPD5 levels to invade the *corpus callosum* was strongly reduced when SPARC was silenced [Fig. 6h, i; Suppl. Movies 1, 2 (Online Resource 5, 6)]. In comparison, the white-matter invasion of glioblastoma cells with low p-AKT1/ENTPD5 levels, such as LNT229 cells, was restricted but could be improved by silencing PTEN (sh*PTEN*). This effect was reversed by co-silencing SPARC (sh*PTEN*/sh*SPARC*), which confirmed that SPARC was required for glioblastoma cells to invade white matter (Fig. 6j).

### SPARC depletion improves survival in vivo

**Fig. 7:**
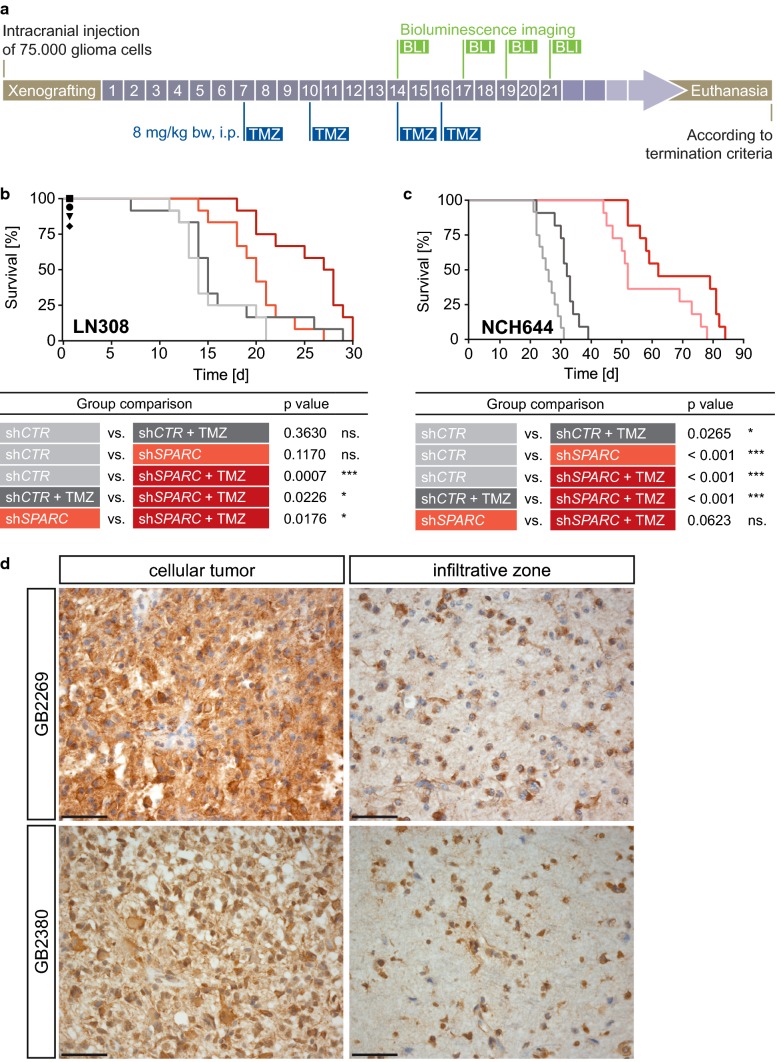
Glioblastoma cells require SPARC for infiltrative growth. **a** Treatment scheme of xenografted NSG mice. **b**, **c** Kaplan–Meier analysis of NSG mice xenografted with LN308 or NCH644 cells expressing EGFP-2A-FLuc and treated with temozolomide (TMZ) or DMSO. Control shRNA (sh*CTR*), shRNA against *SPARC* (sh*SPARC*). Mice that died due to a non-tumor-related cause were censored by a symbol: sh*CTR* (circle), sh*CTR* + TMZ (square), sh*SPARC* (triangle), sh*SPARC* + TMZ (rhombus). ** d** Immunohistochemical staining for SPARC in tissue samples from two representative IDH-wildtype glioblastomas, with (GB2380) or without PTEN mutation (GB2269). Shown are images of cellular tumor areas (left column) and the respective infiltration zone (right column). Brown: specific immunoreactivity. Blue: counterstaining with hemalum. Scale bars: 50 µM

To assess the clinical potential of our molecular findings, we first focused on glioma cell lines with high p-AKT1/ENTPD5 levels and investigated if a reduction in white matter invasion upon SPARC depletion improves survival in a pre-clinical setting (Fig. 7a). We implanted glioblastoma cells that either expressed control shRNA (sh*CTR*) or shRNA against *SPARC* (sh*SPARC*) into NSG mice and confirmed that silencing blunted protein production in vitro (Suppl. Figure 8a) and in vivo [Suppl. Figure 8b (Online Resource 1)]. SPARC depletion in gliomas increased survival, and this increase was further improved when animals were treated with the standard chemotherapeutic temozolomide (TMZ). In contrast, animals bearing gliomas with high SPARC levels showed significantly reduced survival that did not improve upon therapy [Fig. 7b, c; Suppl. Figure 8c, d (Online Resource 1)]. We also xenografted LNT229 cells, which have low p-AKT1/ENTPD5 levels and did not increase SPARC levels in the presence of myelin. Silencing SPARC removed any residual SPARC production from these gliomas [Suppl. Figure 8e (Online Resource 1)], which did not confer an additional survival advantage. However, treatment of LNT229 gliomas with TMZ improved survival [Suppl. Figure 8f, g (Online Resource 1)]. This confirmed the results obtained with gliomas expressing high p-AKT1/ENTPD5 levels, in which SPARC depletion improved the treatment response [Fig. 7b, c; Suppl. Figure 8c, d (Online Resource 1)].

We additionally performed immunohistochemical staining for SPARC in tissue sections of 26 IDH-wildtype glioblastomas [Suppl. Table 2 (Online Resource 1)], which had been previously characterized for glioma-associated genetic alterations [15, 63]. In these studies, we observed high SPARC levels across all tumors, including PTEN-wildtype glioblastomas, both in cellular tumor areas and in less cellular areas of the infiltration zone (Fig. 7d). We confirmed SPARC expression in immunohistochemical stainings of consecutive sections in pAKT-, p53- or EGFRvIII-positive glioblastoma cells in situ [Suppl. Figure 8 h, I, k (Online Resource 1)]. In addition, we performed double-labeling immunofluorescence stainings for SPARC and p53 or SPARC and EGFRvIII on selected tumors which also revealed SPARC expression by glioblastoma cells being positive for either of these tumor cell-specific markers [Suppl. Figure 8 j, l (Online Resource 1)]. These findings are in line with our in vitro data: glioblastomas are able to increase SPARC production even in presence of WT PTEN. Moreover, similar to our observation in high-density cell cultures, glioblastomas also demonstrated stronger SPARC expression in the cellular tumor core compared to the invasive zone [Fig. 7d, Suppl. Figure 8h (Online Resource 1)].

## Discussion

### Nogo-A activates S1PR2 and induces inhibitory Rho-ROCK signaling in glioblastoma cells

Previous studies established NgR1 and S1PR2 as receptors for the inhibitory myelin protein Nogo-A [49]. Since glioblastoma cells are known to invade white matter [6], infiltrating cells could potentially silence these receptors to prevent the induction of inhibitory Nogo-A signaling. Although this was true for NgR1, we found that S1PR2 was still expressed in all investigated cell lines. Our finding for NgR1 in invasive glioma cell lines is in agreement with previous data from human glioma tissues showing a reduction in expression with increasing malignancy [61]. The expression of S1PR2 by glioblastoma cells may be explained by their requirement to balance the input from other expressed S1P receptor subtypes, such as S1PR1 or S1PR3 [44]. Lysophospholipid receptors such as S1PR2 are activated by small molecules [38]. We recently showed binding of the large membrane protein Nogo-A to S1PR2 [29]. Our current study demonstrates that the Δ20 domain of Nogo-A not only physically binds to S1PR2 but also transactivates S1PR2, mainly via its third extracellular loop. For many GPCRs, the third extracellular loop has been proposed to be a ligand binding site [41] and to regulate ligand selectivity and receptor activation [32].

### RhoA-mediated attenuation of RIDD enables gliomas to produce SPARC

Nogo-A inhibits cell migration by activating RhoA, which leads to actomyosin constriction and stress fiber formation [49]. Although glioblastoma cells use myelinated nerve fibers as guide structures for invasion [20], we found that they also activated the anti-migratory Rho-ROCK pathway when exposed to Nogo-A. However, this signaling disturbed the mRNA decay activity of IRE1α, thus enabling glioblastoma cells to increase the production of secreted glycoproteins such as SPARC. This mechanism is similar to the activation of the transcriptional co-factors YAP and TAZ by RhoA-induced cytoskeletal tension [12, 62]. Our finding further expands this concept of mechanotransduced gene regulation [9]: we have identified a fast and dynamic RhoA-driven mechanism that regulates protein translation (yet-unknown mechanisms we termed RAPID). RAPID is efficiently exploited by glioblastoma cells to adapt quickly in a spatiotemporal manner to distinct microenvironments, such as inhibitory white matter.

### The Nogo-A decoy SPARC competes with S1PR2 for binding to an IDR of the Δ20 domain

SPARC modulates the cellular microenvironment and is secreted by cells under stress conditions [5]. We found that SPARC competes with S1PR2 for binding to an IDR of the Nogo-A-Δ20 domain. IDRs allow proteins to interact with different binding partners due to enhanced structural flexibility [10]. Our computational modeling suggested that the flexibility of the Nogo-A-Δ20 IDR enabled its folding into a β-strand, the predominant conformation adopted by protein interfaces that interact with Kazal-like motifs. Thus, Nogo-A-Δ20 was prevented from binding to SPARC when the Kazal-like motif was deleted. Similarly, the IDR of the HIF-1α transactivation domain exhibits comparable backbone flexibility: the domain folds as a helix to bind to the translin-associated zinc finger protein TAZ1, whereas it adopts a β-strand conformation when binding to the asparagine hydroxylase FIH [13]. Glioblastoma cells seem to exploit the structural flexibility of Nogo-A-Δ20 using SPARC as a soluble decoy to attenuate the activation of inhibitory RhoA signaling via S1PR2. In an analogous triadic signaling system, osteoclasts mature upon binding of receptor activator of NF-κB (RANK) to the osteoblast-expressed RANK ligand (RANKL). Osteoblasts, in turn, can interrupt RANK activation by secreting the decoy osteoprotegerin, which neutralizes RANKL and thereby inhibits osteoclast maturation [31]. Previous neuropathological studies hypothesized that invasive glioblastoma cells must possess an intrinsic capability to infiltrate white matter despite its anti-migratory features [17], in addition to mechanisms involving microvessels [2] and ultra-long astrocytoma membrane protrusions as routes for brain invasion [40, 58] as well as mitogenic factors such as neuroligin-3 [54, 55]. We propose that glioblastoma cells counteract the Nogo-A-enforced block in migration. These cells have the intrinsic ability to secrete SPARC when exposed to mechanical stress, such as that caused by RhoA-induced actomyosin constriction, which enables them to migrate away from the cell-dense and nutrition-deprived bulk of the tumor. In turn, this consequently raises the question if less invasive gliomas, e.g., pilocytic astrocytomas or ependymomas lack diffuse infiltrative growth capacities because they do not have the intrinsic ability to secrete SPARC. Solving this question, however, requires further experimental studies.

### SPARC depletion reduces white matter invasion and prolongs survival in an orthotopic glioma model in vivo

Quantification of single glioblastoma cells in various regions of the entire undissected rodent brain has been technically challenging and time consuming so far. With the advancement of our recently developed UM-imaging platform [2], this study provides a new methodology for precisely investigating the invasion of single glioblastoma cells into white-matter structures. Together with the in vitro data, our UM invasion analysis demonstrated that increased AKT-driven ENTPD5 expression enabled glioblastoma cells to produce the Nogo-A decoy SPARC, which was key for their ability to invade the extensively myelinated *corpus callosum*. Thus, gliomas utilize an AKT/ENTPD5-mediated metabolic shift, similar to prostate and lung cancers [7, 14]. However, this altered metabolism not only spurs the growth of cancer cells [26] but also increases their ability to invade the microenvironment. Our work supports recent studies highlighting the important role of altered cell metabolism in controlling cancer invasion and metastasis [22, 35].

The invasive capacity of glioblastomas is a major clinical burden for disease control and therapy [46]. As evidenced by butterfly glioblastomas, in which tumor cells cross the midline via the extensively myelinated *corpus callosum* [17], anti-invasive treatment strategies for gliomas must consider the invasion of the white matter, which represents a substantial proportion of the brain [21]. We demonstrated that SPARC depletion in gliomas with elevated AKT activity improved the response to TMZ treatment and prolonged survival. The reduction of the invasive potential may increase the sensitivity of glioblastoma cells to alkylating drugs. Studies showed that reducing the invasive capability of glioblastoma cells shifts their cell fate decision towards cell growth, which renders them more susceptible to cytostatic therapy [16, 24, 28, 53]. Our pre-clinical data are further supported by patient glioma samples, in which high AKT levels were associated with strong SPARC expression in tumor cells within both the invasive zone and the tumor bulk. Clinical data show that glioblastomas can acquire elevated AKT levels independent of their IDH mutation status [34]. Thus, the molecular mechanism we discovered may apply to both IDH-wildtype and IDH-mutant glioblastomas [34], influencing SPARC production via either IRE1α or AKT/ENTPD5 might represent a worthwhile therapeutic option to pursue.

Taken together, we have identified a novel RhoA-induced signaling mechanism that enables glioma cells to efficiently invade the white matter of the healthy brain using myelinated nerve fibers as guide structures. When glioma cells invade along myelinated nerve fibers, the inhibitory myelin protein Nogo-A activates the S1PR2 receptor, which induces anti-migratory Rho-ROCK signaling. However, these signaling events also lead to deactivation of the ribonuclease IRE1α, which abolishes SPARC mRNA decay and allows glioma cells to secrete SPARC. As a result, SPARC prevents Nogo-A from further activating S1PR2 and thus enables glioblastoma cells to invade white matter. Upon quantifying the invasion along myelinated nerve fiber projections, we found that glioma cells with high levels of p-AKT1 and ENTPD5 were superior at invading white matter due to an increased protein folding capacity. In pre-clinical mouse glioma models, we demonstrated that depleting SPARC and thus preventing white-matter invasion significantly prolonged survival and improved the treatment response to TMZ.

## Electronic supplementary material

Below is the link to the electronic supplementary material.

Supplementary material 1 (PDF 1075 kb)

Supplementary material 2 (PDF 3144 kb)

Supplementary material 3 (PDF 762 kb)

Supplementary material 4 (PDF 11673 kb)

Supplementary material 5 (PDF 1584 kb)

Supplementary material 6 (PDF 1029 kb)

Supplementary material 7 (PDF 528 kb)

Supplementary material 8 (PDF 3341 kb)

Supplementary material 9 (XLSX 67 kb)

Supplementary material 10 (XLSX 15 kb)

Supplementary material 11 (DOCX 86 kb)

Supplementary material 12 (MPEG 7834 kb)

Supplementary material 13 (MPEG 6976 kb)

## References

[1] Bady P, Diserens AC, Castella V, Kalt S, Heinimann K, Hamou MF (2012). DNA fingerprinting of glioma cell lines and considerations on similarity measurements. Neuro Oncol.

[2] Breckwoldt MO, Bode J, Kurz FT, Hoffmann A, Ochs K, Ott M (2016). Correlated magnetic resonance imaging and ultramicroscopy (MR-UM) is a tool kit to assess the dynamics of glioma angiogenesis. Elife.

[3] Caroni P, Schwab ME (1988). Antibody against myelin-associated inhibitor of neurite growth neutralizes nonpermissive substrate properties of CNS white matter. Neuron.

[4] Chalhoub N, Baker SJ (2009). PTEN and the PI3-kinase pathway in cancer. Ann Rev Pathol.

[5] Chlenski A, Cohn SL (2010). Modulation of matrix remodeling by SPARC in neoplastic progression. Semin Cell Dev Biol.

[6] Claes A, Idema AJ, Wesseling P (2007). Diffuse glioma growth: a guerilla war. Acta Neuropathol.

[7] Curry NL, Mino-Kenudson M, Oliver TG, Yilmaz OH, Yilmaz VO, Moon JY (2013). Pten-null tumors cohabiting the same lung display differential AKT activation and sensitivity to dietary restriction. Cancer Discov.

[8] Dejeans N, Pluquet O, Lhomond S, Grise F, Bouchecareilh M, Juin A (2012). Autocrine control of glioma cells adhesion and migration through IRE1alpha-mediated cleavage of SPARC mRNA. J Cell Sci.

[9] DuFort CC, Paszek MJ, Weaver VM (2011). Balancing forces: architectural control of mechanotransduction. Nat Rev Mol Cell Biol.

[10] Dunker AK, Silman I, Uversky VN, Sussman JL (2008). Function and structure of inherently disordered proteins. Curr Opin Struct Biol.

[11] Dunn GP, Rinne ML, Wykosky J, Genovese G, Quayle SN, Dunn IF (2012). Emerging insights into the molecular and cellular basis of glioblastoma. Genes Dev.

[12] Dupont S, Morsut L, Aragona M, Enzo E, Giulitti S, Cordenonsi M (2011). Role of YAP/TAZ in mechanotransduction. Nature.

[13] Dyson HJ (2016). Making sense of intrinsically disordered proteins. Biophys J.

[14] Fang M, Shen Z, Huang S, Zhao L, Chen S, Mak TW (2010). The ER UDPase ENTPD5 promotes protein N-glycosylation, the Warburg effect, and proliferation in the PTEN pathway. Cell.

[15] Felsberg J, Hentschel B, Kaulich K, Gramatzki D, Zacher A, Malzkorn B (2017). Epidermal growth factor receptor variant III (EGFRvIII) positivity in EGFR-amplified glioblastomas: prognostic role and comparison between primary and recurrent tumors. Clin Cancer Res.

[16] Gerlee P, Nelander S (2016). Travelling wave analysis of a mathematical model of glioblastoma growth. Math Biosci.

[17] Giese A, Bjerkvig R, Berens ME, Westphal M (2003). Cost of migration: invasion of malignant gliomas and implications for treatment. J Clin Oncol.

[18] Giese A, Kluwe L, Laube B, Meissner H, Berens ME, Westphal M (1996). Migration of human glioma cells on myelin. Neurosurgery.

[19] GrandPre T, Li S, Strittmatter SM (2002). Nogo-66 receptor antagonist peptide promotes axonal regeneration. Nature.

[20] Gritsenko PG, Ilina O, Friedl P (2012). Interstitial guidance of cancer invasion. J Pathol.

[21] Harris JJ, Jolivet R, Attwell D (2012). Synaptic energy use and supply. Neuron.

[22] Hay N (2016). Reprogramming glucose metabolism in cancer: can it be exploited for cancer therapy?. Nat Rev Cancer.

[23] He Y, Beatty A, Han X, Ji Y, Ma X, Adelstein RS (2012). Nonmuscle myosin IIB links cytoskeleton to IRE1alpha signaling during ER stress. Dev Cell.

[24] Hill R, Murray SA, Maherally Z, Higgins SC, Pilkington GJ, Tivnan A (2016). Drug repurposing to circumvent chemotherapy resistance in brain tumours. Resistance to targeted therapies against adult brain cancers.

[25] Hollien J, Weissman JS (2006). Decay of endoplasmic reticulum-localized mRNAs during the unfolded protein response. Science.

[26] Israelsen WJ, Vander Heiden MG (2010). ATP consumption promotes cancer metabolism. Cell.

[27] Karcher S, Steiner HH, Ahmadi R, Zoubaa S, Vasvari G, Bauer H (2006). Different angiogenic phenotypes in primary and secondary glioblastomas. Int J Cancer.

[28] Kathagen A, Schulte A, Balcke G, Phillips HS, Martens T, Matschke J (2013). Hypoxia and oxygenation induce a metabolic switch between pentose phosphate pathway and glycolysis in glioma stem-like cells. Acta Neuropathol.

[29] Kempf A, Tews B, Arzt ME, Weinmann O, Obermair FJ, Pernet V (2014). The sphingolipid receptor S1PR2 is a receptor for Nogo—a repressing synaptic plasticity. PLoS Biol.

[30] Korennykh AV, Egea PF, Korostelev AA, Finer-Moore J, Zhang C, Shokat KM (2009). The unfolded protein response signals through high-order assembly of Ire1. Nature.

[31] Lacey DL, Boyle WJ, Simonet WS, Kostenuik PJ, Dougall WC, Sullivan JK (2012). Bench to bedside: elucidation of the OPG-RANK-RANKL pathway and the development of denosumab. Nat Rev Drug Discov.

[32] Lawson Z, Wheatley M (2004). The third extracellular loop of G-protein-coupled receptors: more than just a linker between two important transmembrane helices. Biochem Soc Trans.

[33] Li Y, Guessous F, Kwon S, Kumar M, Ibidapo O, Fuller L (2008). PTEN has tumor-promoting properties in the setting of gain-of-function p53 mutations. Cancer Res.

[34] Louis DN, Perry A, Reifenberger G, von Deimling A, Figarella-Branger D, Cavenee WK (2016). The 2016 World Health Organization classification of tumors of the central nervous system: a summary. Acta Neuropathol.

[35] Lunt SY, Vander Heiden MG (2011). Aerobic glycolysis: meeting the metabolic requirements of cell proliferation. Ann Rev Cell Dev Biol.

[36] McBeath R, Pirone DM, Nelson CM, Bhadriraju K, Chen CS (2004). Cell shape, cytoskeletal tension, and RhoA regulate stem cell lineage commitment. Dev Cell.

[37] Muppidi JR, Schmitz R, Green JA, Xiao W, Larsen AB, Braun SE (2014). Loss of signalling via Galpha13 in germinal centre B-cell-derived lymphoma. Nature.

[38] Mutoh T, Rivera R, Chun J (2012). Insights into the pharmacological relevance of lysophospholipid receptors. Br J Pharmacol.

[39] Oikawa D, Tokuda M, Hosoda A, Iwawaki T (2010). Identification of a consensus element recognized and cleaved by IRE1 alpha. Nucleic Acids Res.

[40] Osswald M, Jung E, Sahm F, Solecki G, Venkataramani V, Blaes J (2015). Brain tumour cells interconnect to a functional and resistant network. Nature.

[41] Peeters MC, van Westen GJ, Li Q, IJzerman AP (2011). Importance of the extracellular loops in G protein-coupled receptors for ligand recognition and receptor activation. Trends Pharmacol Sci.

[42] Phillips E, Lang V, Bohlen J, Bethke F, Puccio L, Tichy D (2016). Targeting atypical protein kinase C iota reduces viability in glioblastoma stem-like cells via a notch signaling mechanism. Int J Cancer.

[43] Preusser M, de Ribaupierre S, Wohrer A, Erridge SC, Hegi M, Weller M (2011). Current concepts and management of glioblastoma. Ann Neurol.

[44] Pyne NJ, Pyne S (2010). Sphingosine 1-phosphate and cancer. Nat Rev Cancer.

[45] Rempel SA, Golembieski WA, Ge S, Lemke N, Elisevich K, Mikkelsen T (1998). SPARC: a signal of astrocytic neoplastic transformation and reactive response in human primary and xenograft gliomas. J Neuropathol Exp Neurol.

[46] Ricard D, Idbaih A, Ducray F, Lahutte M, Hoang-Xuan K, Delattre JY (2012). Primary brain tumours in adults. Lancet.

[47] Schultz C, Lemke N, Ge S, Golembieski WA, Rempel SA (2002). Secreted protein acidic and rich in cysteine promotes glioma invasion and delays tumor growth in vivo. Cancer Res.

[48] Schwab ME (2010). Functions of Nogo proteins and their receptors in the nervous system. Nat Rev Neurosci.

[49] Schwab ME, Strittmatter SM (2014). Nogo limits neural plasticity and recovery from injury. Curr Opin Neurobiol.

[50] Simonen M, Pedersen V, Weinmann O, Schnell L, Buss A, Ledermann B (2003). Systemic deletion of the myelin-associated outgrowth inhibitor Nogo—a improves regenerative and plastic responses after spinal cord injury. Neuron.

[51] Tojkander S, Gateva G, Lappalainen P (2012). Actin stress fibers–assembly, dynamics and biological roles. J Cell Sci.

[52] Tompa P, Fuxreiter M, Oldfield CJ, Simon I, Dunker AK, Uversky VN (2009). Close encounters of the third kind: disordered domains and the interactions of proteins. BioEssays.

[53] Venere M, Horbinski C, Crish JF, Jin X, Vasanji A, Major J (2015). The mitotic kinesin KIF11 is a driver of invasion, proliferation, and self-renewal in glioblastoma. Sci Transl Med.

[54] Venkatesh HS, Johung TB, Caretti V, Noll A, Tang Y, Nagaraja S (2015). Neuronal activity promotes glioma growth through neuroligin-3 secretion. Cell.

[55] Venkatesh HS, Tam LT, Woo PJ, Lennon J, Nagaraja S, Gillespie SM (2017). Targeting neuronal activity-regulated neuroligin-3 dependency in high-grade glioma. Nature.

[56] Vicente-Manzanares M, Ma X, Adelstein RS, Horwitz AR (2009). Non-muscle myosin II takes centre stage in cell adhesion and migration. Nat Rev Mol Cell Biol.

[57] Wang X, Chun SJ, Treloar H, Vartanian T, Greer CA, Strittmatter SM (2002). Localization of Nogo-A and Nogo-66 receptor proteins at sites of axon-myelin and synaptic contact. J Neurosci.

[58] Weil S, Osswald M, Solecki G, Grosch J, Jung E, Lemke D (2017). Tumor microtubes convey resistance to surgical lesions and chemotherapy in gliomas. Neuro Oncol.

[59] Weller M, van den Bent M, Tonn JC, Stupp R, Preusser M, Cohen-Jonathan-Moyal E (2017). European Association for Neuro-Oncology (EANO) guideline on the diagnosis and treatment of adult astrocytic and oligodendroglial gliomas. Lancet Oncol.

[60] Wong GS, Rustgi AK (2013). Matricellular proteins: priming the tumour microenvironment for cancer development and metastasis. Br J Cancer.

[61] Xiong NX, Zhao HY, Zhang FC, He ZQ (2007). Negative correlation of Nogo-A with the malignancy of oligodendroglial tumor. Neurosci Bull.

[62] Yu FX, Zhao B, Panupinthu N, Jewell JL, Lian I, Wang LH (2012). Regulation of the Hippo-YAP pathway by G-protein-coupled receptor signaling. Cell.

[63] Zacher A, Kaulich K, Stepanow S, Wolter M, Kohrer K, Felsberg J (2017). Molecular diagnostics of gliomas using next generation sequencing of a glioma-tailored gene panel. Brain Pathol.

