# Supplementary material for: RhoA regulates translation of the Nogo-A decoy SPARC in white matter-invading glioblastomas
Source: Acta Neuropathol. 2019 May 6;138(2):275–93. doi: 10.1007/s00401-019-02021-z (PMC6660512; doi:10.1007/s00401-019-02021-z)
Supplement: Supplementary file 1 — Supplementary material 1 (PDF 1075 kb) [file 401_2019_2021_MOESM1_ESM.pdf]

SUPPLEMENTAL FIGURE 1

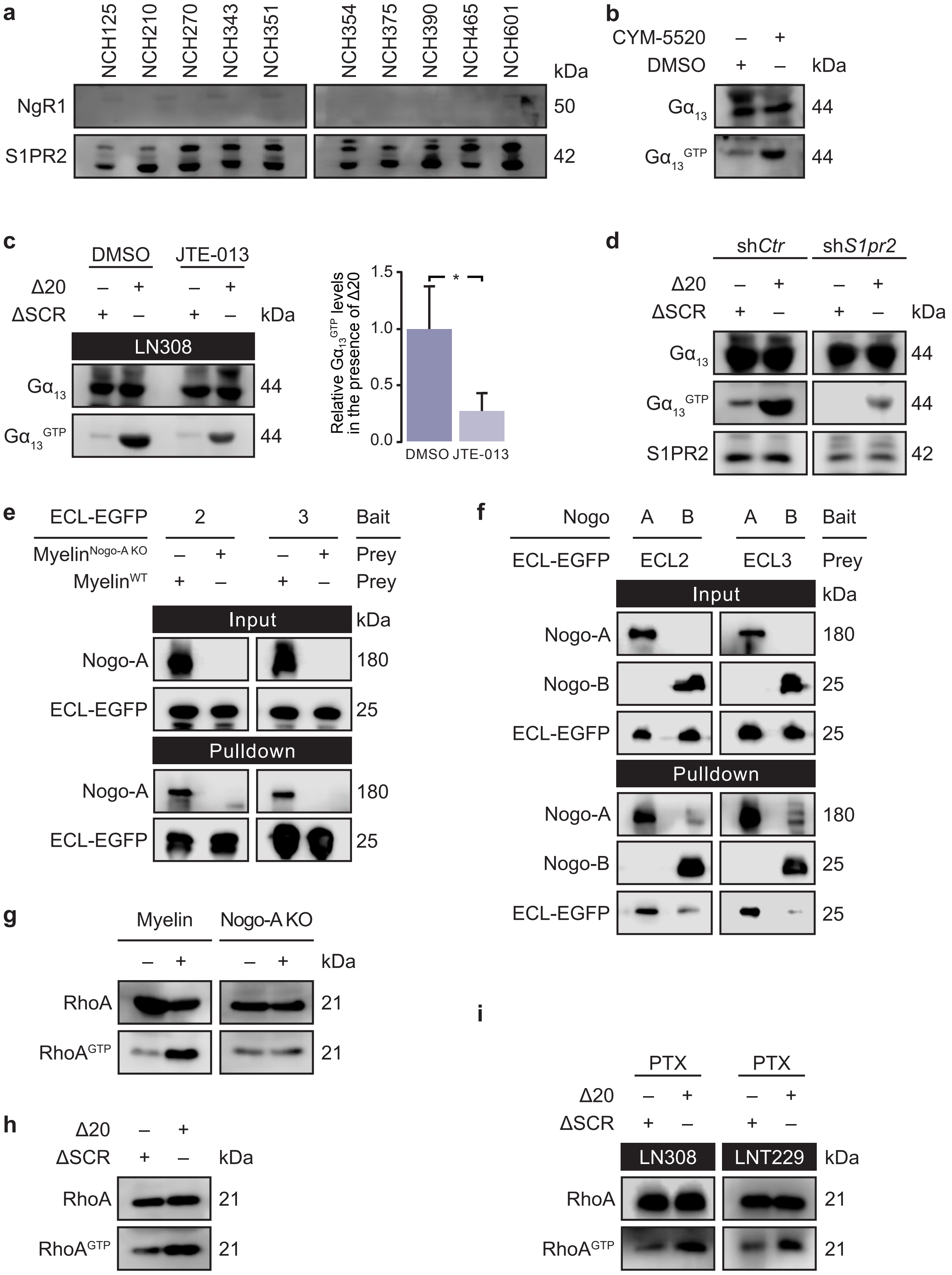

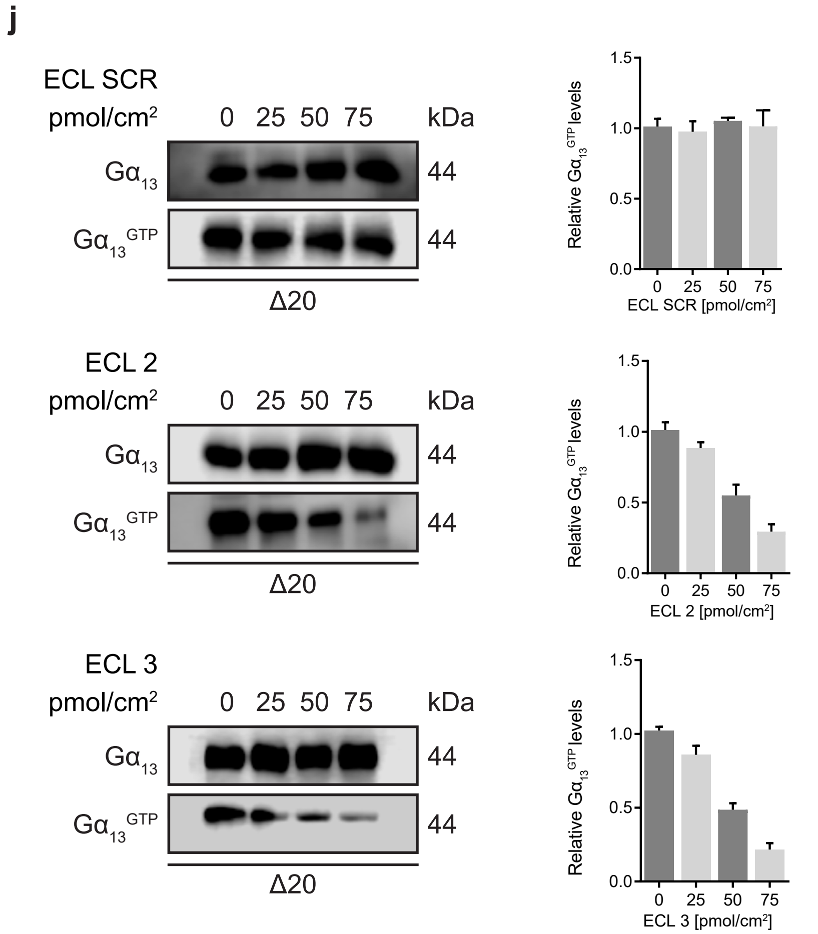

Figure S1. Glioma cells respond to Nogo-A by activating S1PR2 and its downstream effector RhoA. Related to Figure 1.

(a) S1PR2 and NgR1 levels of low passage patient-derived glioma cells. (b, c, d) $G\alpha_{13}^{GTP}$ levels of (b) LN308 glioma cells treated with 5 µM CYM-5520, (c) LN308 cells treated with 1 µM JTE-013, (d) NIH-3T3 fibroblasts expressing control shRNA (sh*Ctr*) or shRNA against *S1pr2* (sh*S1pr2*). (c, d) Nogo-A-Δ20 (Δ20) or Nogo-A-ΔSCR (ΔSCR). (e, f) Pulldown using (e) either EGFP-ECL2/3 and myelin extracts or (f) using His-tagged Nogo-A or Nogo-B for precipitating EGFP-ECL2/3 expressed in HEK293 cells. (g, h, i) $RhoA^{GTP}$ levels of LN308 glioma cells (g) in the presence of myelin extracted from wildtype or Nogo-A KO mice, (h) in the presence of Nogo-A-Δ20 or Nogo-A-ΔSCR. (i) Glioma cells treated with pertussis toxin (PTX). (j) LN308 cells treated with blocking peptides mimicking ECL2 or ECL3 or a scrambled peptide (SCR) at serial dilutions. Error bars represent the SD, n = 3. Unpaired t-test, * $p \leq 0.05$; ** $p \leq 0.01$; *** $p \leq 0.001$; not significant = $p > 0.05$.
