# Supplementary material for: RhoA regulates translation of the Nogo-A decoy SPARC in white matter-invading glioblastomas
Source: Acta Neuropathol. 2019 May 6;138(2):275–93. doi: 10.1007/s00401-019-02021-z (PMC6660512; doi:10.1007/s00401-019-02021-z)
Supplement: Supplementary file 2 — Supplementary material 2 (PDF 3144 kb) [file 401_2019_2021_MOESM2_ESM.pdf]

SUPPLEMENTAL FIGURE 2

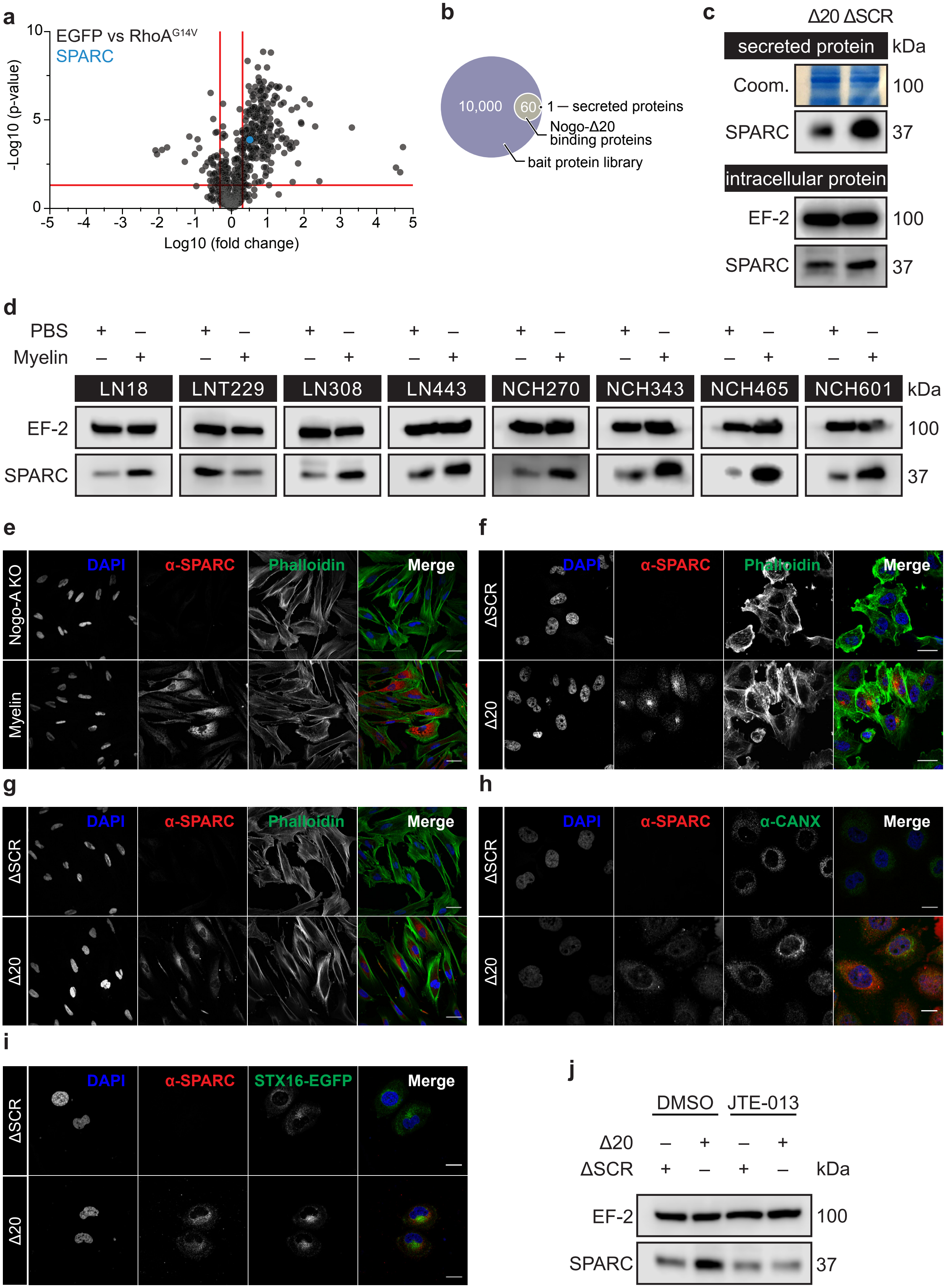

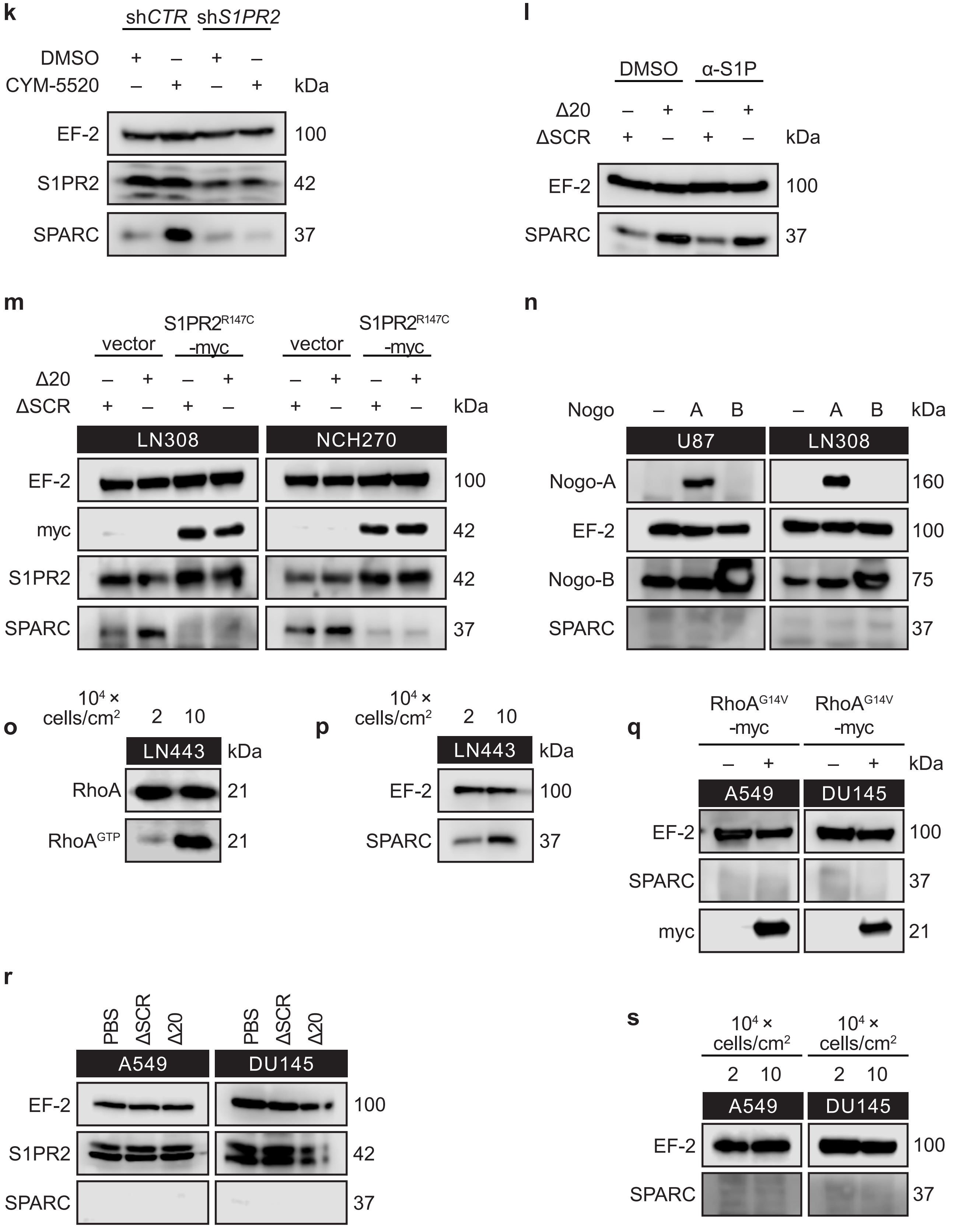
k
shCTR shS1PR2
DMSO + – + –
CYM-5520 – + – + kDa
EF-2 100
S1PR2 42
SPARC 37
l
DMSO α-S1P
Δ20 – + – +
ΔSCR + – + – kDa
EF-2 100
SPARC 37
m
vector S1PR2R147C -myc vector S1PR2R147C -myc
Δ20 – + – + – + – +
ΔSCR + – + – + – + – kDa
LN308 NCH270
EF-2 100
myc 42
S1PR2 42
SPARC 37
n
Nogo – A B – A B kDa
U87 LN308
Nogo-A 160
EF-2 100
Nogo-B 75
SPARC 37
o
10⁴ × cells/cm² 2 10
LN443 kDa
RhoA 21
RhoAGTP 21
p
10⁴ × cells/cm² 2 10
LN443 kDa
EF-2 100
SPARC 37
q
RhoAG14V -myc RhoAG14V -myc
– + – + kDa
A549 DU145
EF-2 100
SPARC 37
myc 21
r
PBS ΔSCR Δ20 PBS ΔSCR Δ20
A549 DU145
EF-2 100
S1PR2 42
SPARC 37
s
10⁴ × cells/cm² 10⁴ × cells/cm²
2 10 2 10
A549 DU145
EF-2 100
SPARC 37

Figure S2. Glioma cells secrete the Nogo-A decoy SPARC upon RhoA activation. Related to Figure 2.
(a) Nano-LC-MS/MS of secreted proteins from LN308 cells. Averaged and normalized abundances of detected proteins calculated as ratio between EGFP- and $RhoA^{G14V}$-expressing cells. (b) Candidate overlap-analysis between a previously conducted Y2H screen (Kempf et al., 2014) and the Nano-LC-MS/MS. (c) SPARC levels in secreted and in intracellular protein isolates from LN308 cells. (d) SPARC levels in glioma cell lines (LN18, LNT229, LN308, LN443) and in low passage patient-derived glioma cells (NCH342, NCH417, NCH465, NCH601). (e, f, g, h, i) CLSM of (f, h, i) LN308 cells or (e, g) NCH-465 cells grown on protein-coated cover slips. Calnexin (CANX); syntaxin-16 fused to EGFP (STX16-EGFP). (j, k, l, m, n) SPARC levels (j) in LN308 glioma cells treated with 1 µM JTE-013 or (k) treated with 5 µM CYM-5520 for 16 h, or (l) grown in serum-free medium containing 1 µg/ml α-S1P; (m) in LN308 or NCH270 glioma cells expressing myc-tagged $S1PR2^{R147C}$; (n) in U87 or LN308 expressing expressing His-tagged Nogo-A or Nogo-B. (o, p) $RhoA^{GTP}$ or SPARC levels in LN443 cells grown at either low (2×10^4 cells/cm^2) or high (10×10^4 cells/cm^2) density. (q, r, s) SPARC levels in A549 and DU145 cells (q) expressing $RhoA^{G14V}$, (r) grown on protein-coated surfaces or (s) at either low (2×10^4 cells/cm^2) or high (10×10^4 cells/cm^2) density. (c, d, e, f, g, h, i, j, l, m, r) Cells were exposed to myelin extracted from C57BL/6-*Rtn4t*^{m1Schw} mice (Nogo-A KO), Nogo-A-Δ20 (Δ20), or Nogo-A-ΔSCR (ΔSCR) for 16 h. Control shRNA (sh*CTR*); shRNA against *S1PR2* (sh*S1PR2*).
