# Supplementary material for: RhoA regulates translation of the Nogo-A decoy SPARC in white matter-invading glioblastomas
Source: Acta Neuropathol. 2019 May 6;138(2):275–93. doi: 10.1007/s00401-019-02021-z (PMC6660512; doi:10.1007/s00401-019-02021-z)
Supplement: Supplementary file 3 — Supplementary material 3 (PDF 762 kb) [file 401_2019_2021_MOESM3_ESM.pdf]

SUPPLEMENTAL FIGURE 3

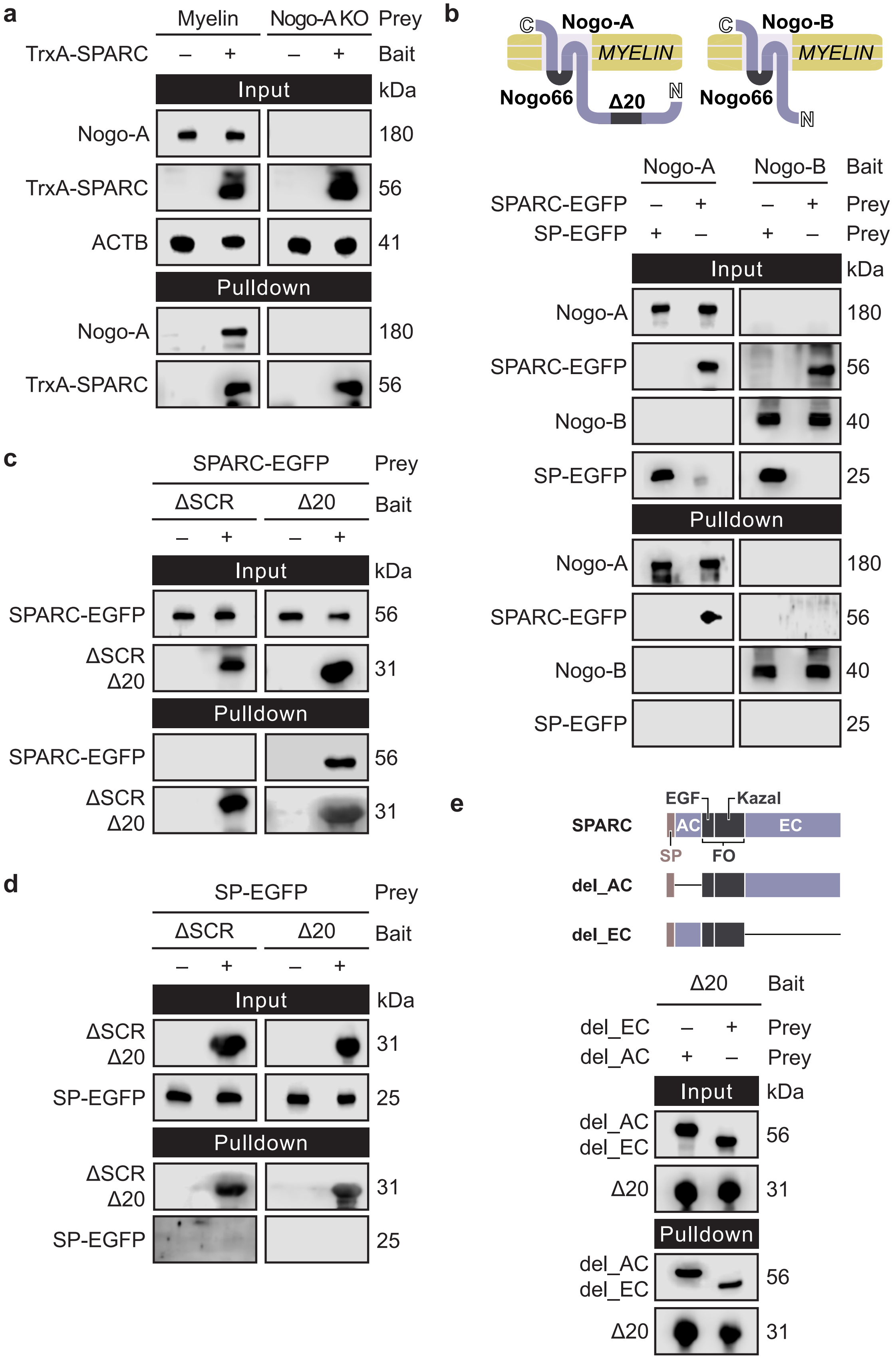

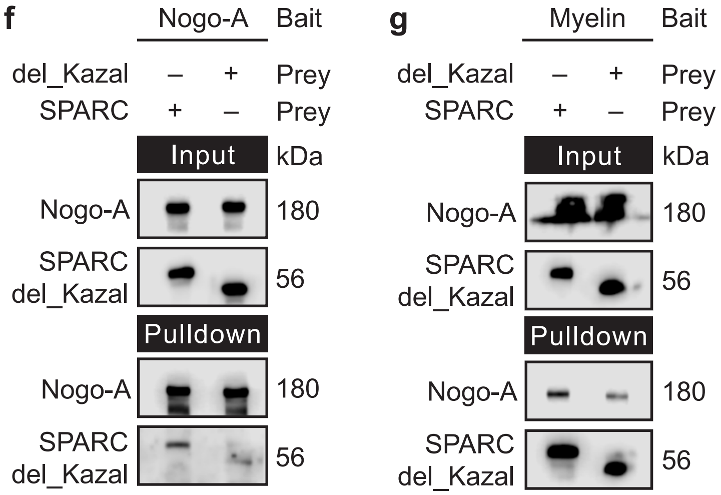

Figure S3. SPARC binds to Nogo-A. Related to Figure 2.
(a) IMAC with thioredoxin A-SPARC fusion protein (TrxA-SPARC) and and myelin extracts from C57BL/6 (Myelin) or C57BL/6-*Rtn4*^{tm1Schw} mice (Nogo-A KO). (b) Domains of Nogo isoforms Nogo-A and Nogo-B. (b, c, d) IMAC using (b) His-tagged Nogo-A or Nogo-B and EGFP-SPARC or SP-EGFP or (c, d) His-tagged Δ20 or ΔSCR and EGFP-tagged SPARC (SPARC-EGFP) or EGFP with an N-terminal signal peptide (SP-EGFP). (e, f, g) IMAC using (e) His-tagged Δ20 and EGFP-tagged SPARC with a deletion of either the acidic domain (del_AC) or the extracellular calcium binding domain (del_EC), (f) His-tagged Nogo-A and EGFP-tagged SPARC or del_Kazal, or (g) His-tagged SPARC or del_Kazal and myelin extracts from C57BL/6 mice (myelin).
