# Supplementary material for: RhoA regulates translation of the Nogo-A decoy SPARC in white matter-invading glioblastomas
Source: Acta Neuropathol. 2019 May 6;138(2):275–93. doi: 10.1007/s00401-019-02021-z (PMC6660512; doi:10.1007/s00401-019-02021-z)
Supplement: Supplementary file 4 — Supplementary material 4 (PDF 11673 kb) [file 401_2019_2021_MOESM4_ESM.pdf]

SUPPLEMENTAL FIGURE 4

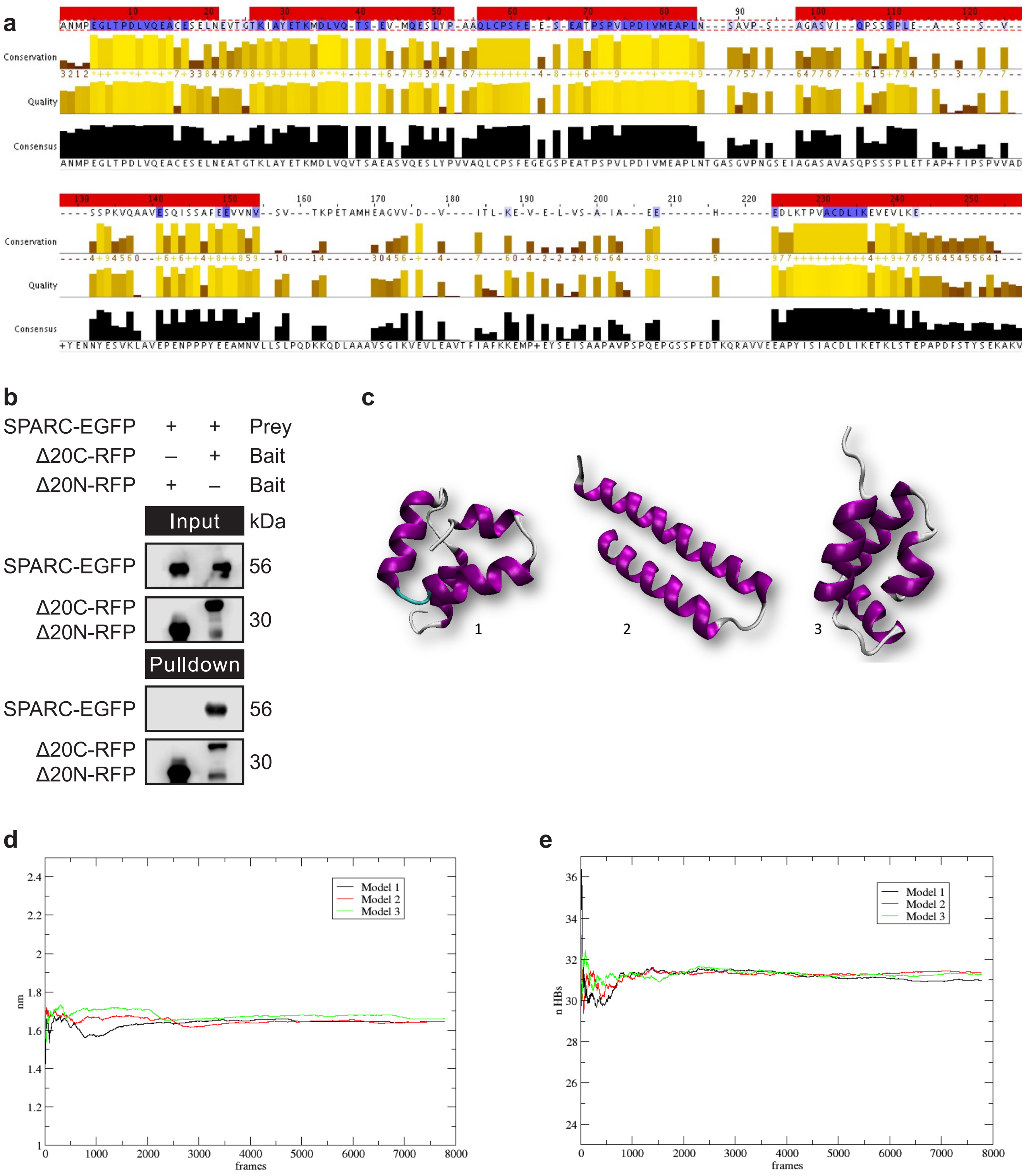

f

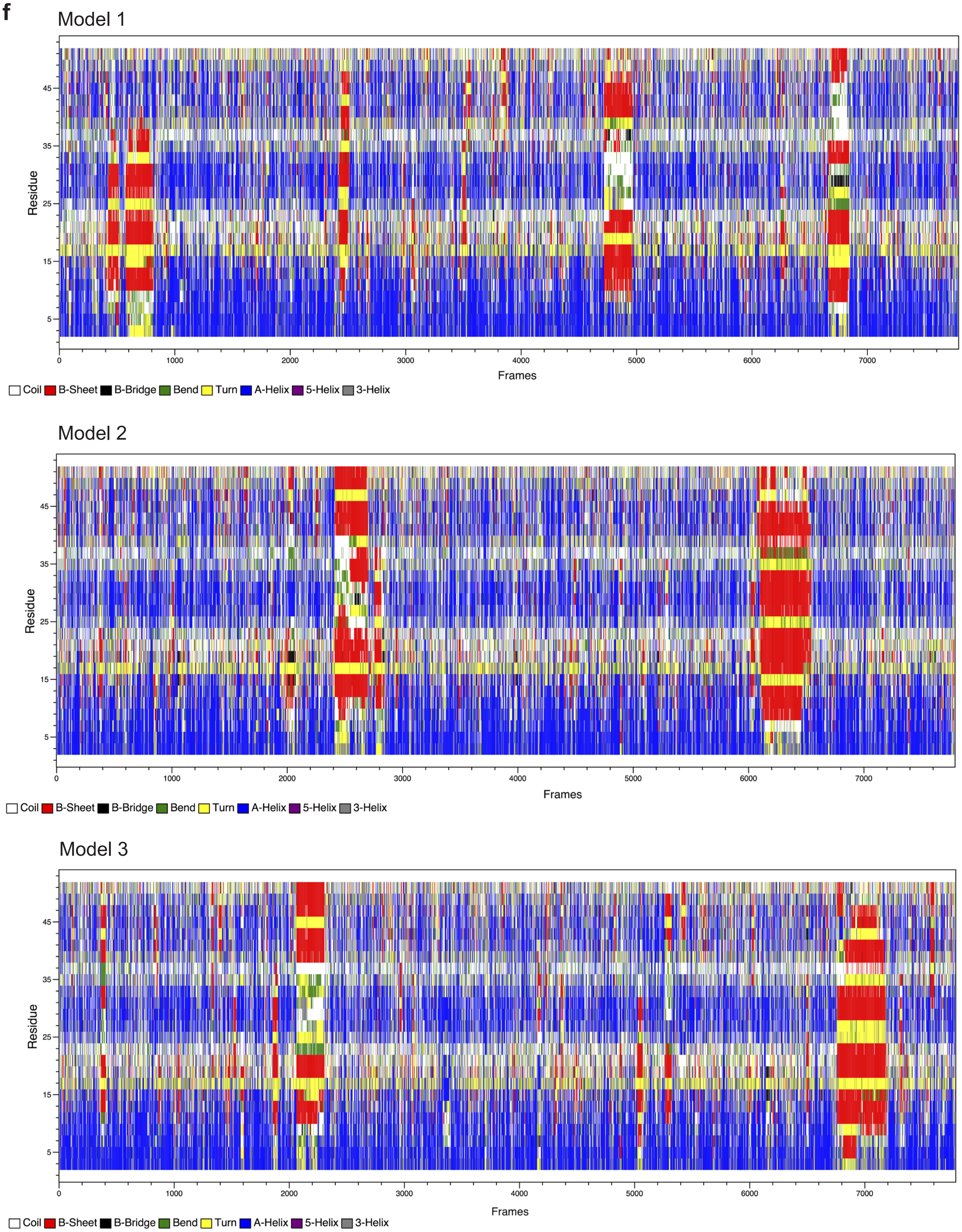
Model 1
Residue
45
35
25
15
5
0
1000
2000
3000
4000
5000
6000
7000
Frames
Coil B-Sheet B-Bridge Bend Turn A-Helix 5-Helix 3-Helix
Model 2
Residue
45
35
25
15
5
0
1000
2000
3000
4000
5000
6000
7000
Frames
Coil B-Sheet B-Bridge Bend Turn A-Helix 5-Helix 3-Helix
Model 3
Residue
45
35
25
15
5
0
1000
2000
3000
4000
5000
6000
7000
Frames
Coil B-Sheet B-Bridge Bend Turn A-Helix 5-Helix 3-Helix

g

Model	Coil	β-Sheet	β-Bridge	Bend	Turn	α-helix	5-helix	3-helix	TOT-structure
1	0.27	0.08	0.01	0.08	0.12	0.4	0	0.04	0.61
2	0.26	0.09	0.01	0.08	0.12	0.4	0	0.04	0.62
3	0.27	0.09	0.01	0.08	0.12	0.4	0	0.04	0.62

h

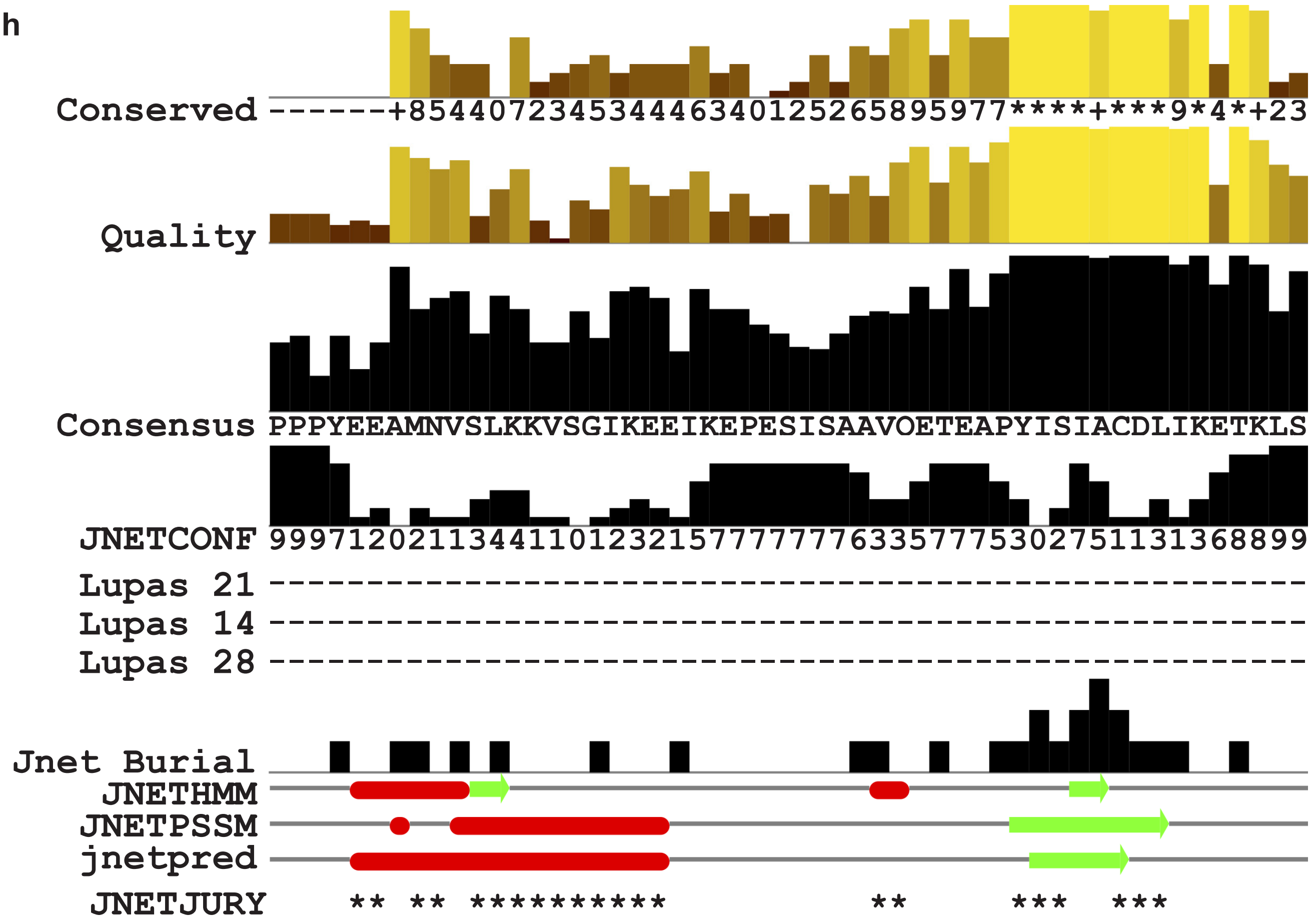

i

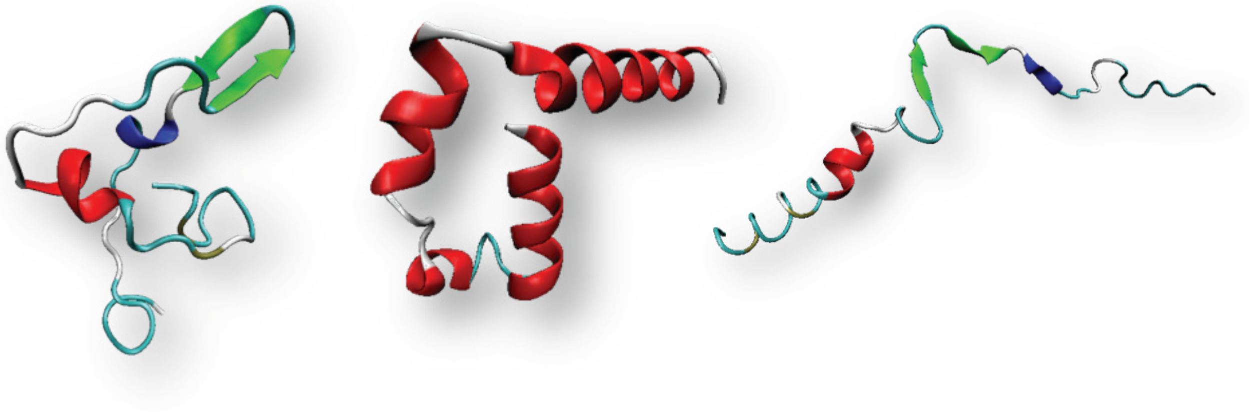

j

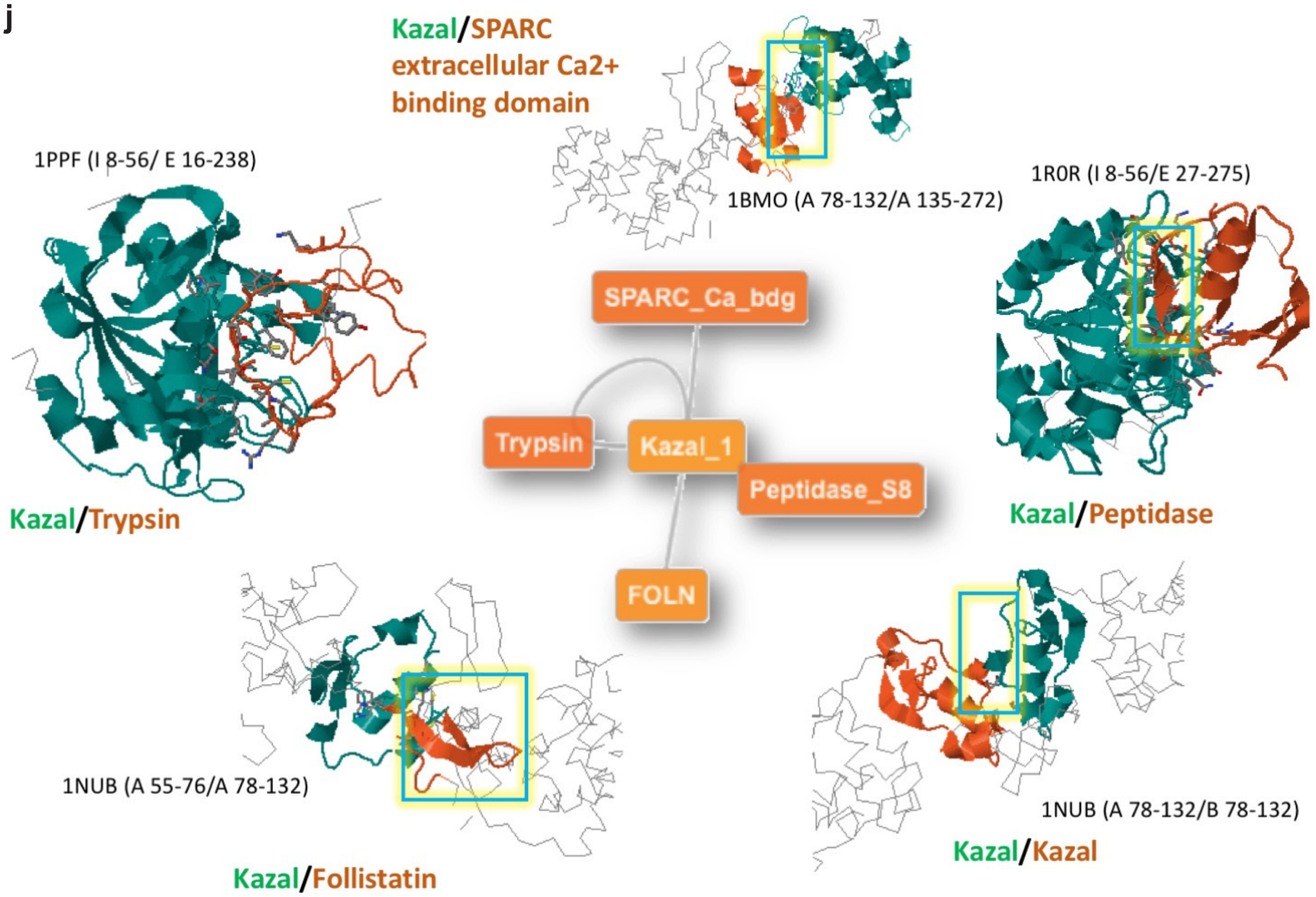

Kazal/SPARC extracellular Ca2+ binding domain
1BMO (A 78-132/A 135-272)
1PPF (I 8-56/ E 16-238)
Kazal/Trypsin
1R0R (I 8-56/E 27-275)
Kazal/Peptidase
SPARC_Ca_bdg
Trypsin
Kazal_1
Peptidase_S8
FOLN
1NUB (A 55-76/A 78-132)
Kazal/Follistatin
1NUB (A 78-132/B 78-132)
Kazal/Kazal

k

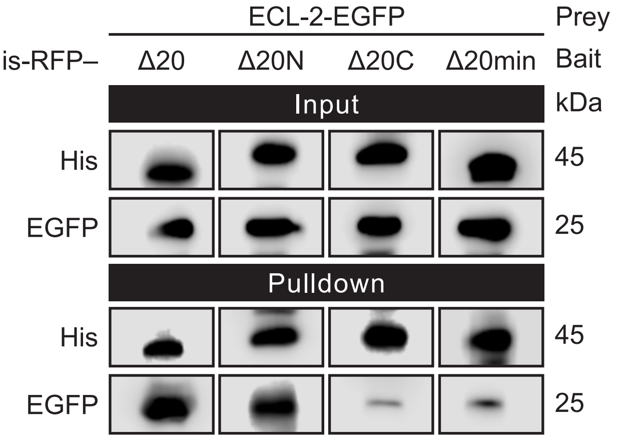

ECL-2-EGFP
Prey
His-RFP–
Δ20
Δ20N
Δ20C
Δ20min
Bait
Input
kDa
His
45
EGFP
25
Pulldown
His
45
EGFP
25

l

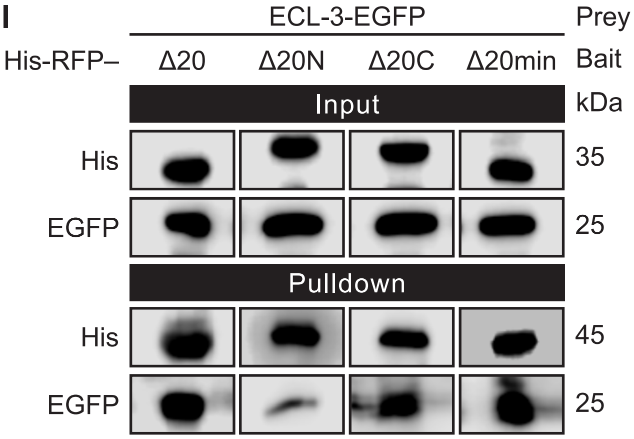

ECL-3-EGFP
Prey
His-RFP–
Δ20
Δ20N
Δ20C
Δ20min
Bait
Input
kDa
His
35
EGFP
25
Pulldown
His
45
EGFP
25

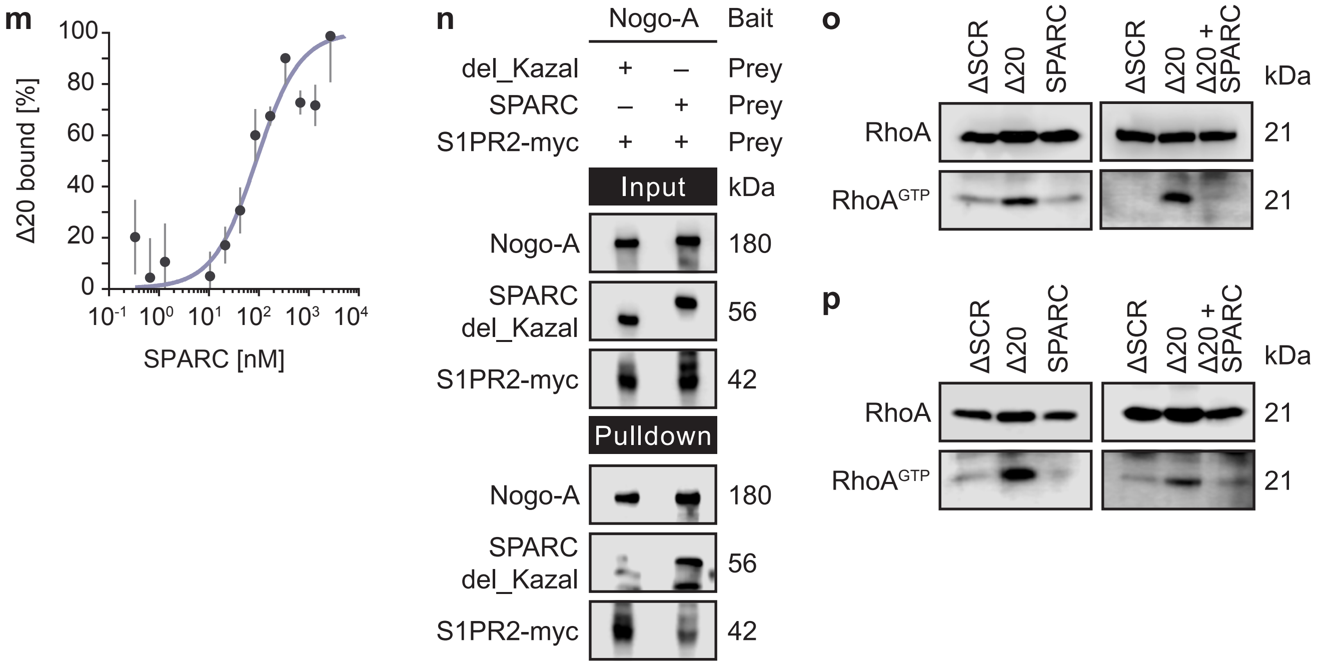

Figure S4. A minimal disordered region of Nogo-A-Δ20 is responsible for binding both S1PR2 and SPARC. Related to Figure 2.

(a) Sequence analysis of Nogo-A-Δ20. Red bars indicate residues that are conserved across most homologs. The sequence coloring indicates identity among homologs: white <40%; light gray > 40%; light blue >60%; medium blue >80%. The conversation shows conserved physicochemical properties. Quality indicates the ratio of two BLOSUM 62 scores for a mutation pair and the conserved BLOSUM 62 score of each residue. The consensus shows the most common residues and their percentage. (b) IMAC with Nogo-A-Δ20N-RFP (Δ20N-RFP) or Nogo-A-Δ20C-RFP (Δ20C-RFP) and EGFP-tagged SPARC (SPARC-EGFP). (c) Best *ab initio* models of Nogo-A-Δ20min in terms of DOPE score and stereochemical quality. (d) Cumulative averages of Rg for Nogo-A-Δ20min. (e) Cumulative averages of hydrogen bonds for Nogo-A-Δ20min. (f, g) The secondary structure of models 1, 2 and 3 across all ensembles. TOT structure = α-helix + β-Sheet + β-bridge + turn. (h) Primary sequence conservation and secondary structure prediction for Nogo-A-Δ20min (Δ20min) using Jpred4. Lupas 21, Lupas 14 and Lupas 28 are coiled-coil sequence predictions. Jnetpred is a consensus prediction. Helices are marked as red tubes, and sheets are shows as green arrows. JNETHMM: HMM profile-based prediction. Helices are marked as red tubes, and sheets are depicted as green arrows. JNETPSSM: PSSM-based prediction. Helices are marked as red tubes, and sheets are shown as green arrows. JNETJURY: * indicates that the JNETJURY was invoked to rationalize significantly different primary predictions. (i) Representative conformations of the three most populated clusters from REMC simulations using PROFASI. (j) Kazal domain/domain interactions from the database of 3D Interaction Domains. Each interface is shown as a representative structure. PDBID of the representative structure and residues involved in the interaction are shown for each interface. The β-sheet motif is highlighted by a square. (k, l) IMAC using His/RFP-tagged Nogo-A-Δ20 (Δ20), Nogo-A-Δ20N (Δ20N), Nogo-A-Δ20C (Δ20C), Nogo-A-Δ20min (Δ20min) and either EGFP-tagged (k) ECL2 or (l) ECL3. Extracellular loop of S1PR2 (ECL). (m) MST using Δ20 or Nogo66 and SPARC. Error bars represent the SD, $n = 3$. Unpaired t-test, * $p \leq 0.05$; ** $p \leq 0.01$; *** $p \leq 0.001$; not significant = $p > 0.05$. (n) Competitive IMAC using His-tagged Nogo-A and myc-tagged S1PR2 in a mixture containing either EGFP-tagged del_Kazal or SPARC. (o, p) RhoA^{GTP} levels in (o) LN308 cells or (p) LNT229 glioma cells in the presence of ΔSCR, Δ20, ΔSCR/SPARC, or Δ20/SPARC. (e, f, g, m, o, p) Nogo-A-Δ20 (Δ20), Nogo-A-ΔSCR (ΔSCR).
