# Supplementary material for: RhoA regulates translation of the Nogo-A decoy SPARC in white matter-invading glioblastomas
Source: Acta Neuropathol. 2019 May 6;138(2):275–93. doi: 10.1007/s00401-019-02021-z (PMC6660512; doi:10.1007/s00401-019-02021-z)
Supplement: Supplementary file 5 — Supplementary material 5 (PDF 1584 kb) [file 401_2019_2021_MOESM5_ESM.pdf]

SUPPLEMENTAL FIGURE 5

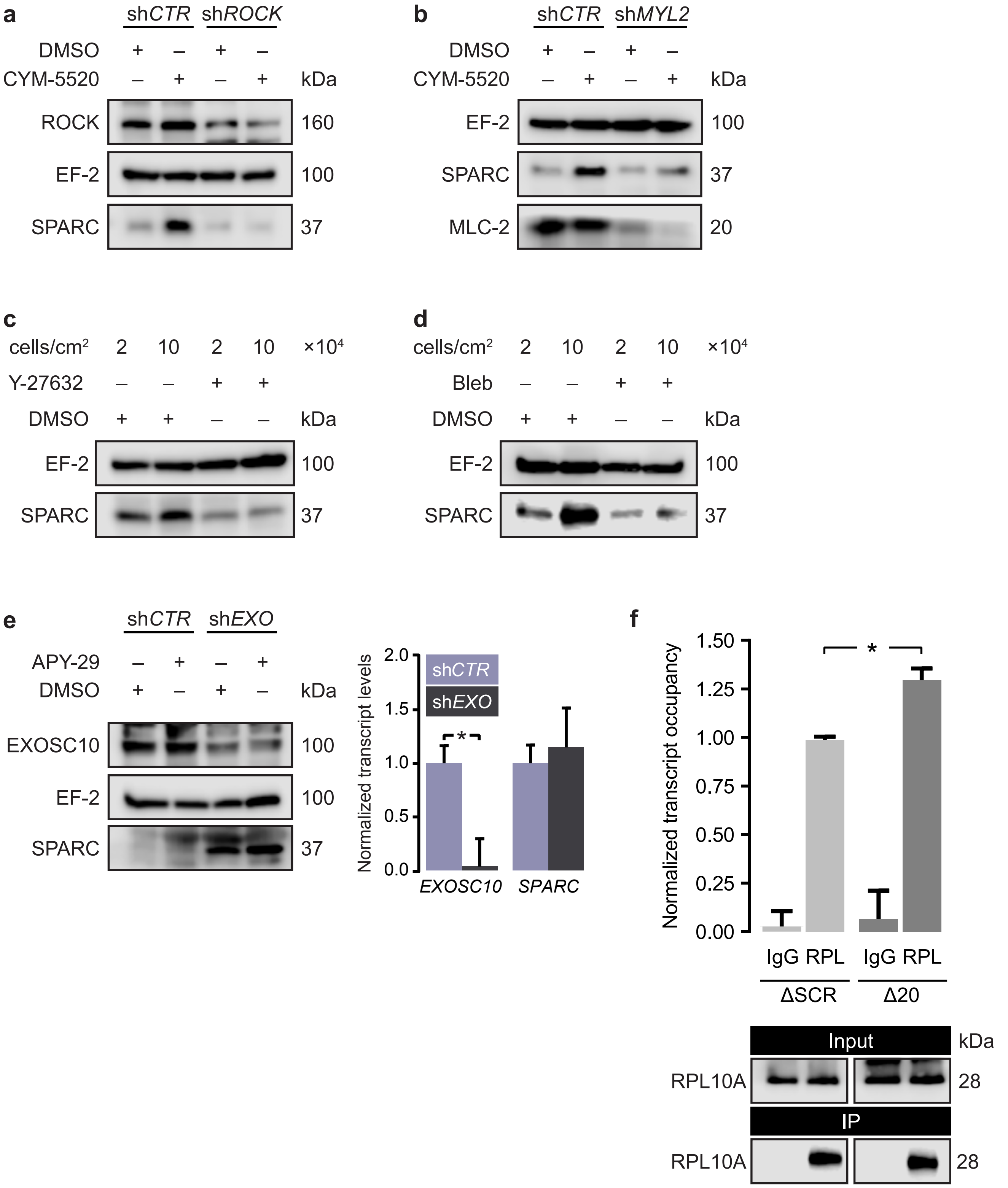

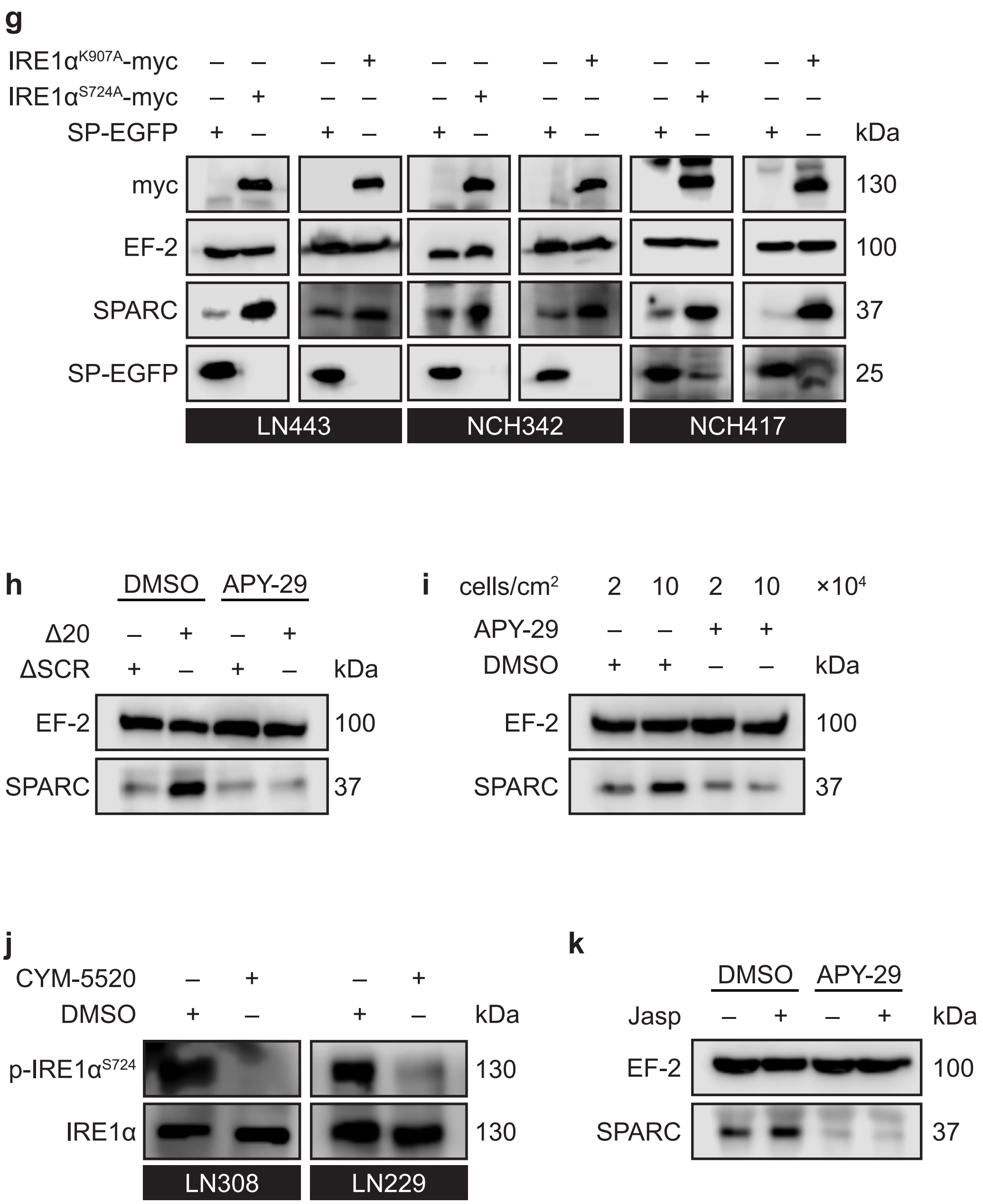
g
IRE1α^K907A-myc
IRE1α^S724A-myc
SP-EGFP
kDa
myc
EF-2
SPARC
SP-EGFP
130
100
37
25
LN443
NCH342
NCH417
h
DMSO
APY-29
Δ20
ΔSCR
kDa
EF-2
SPARC
100
37
i
cells/cm2
2
10
2
10
×10^4
APY-29
DMSO
kDa
EF-2
SPARC
100
37
j
CYM-5520
DMSO
kDa
p-IRE1α^S724
IRE1α
130
130
LN308
LN229
k
DMSO
APY-29
Jasp
kDa
EF-2
SPARC
100
37

m

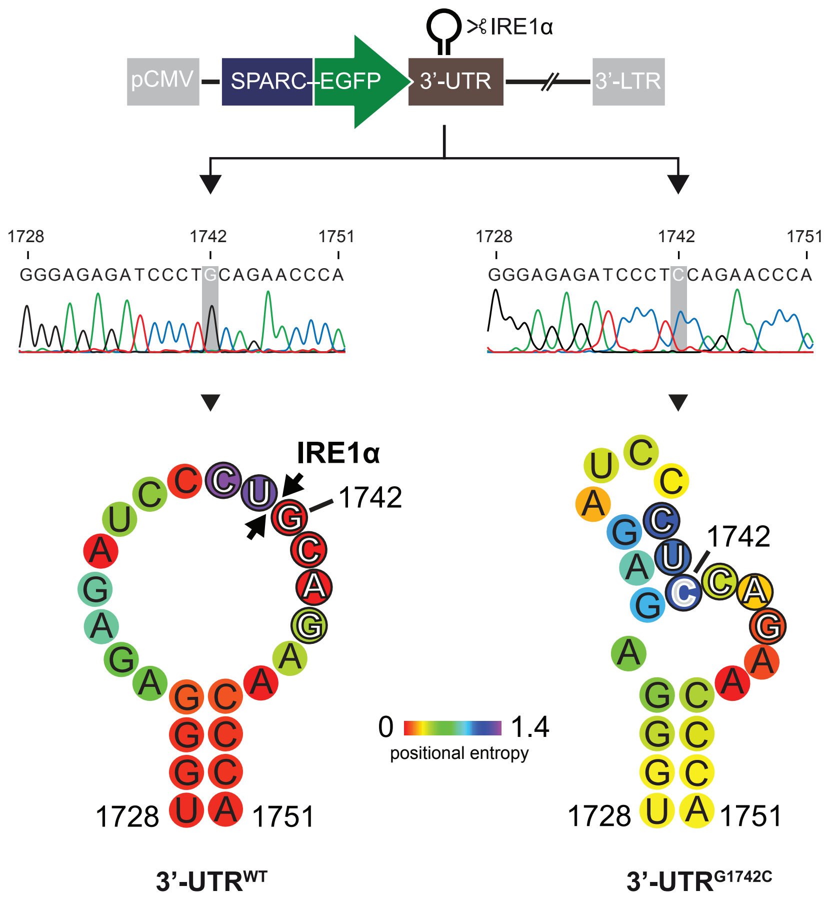

IRE1α
pCMV
SPARC-EGFP
3'-UTR
3'-LTR
1728
1742
1751
GGGAGAGATCCCTGCAGAACCCA
GGGAGAGATCCCTCCAGAACCCA
IRE1α
1742
1728
1751
0
1.4
positional entropy
3'-UTR^WT
3'-UTR^G1742C

n

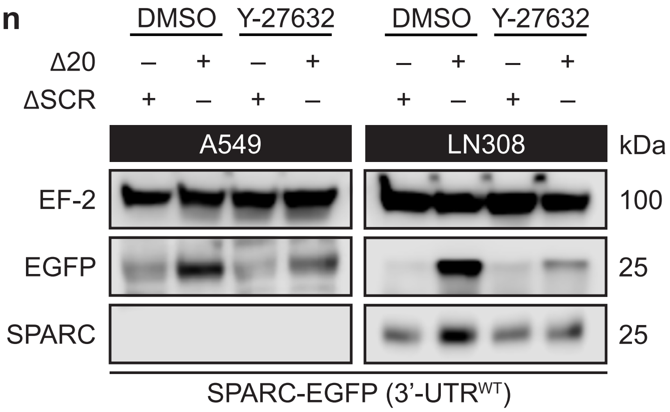

DMSO
Y-27632
DMSO
Y-27632
Δ20 – + – + – + – +
ΔSCR + – + – + – + –
A549
LN308
kDa
EF-2
100
EGFP
25
SPARC
25
SPARC-EGFP (3'-UTR^WT)

o

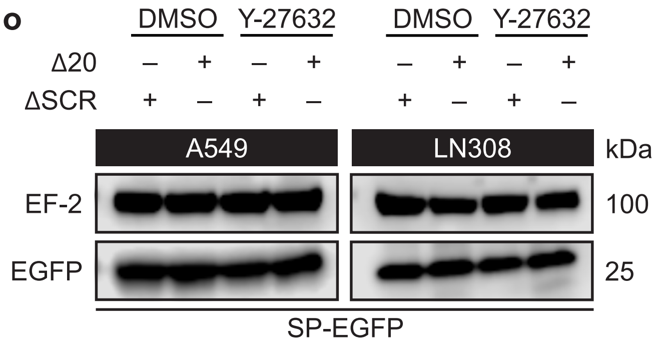

DMSO
Y-27632
DMSO
Y-27632
Δ20 – + – + – + – +
ΔSCR + – + – + – + –
A549
LN308
kDa
EF-2
100
EGFP
25
SP-EGFP

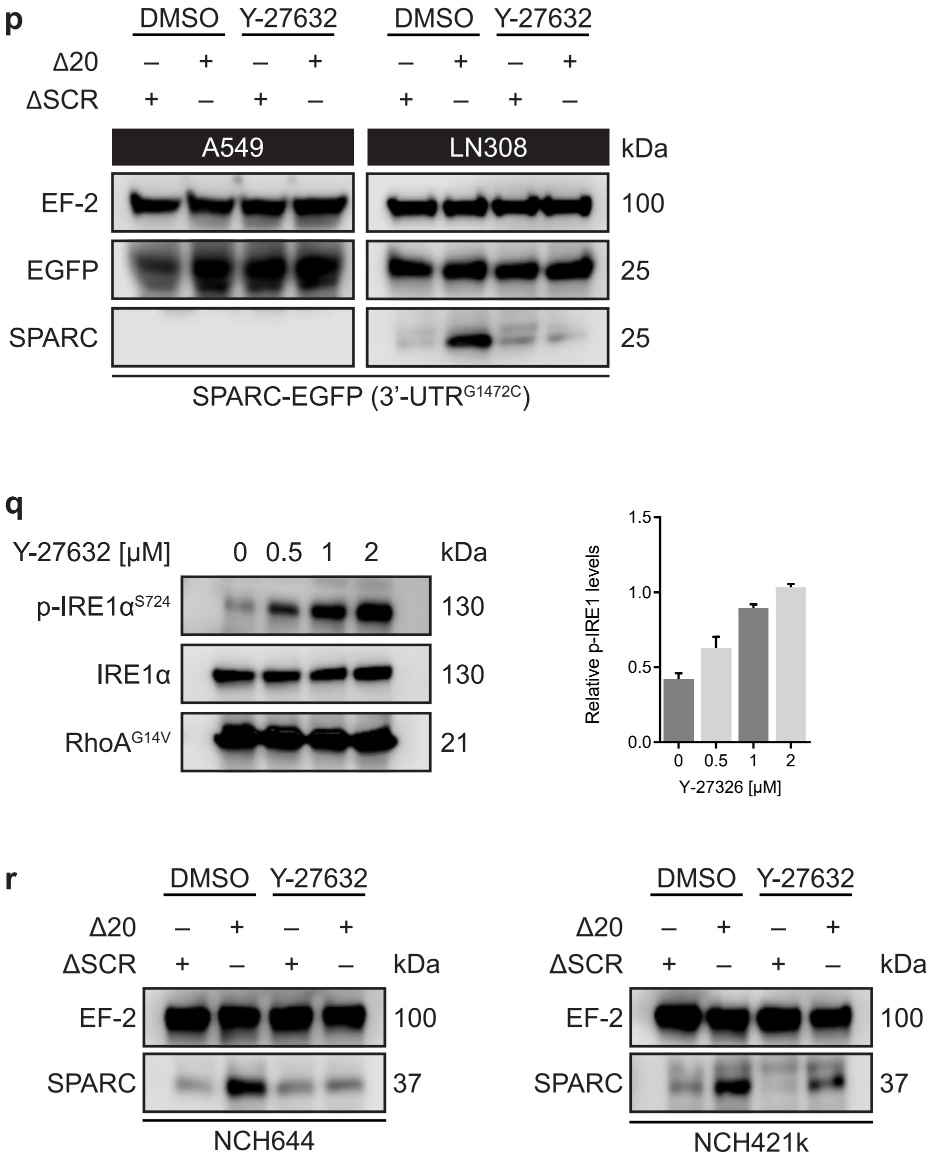

Figure S5. RhoA-induced deactivation of IRE1α initiates SPARC translation. Related to Figure 3.

(a, b) SPARC levels in LN308 cells treated with 5 µM CYM-5520. Control shRNA (sh*CTR*); shRNA against *ROCK* (sh*ROCK*); shRNA against *MYL2* (sh*MYL2*), which encodes MLC-2. (c, d) SPARC levels in (c) LN308 cells or (d) LN433 cells from low (2×10^4 cells) or high (10×10^4 cells) density cultures. Cells were treated for 16 h with either (c) 1 µM Y-27632 or (d) 1 µM Blebbistatin (Bleb). (e) LN308 glioma cell treated with APY-29 for 16 h. Gene expression analysis of *SPARC* and *EXOSC10* transcripts in LN308 cells. Error bars represent the SD, n = 3. Unpaired t-test, * $p \leq 0.05$; ** $p \leq 0.01$; *** $p \leq 0.001$; not significant = $p > 0.05$. Control shRNA (sh*CTR*) or shRNA against *EXOSC10* (sh*EXO*). (f) Polysomal occupancy of SPARC transcripts. Nogo-A-ΔSCR (ΔSCR); Nogo-A-Δ20 (Δ20). (g) SPARC levels in glioma cells expressing either IRE1α^{S724A}, IRE1α^{K907A} or EGFP fused to an N-terminal signal peptide (SP-EGFP); (h) LN308 glioma cells treated with 1 µM APY-29 for 16 h; (i) LN443 cells cultured at either low (2×10^4 cells) or high (10×10^4 cells) density. (j) Levels of p-IRE1α^{S724} in glioma cells treated with 5 µM CYM-5520 for 2 h. (k) SPARC levels in LN308 cells treated with 1 µM jasplakinolide (Jasp) for 16 h. (m) Mutation of the IRE1α recognition site present in the 3’-UTR of SPARC. (n, o, p) Cells were treated with 1 µM Y-27632 for 16 h. Nogo-A-ΔSCR (ΔSCR); Nogo-A-Δ20 (Δ20). (q) Phosphorylation levels of IRE1α expressing RhoA^{G14V} at increasing concentrations of Y-27632. Error bars represent the SD, n = 3. Unpaired t-test, * $p \leq 0.05$; ** $p \leq 0.01$; *** $p \leq 0.001$; not significant = $p > 0.05$. (r) SPARC levels in patient-derived glioma cells cultured under serum free conditions.
