# Supplementary material for: RhoA regulates translation of the Nogo-A decoy SPARC in white matter-invading glioblastomas
Source: Acta Neuropathol. 2019 May 6;138(2):275–93. doi: 10.1007/s00401-019-02021-z (PMC6660512; doi:10.1007/s00401-019-02021-z)
Supplement: Supplementary file 6 — Supplementary material 6 (PDF 1029 kb) [file 401_2019_2021_MOESM6_ESM.pdf]

**SUPPLEMENTAL FIGURE 6**

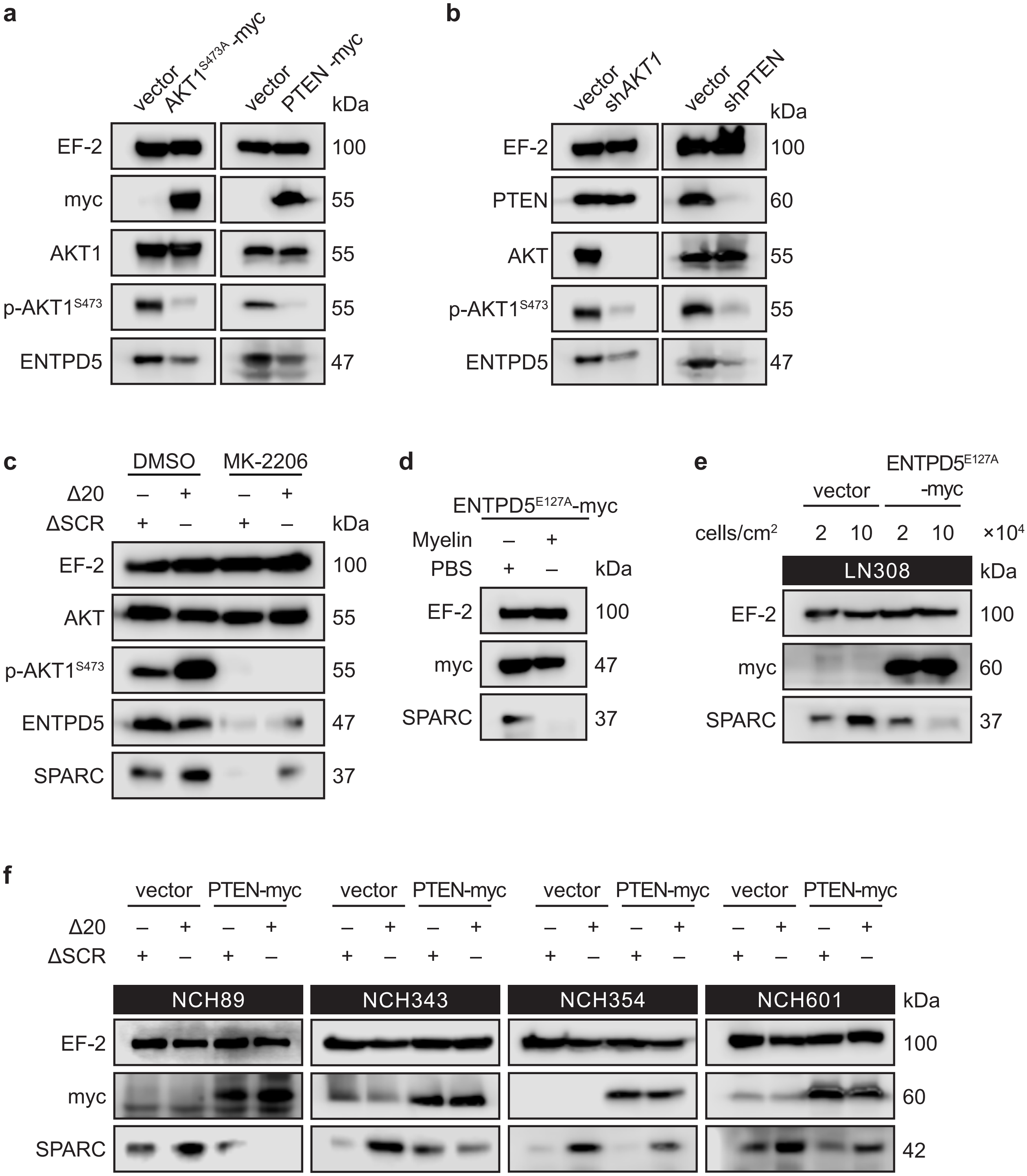

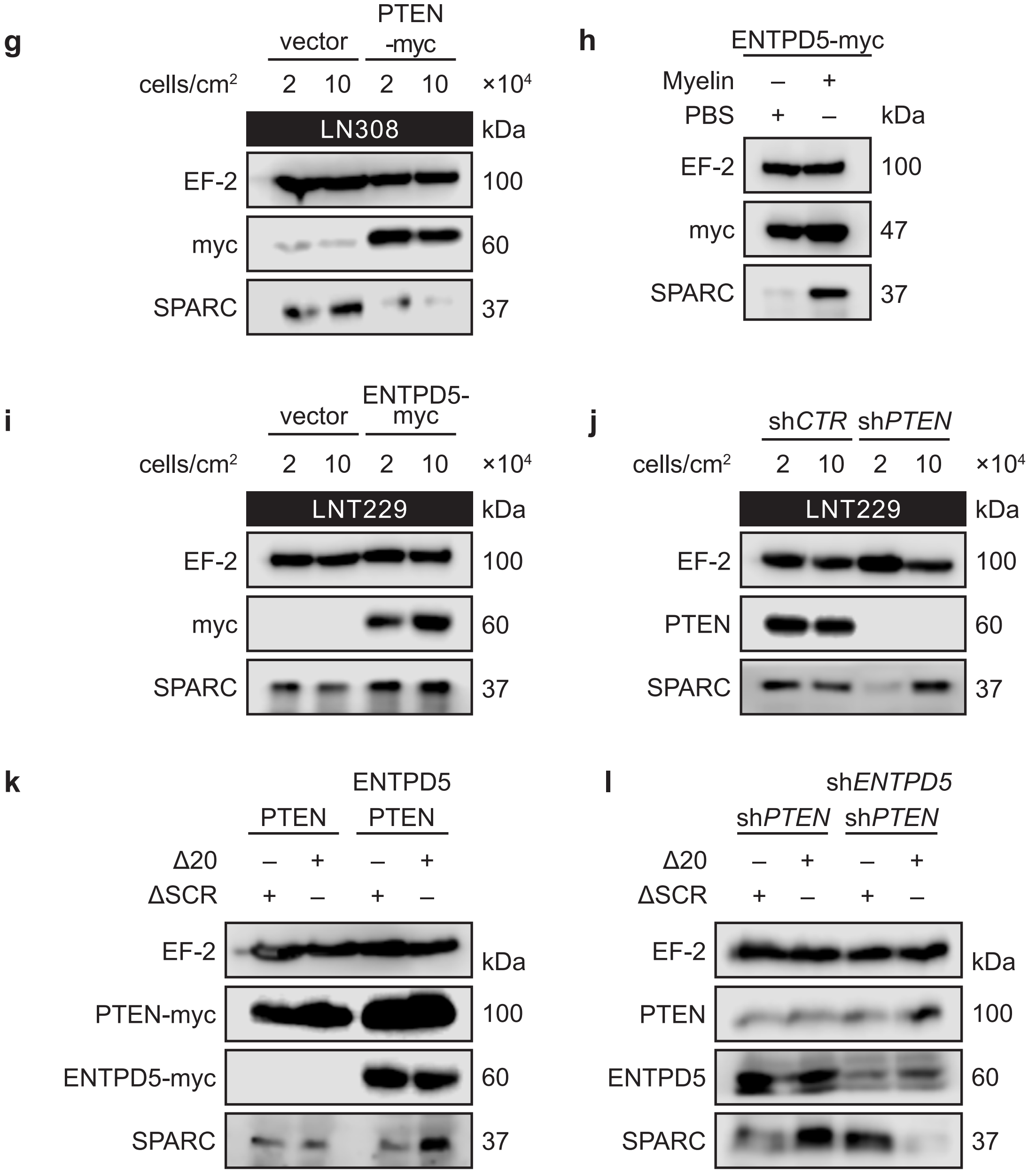


**Figure S6. Increased ENTPD5 expression due to high p-AKT levels allows SPARC production. Related to Figure 4.** (a, b) ENTPD5 levels in (a) LN443 cells expressing either myc-tagged AKT1 or PTEN or (b) LN18 glioma cells expressing control shRNA (sh*CTR*), shRNA against *AKT1* (sh*AKT1*) or shRNA against *PTEN* (sh*PTEN*). (c) ENTPD5 and SPARC levels in LN18 glioma cells treated with MK-2206 for 16 h. (d, e) SPARC levels in LN308 glioma cells expressing ENTPD5$^{E127A}$-myc. (f) SPARC levels in glioma cells expressing PTEN-myc. (g, h) SPARC levels in (g) LN308 cells expressing PTEN-myc. (h, i) LNT229 glioma cells expressing ENTPD5-myc. (j) LNT229 cells expressing sh*PTEN*. (k) LN308 glioma cells expresing PTEN-myc or PTEN-myc/ENTP5-myc. (l) LNT229 glioma cells expressing sh*PTEN* or sh*PTEN*/sh*ENTPD5*.
