# Supplementary material for: RhoA regulates translation of the Nogo-A decoy SPARC in white matter-invading glioblastomas
Source: Acta Neuropathol. 2019 May 6;138(2):275–93. doi: 10.1007/s00401-019-02021-z (PMC6660512; doi:10.1007/s00401-019-02021-z)
Supplement: Supplementary file 7 — Supplementary material 7 (PDF 528 kb) [file 401_2019_2021_MOESM7_ESM.pdf]

## SUPPLEMENTAL FIGURE 7

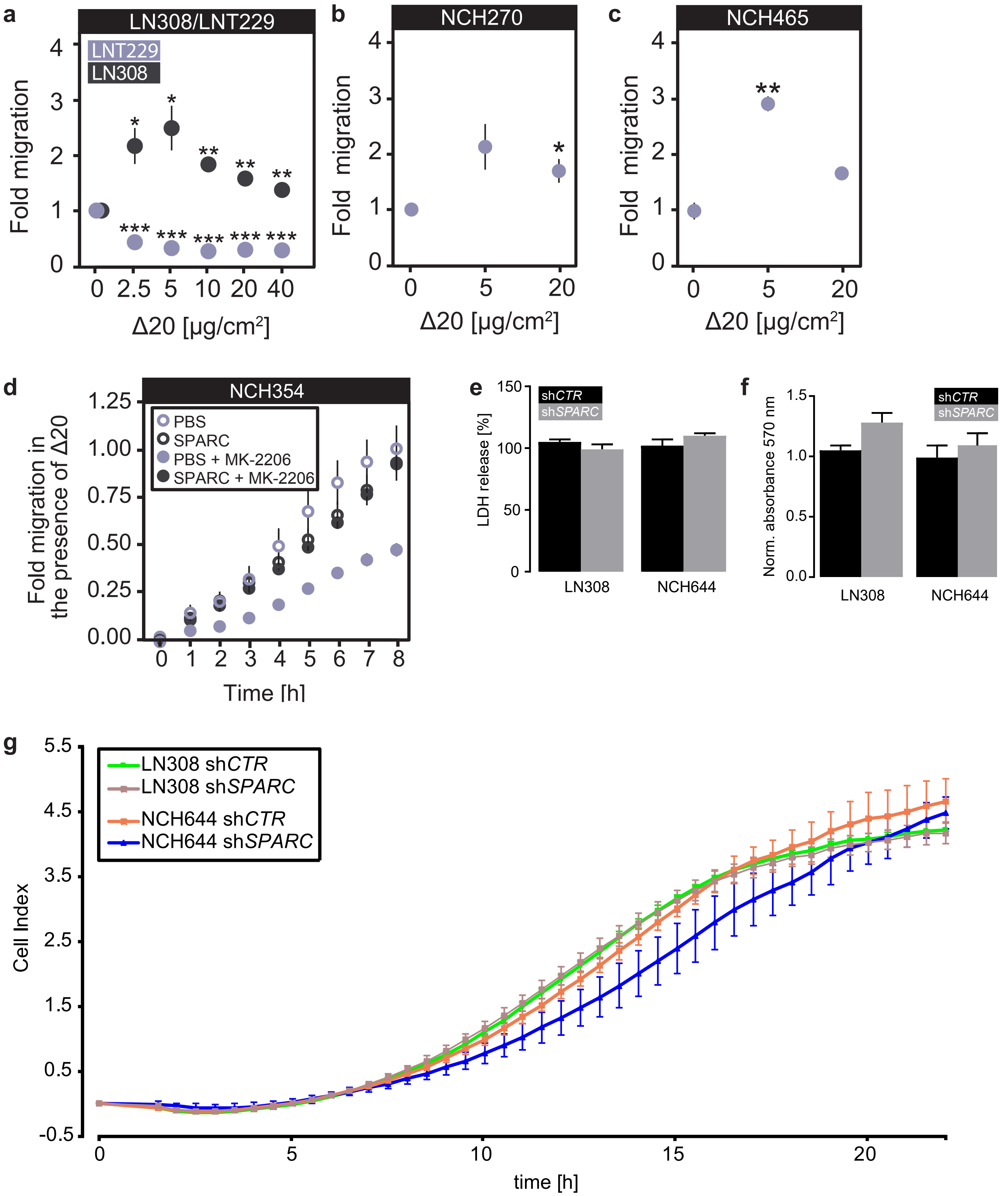


**Figure S7. Glioma cells require SPARC to migrate on myelinated structures *in vitro*. Related to Figure 5.**
(a, b, c) Invasion of glioblastoma cells in the presence of increasing Nogo-A-Δ20 (Δ20) concentrations. (d) Migration of NCH354 cells in the presence of equimolar amounts of SPARC and/or 1 μM MK-2206. (e) LDH-release assay, (f) MTT assay or (g) RTCA cell growth assay using glioblastoma cells expressing either sh*SPARC* or sh*CRT*. (a-f) Unpaired t-test, error bars represent the SD, * $p \leq 0.05$; ** $p \leq 0.01$; *** $p \leq 0.001$; ns. = $p > 0.05$; n = 3.
