# Supplementary material for: RhoA regulates translation of the Nogo-A decoy SPARC in white matter-invading glioblastomas
Source: Acta Neuropathol. 2019 May 6;138(2):275–93. doi: 10.1007/s00401-019-02021-z (PMC6660512; doi:10.1007/s00401-019-02021-z)
Supplement: Supplementary file 8 — Supplementary material 8 (PDF 3341 kb) [file 401_2019_2021_MOESM8_ESM.pdf]

SUPPLEMENTAL FIGURE 8

a

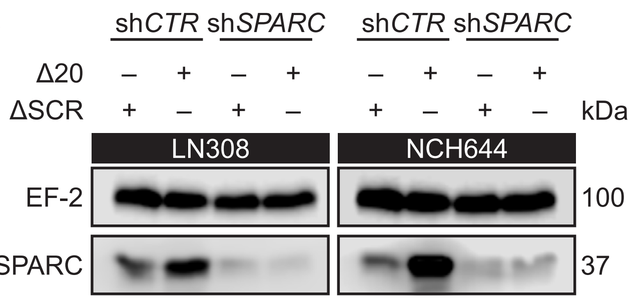

b

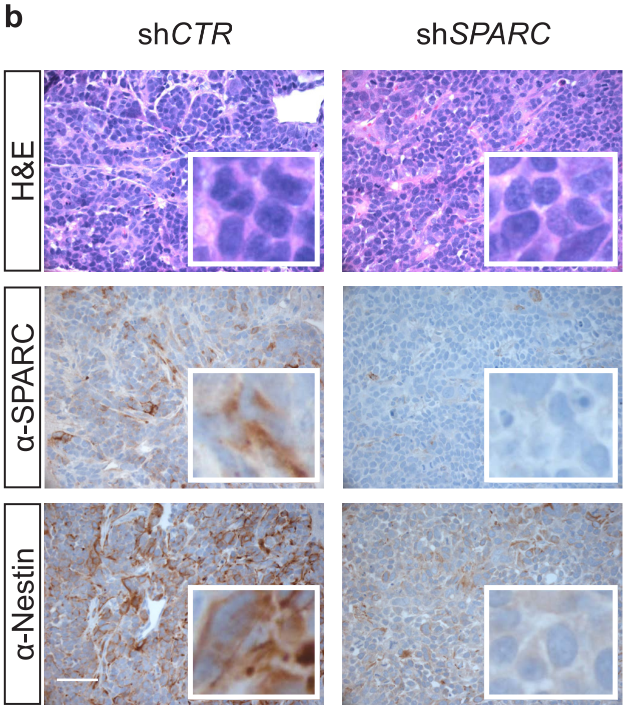

c

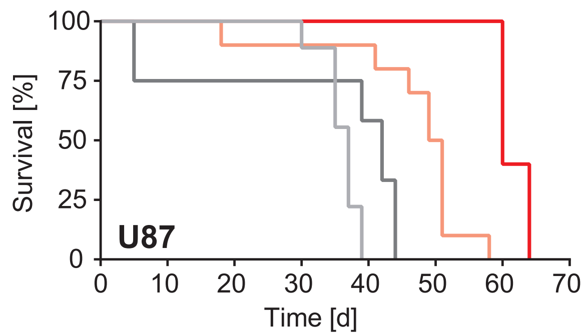

d

Group comparison			p value	
shCTR	vs.	shCTR + TMZ	0.0695	ns.
shCTR	vs.	shSPARC	0.0300	*
shCTR	vs.	shSPARC + TMZ	< 0.001	***
shCTR + TMZ	vs.	shSPARC + TMZ	< 0.001	***
shSPARC	vs.	shSPARC + TMZ	< 0.001	***

e

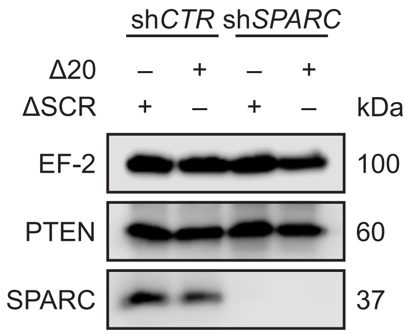

f

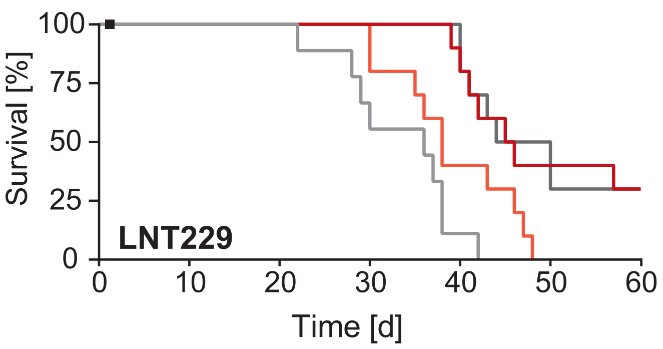

g

Group comparison			p value	
shCTR	vs.	shCTR + TMZ	0.0034	**
shCTR	vs.	shSPARC	0.2170	ns.
shCTR	vs.	shSPARC + TMZ	0.0016	**
shCTR + TMZ	vs.	shSPARC + TMZ	0.9980	ns.
shSPARC	vs.	shSPARC + TMZ	0.0139	*

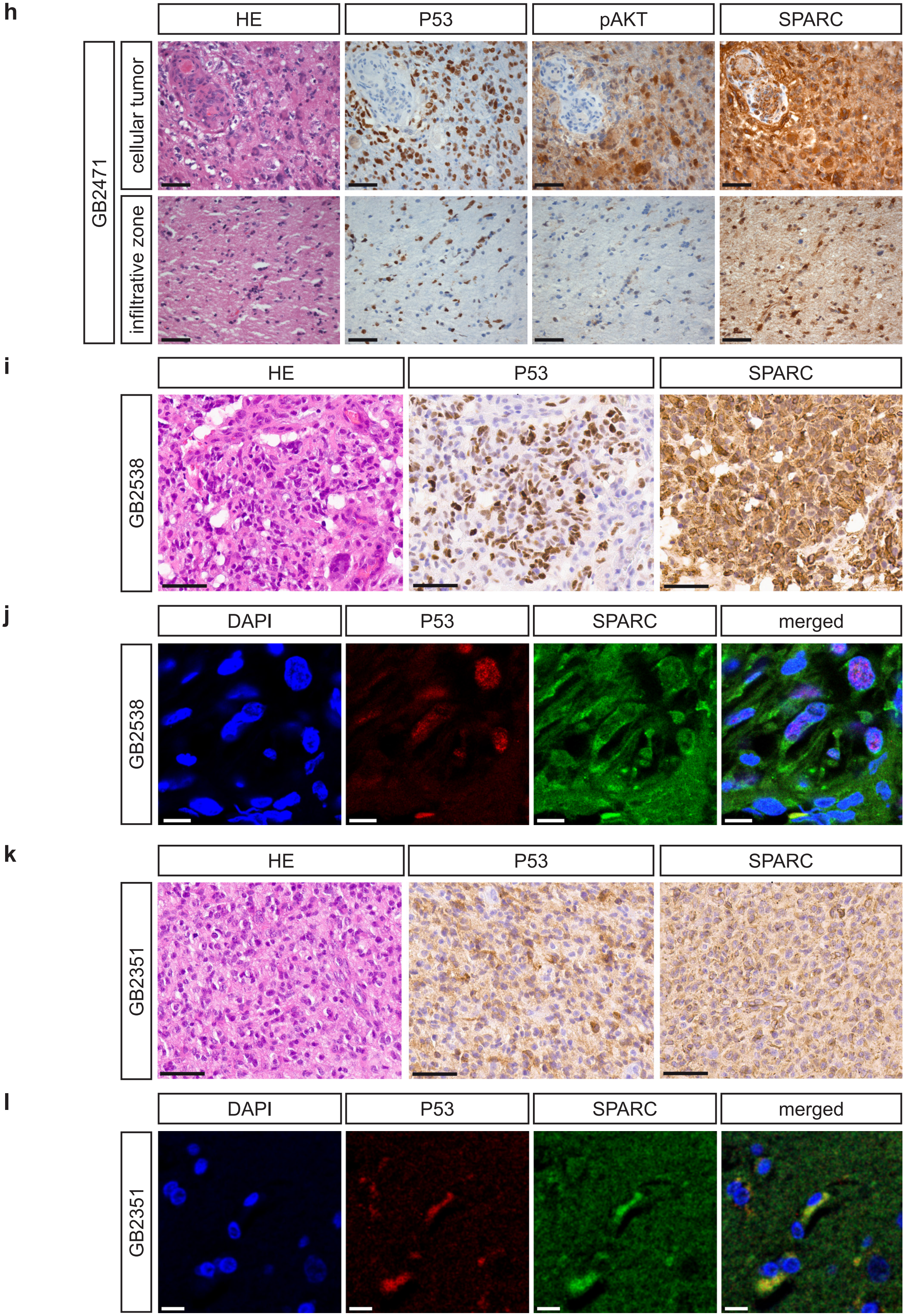
h
HE
P53
pAKT
SPARC
GB2471
cellular tumor
infiltrative zone
i
HE
P53
SPARC
GB2538
j
DAPI
P53
SPARC
merged
GB2538
k
HE
P53
SPARC
GB2351
l
DAPI
P53
SPARC
merged
GB2351

Figure S8. Glioma cells require SPARC for infiltrative growth. Related to Figure 7.
(a, b) SPARC levels in (a) glioma cells in the presence of Nogo-A-Δ20 and (b) tumors generated from xenografted LN308$^{EGFP-2A-FLuc}$ cells. Scale bar: 50 µM. (c, d, f, g) Kaplan-Meier analysis of NSG mice xenografted with (c, d) U87$^{EGFP-2A-FLuc}$ or (f, g) LNT229$^{EGFP-2A-FLuc}$ cells. Mice that died due to a non-tumor-related cause were censored by a symbol: sh*CTR* (circle), sh*CTR* + TMZ (square), sh*SPARC* (triangle), sh*SPARC* + TMZ (rhombus). (e) Knock-down of SPARC in LNT-229 cells. (h) Histology (hematoxylin-eosin, HE) as well as immunohistochemical stainings for p53, pAKT and SPARC in consecutive sections of a selected IDH-wildtype glioblastoma that carries a p53 mutation leading to nuclear p53 accumulation in the tumor cells (GB2471). Shown are representative areas of cellular tumor tissue (upper panel) and infiltration zone (lower panel). Note that SPARC expression is strong in cellular areas with p53-positive tumor cells that also express pAkt (upper panel), but is also seen in invading p53-positive tumor cells, some of which additionally express pAKT (lower panel) (scale bars: 50 um). (i) Histology (HE) as well as immunohistochemical stainings for p53 and SPARC in another IDH-wildtype glioblastoma with p53 mutation and nuclear p53 accumulation in the tumor cells (GB2538). Again, the area with many p53-positive tumor cells shows widespread expression of SPARC (scale bars: 50 um). (j) Confocal immunofluorescent images of GB2538 demonstrating p53-positive tumor cells with SPARC positivity. Shown is a selected tumor area stained for DAPI, p53 and SPARC (scale bars: 10 um). (k) Histology (HE) as well as immunohistochemical stainings for EGFRvIII and SPARC in cellular tumor tissue of a selected IDH-wildtype glioblastoma with EGFR gene amplification and EGFRvIII expresion (GB2351). Note that the depicted area with EGFRvIII-positive tumor cells also shows strong expression of SPARC (scale bars: 50 um). (l) Confocal immunofluorescent images from GB2351 confirming co-expression of EGFRvIII and SPARC in tumor cells. Shown is a selected tumor area stained for DAPI, EGFRvIII and SPARC (scale bars: 10 um).
