# Supplementary material for: RhoA regulates translation of the Nogo-A decoy SPARC in white matter-invading glioblastomas
Source: Acta Neuropathol. 2019 May 6;138(2):275–93. doi: 10.1007/s00401-019-02021-z (PMC6660512; doi:10.1007/s00401-019-02021-z)
Supplement: Supplementary file 11 — Supplementary material 11 (DOCX 86 kb) [file 401_2019_2021_MOESM11_ESM.docx]

**Title**

**RhoA regulates translation of the Nogo-A decoy SPARC in white matter-invading glioblastomas**

# Authors

Peter Wirthschaft^1, 2^, Julia Bode^1, 2^, Himanshu Soni^1, 2^, Fabio Dietrich^1, 2^, Thomas Krüwel^1, 2^, Bernd Fischer^3^, Christiane B. Knobbe-Thomsen^4^, Giulia Rossetti^6, 7, 8^, Andreas Hentschel^9^, Norman Mack^10^, Kai Schönig^11^, Michael O. Breckwoldt^12, 13^, André Schmandke^14, 15, 16^, Stefan Pusch^17, 18^, Jan Medenbach^19^, Martin Bendszus^12^, Martin E. Schwab^14, 15^, Andreas von Deimling^17, 18^, Marcel Kool^10^, Christel Herold-Mende^20^, Guido Reifenberger^4, 5^, Robert Ahrends^9^, Björn Tews^1, 2,  21^

# Affiliations

^1^Schaller Research Group at the University of Heidelberg and the German Cancer Research Center (DKFZ), Im Neuenheimer Feld 581, 69120 Heidelberg, Germany

^2^Molecular Mechanisms of Tumor Invasion (V077), DKFZ, Im Neuenheimer Feld 581, 69120 Heidelberg, Germany

^3^ Computational Genome Biology, German Cancer Research Center (DKFZ), Im Neuenheimer Feld 581, 69120 Heidelberg, Germany

^4^Department of Neuropathology, Heinrich Heine University Düsseldorf, Moorenstrasse 5, 40225 Düsseldorf, Germany

^5^German Cancer Consortium (DKTK), partner site Essen/Düsseldorf, 40225 Düsseldorf, Germany

^6^Computational Biomedicine, Institute for Advanced Simulation IAS-5 and Institute of Neuroscience and Medicine INM-9, Forschungszentrum Jülich, Wilhelm-Johnen-Straße, 52428, Jülich, Germany

^7^Jülich Supercomputing Centre (JSC), Forschungszentrum Jülich, Jülich, Germany, Wilhelm-Johnen-Straße, 52428, Jülich, Germany

^8^Department of Oncology, Hematology and Stem Cell Transplantation, RWTH Aachen University, Aachen, Pauwelsstraße 30, 52074 Aachen, Germany

^9^Leibniz-Institut für Analytische Wissenschaften - ISAS - e.V., Otto-Hahn-Str. 6b, 44227 Dortmund, Germany

^10^Division of Pediatric Neurooncology, DKFZ, Im Neuenheimer Feld 580, 69120 Heidelberg, Germany

^11^Central Institute of Mental Health, Medical Faculty Mannheim / Heidelberg University, Department of Molecular Biology, J 5, 68159 Mannheim, Germany

^12^Neuroradiology Department, University Hospital Heidelberg, Im Neuenheimer Feld 400, 69120 Heidelberg, Germany

^13^Clinical Cooperation Unit Neuroimmunology and Brain Tumor Immunology, DKFZ, Im Neuenheimer Feld 280, 69120 Heidelberg, Germany

^14^Brain Research Institute, University of Zurich, Winterthurerstrasse 190, 8057 Zurich, Switzerland

^15^Dept. of Health Sciences and Technology, ETH Zurich, Universitätstrasse 2, 8092 Zurich, Switzerland

^16^Present Address: Max Planck Institute for Demographic Research, Konrad-Zuse-Straße 1, 18057 Rostock, Germany

^17^Department of Neuropathology, University of Heidelberg, Im Neuenheimer Feld 224, 69120 Heidelberg, Germany

^18^Clinical Cooperation Unit Neuropathology, German Cancer Research Center (DKFZ) and DKTK, Im Neuenheimer Feld 224, 69120 Heidelberg, Germany

^19^Biochemistry I - Institute for Biochemistry, Genetics and Microbiology, University of Regensburg, Universitätsstraße 31, 93053 Regensburg, Germany

^20^Division of Experimental Neurosurgery, Department of Neurosurgery, Heidelberg University Hospital, Im Neuenheimer Feld 400, 69120 Heidelberg, Germany

^21^Present Address: Björn Tews, AbbVie Deutschland GmbH & Co.KG, Wiesbaden, Germany

**Inventory**

# Supplementary Figures

## Suppl. Figure 1

Glioma cells respond to Nogo-A by activating S1PR2 and its downstream effector RhoA. Related to Figure 1.

## Suppl. Figure 2

Glioma cells secrete the Nogo-A decoy SPARC upon RhoA activation. Related to Figure 2.

## Suppl. Figure 3

SPARC binds to Nogo-A. Related to Figure 2.

## Suppl. Figure 4

A minimal disordered region of Nogo-A-Δ20 is responsible for binding both S1PR2 and SPARC. Related to Figure 2.

## Suppl. Figure 5

RhoA-induced deactivation of IRE1α initiates SPARC translation. Related to Figure 3.

## Suppl. Figure 6

Increased ENTPD5 expression due to high p-AKT levels allows for SPARC production. Related to Figure 4.

## Suppl. Figure 7

Glioma cells require SPARC to migrate on myelinated structures in vitro. Related to Figure 5.

## Suppl. Figure 8

Glioma cells require SPARC for infiltrative growth. Related to Figure 7.

# Supplemental Movies

## Supplementary Movie 1

Glioma cells require SPARC to invade white matter in vivo. Related to Figure 6.

Wirthschaft_Supplementary_Movie_1.mpeg

## Supplementary Movie 2

Glioma cells require SPARC to invade white matter in vivo. Related to Figure 6.

Wirthschaft_Supplementary_Movie_2.mpeg

# Supplementary Methods

## Cell culture

All cells were cultured under humidified air conditions with 5% CO_2_ at 37 °C. All cells were grown in DMEM (Sigma Aldrich, Germany) containing 5% of fetal calf serum (Biochrome, Germany) except for CHO cells which were grown in chemically defined, serum-free PowerCHO medium (Lonza, Germany). Adhesion surfaces were coated with proteins for 24 h at 4 °C. Concentrations were at 30 pmol/cm^2^ for Nogo-A-Δ20, Nogo-A-ΔSCR and deletion mutants, at 10 µg/cm^2^ for myelin, at 30 pmol/cm^2^ for SPARC or SPARC(del_Kazal), at 30 pmol/cm^2^ for NEP1-40 (Sigma Aldrich, Germany) and at 50 pmol/cm2 for ECL-2, ECL-3 and ECL-SCR. All proteins and peptides were diluted in PBS (AppliChem, Germany). Peptides were synthesized by JPT Peptide Technologies (Germany). After infection with lentivirus, cells were selected in growth medium containing 2-7 µg/ml of puromycin (BioMol, Germany). To study density-induced phenotypes, equal numbers of cells were seeded on a surface area of either 9 cm^2^ or 152 cm^2^. All cell lines were continuously monitored for mycoplasma according to the recommendations of the German Collection of Microorganisms and Cells (Germany). All cell lines were authenticated by single nucleotide polymorphism profiling. Cell lines are listed in Table 2. Compounds for cell treatments are listed in Table S6.

Cytotoxicity was monitored using LDH-Glo (Promega, Germany) according to the manufacturer. Cell viability was assessed using the CellTiter 96 Non-Radioactive Cell Proliferation Assay (Promega, Germany) according to the manufacturer.

## Lentivirus production

Lentiviruses pseudotyped with the G-protein of Vesicular Stomatitis Virus was produced in HEK-293 cells (ATCC, USA) by co-transfecting pMD2.G (Addgene, USA), psPAX2 (Addgene, USA) and pLKO.1 (Addgene, USA) or pLVX (Clontech, USA) using polyethyleneimine (Sigma Aldrich, Germany). The virus-containing cell culture medium was centrifuged for 5 min at 600 × g and filtered through 0.45 µm cellulose acetate filters (Merck-Milipore, Germany). Virions were precipitated by centrifugation for 90 min at 45,000 × g and 4° C. Virions were resuspended in cold OptiMEM (Gibco, Germany) and stored at -80 °C. The virus titer was determined by infecting target cells using ten-fold dilution series of virions. Cells were infected and selected as described above. Cells were stained with hexamethyl pararosaniline chloride (Sigma Aldrich, Germany) and counted. A tissue culture infection dose required to infect 50% of the cell population (TCID_50_) was used for further experiments. Plasmids are listed in Table 6.

## Myelin extractions

Brain tissue was homogenized in extraction buffer (20 mM Tris (Roth, Germany), 60 mM CHAPS (Sigma Aldrich, Germany), 1 mM EDTA (Roth, Germany), 1x Complete Protease Inhibitor (Roche, Germany), 1x PhosSTOP (Roche, Germany), pH 8.0), lyzed on ice for 30 min and centrifuged for 30 min at 24,000 x g, 4 °C. Protein concentration was determined using the Protein Assay Dye (BioRad, Germany) according to the manufacturer.

## Confocal laser scanning microscopy (CLSM)

**Sample preparation.** Cells were seeded on coated borosilicate glass coverslips (Roth, Germany), fixed with PBS (AppliChem, Germany) containing 4% of formaldehyde (Roth, Germany) for 20 min at room temperature, washed with PBS (AppliChem, Germany), and permeabilized with PBS (AppliChem, Germany) containing 0.1% of Triton-X100 (Sigma Aldrich, Germany). Cells were washed with PBS (AppliChem, Germany) and stained with primary antibody diluted in blocking buffer (PBS (AppliChem, Germany), 3% of fetal calf serum (Biochrome, Germany), 1% of bovine serum albumine (AppliChem, Germany)) overnight at 4 °C. Cells were washed with PBS (AppliChem, Germany) and incubated for 1 h with Alexa Fluor-coupled secondary antibodies (Invitrogen, Germany) or with Alexa Fluor-488-coupled phalloidin (Invitrogen, Germany) diluted in blocking buffer. Cell nuclei were stained with 4',6-diamidino-2-phenylindole (Sigma Aldrich, Germany). Cover slips were mounted on glass slides (Roth, Germany) using VectaShield (Vecta Technologies, Germany).

**Measurement**. Fluorescence was detected using an LSM 710 microscope (Zeiss, Germany), recorded using ZEN 2012 software (Zeiss, Germany), and processed using ImageJ v1.47 (National Institutes of Health, USA). Antibodies are listed in Table S5.

## Ultramicroscopy (UM)

**Sample preparation**. Brains were optically cleared as previously described^1^. Briefly, brains were fixed in PBS (AppliChem, Germany) containing 4% of formaldehyde (Roth, Germany) after dissection and kept in PBS (AppliChem, Germany) at 4°C. Tissues were dehydrated with t-butanol (Sigma-Aldrich, Germany) diluted in HiPerSolv Chromanorm water (VWR Chemicals, France) at a range from 30% to 100%. Tissues were cleared with a 1:2 mixture of benzyl alcohol (Merck-Millipore, Germany) to benzyl benzoate (Sigma Aldrich, Germany). Dehydration solutions were changed every 24 h starting from the highest to the lowest dilution. Tissues were cleared for 48 h. After clearing, samples were stored at 4°C.

**Measurement**. Cleared whole brains were imaged using an UltraMicroscope II (LaVision BioTec, Germany) as described previously^2^. Images were taken at 1.26 × and 4.0 × magnification with a 2 × objective lens and a white light laser. For detection of GFP signals, a filter with an excitation range 470/24 nm and emission range 525/50 nm was used. Z-stacks with a 5 µm step size were acquired over a total range of 1500 to 2000 µm for whole brains. Z-stacks of whole brain were recorded at 351 pictures at 20 frames per second with an x/y-resolution of 720 pixels/576 pixels.

**Image segmentation.** Images were segmented in 2D separately for each z-position. Noise was reduced by linearly filtering the image with a Gaussian blur (s=3 pixels). Cells were segmented with an adaptive thresholding as implemented in the R/Bioconductor package EBImage and only cells that are located in the *corpus callosum* and not laying in image cracks were accepted. The *corpus callosum* was manually segmented at 8 z-positions and the region was linearly interpolated in z positions between. Regions with a fluorescence intensity 2 median deviations smaller than the median intensity of the corpus callosum were defined as tissue cracks. These crack regions were dilated with a disk shape of size 31 pixels. Further objects were only regarded as cells, if the ratio of maximum radius to minimum radius of the cell region as computed by EBImage is not exceeding 4. The 2D segments were combined to a 3D object with a greedy algorithm by scanning all 2D segments in the image stack in the order of their maximum intensity starting with the highest intensity. For one image segment all segments in all other z-positions that overlap at least 50% were assigned to the same 3D object. A 3D object is accepted as a cell object, if it is detected in at least three z-planes.

## Magnetic resonance imaging (MRI)

MRI was performed on a 9.4 Tesla horizontal bore small animal MRI scanner (BioSpec 94/20 USR, Bruker BioSpin GmbH, Germany) with a 4 channel phased array surface receiver coil. We used a 3D rare T1-w 3D post- Gadodiamide -contrast fast low angle shot (FLASH) sequence to delineate intracranial tumors (T1-w parameters: 3D sequence, TE: 1.9 ms, TR: 5 ms, Flip angle: 60°, acquisition matrix: 128 x 128, number of averages: 4, in plane resolution: 156 µm, duration: 05 min 28 s). 0.2 mmol/kg Gadodiamide (Nycomed, Germany) was administered as a contrast reagent to assess blood brain barrier disruptions caused by the tumor. MRI was performed at day 9 after tumor implantation. For MRI, animals were anesthetized with 2% isoflurane. Anesthesia was maintained with 0.5-1.5% isoflurane. Animals were kept on a heating pad to keep the body temperature constant. Animal respiration was monitored during imaging with a breathing surface pad controlled by an in house developed LabView program (National Instruments Corporation, Germany). MR images were visualized in OsiriX Imaging software (version 4.12 Pixmeo, Switzerland).

## Bioluminescence imaging

All mice were imaged at day 14, 17, 19 and 21 post tumor cell injection. D-luciferin was injected at a concentration of 150 mg/ kg body weight (Biomol, Germany). Mice were anaesthetized using 1.5 % of isoflurane (Abbott, Germany). After 10 min incubation, images were acquired using an IVIS Lumina II system (Caliper Life Science, USA) with exposure times of 1, 3 and 5 min. Bioluminescence signals were quantified with LivingImage 4.4 (PerkinElmer, USA).

## Immunohistochemistry (IHC)

For paraffin analyses, mouse brains were fixed in PBS (AppliChem, Germany) containing 4% of formaldehyde and cut into 2 mm coronal sections. After automated dehydration, sections were embedded in paraffin (Sigma Aldrich, Germany). 5 µm sections were cut and mounted onto StarFrost adhesive slides (Knittel, Germany). For further staining procedures, sections were de-paraffinized in xylene (Sigma Aldrich, Germany) followed by a descending ethanol (Sigma Aldrich, Germany) series and finally water. Standard protocols were used for hematoxylin/eosin (Sigma Aldrich, Germany) staining, for luxol fast blue-periodic acid Schiff (LFB-PAS; Sigma Aldrich, Germany) staining and immunohistochemical staining. Antigens were retrieved using antigen retrieval solution (Dako, Germany) except for staining of GFAP for which antigen was retrieved for 20 min using 1% of pepsin (Sigma Aldrich, Germany) in 10 mM HCl (Sigma Aldrich, Germany). Antibodies were detected using Vectastain Elite ABC Kit (VectorLab) and diaminobenzidine (Dako, Germany). Paraffin sections from human specimens were processed accordingly. Antibodies are listed in Table 5.

## Quantitative reverse transcription polymerase chain reaction (qRT-PCR)

Total RNA was isolated from cells using the NucleoSpin RNA II Kit (Macherey & Nagel, Germany) and reverse transcribed using the First Strand cDNA Synthesis Kit (Thermo Scientific, Germany). Transcripts were quantified on a LightCycler 480 (Roche, Germany) using the FastStart SYBR Green Master Kit (Roche, Germany). Fluorescence was recorded and analyzed using the LightCycler Gene Scanning Software 1.5 (Roche, Germany). Expression of target genes was normalized to the transcript levels of *EEF2*, *PGK1* and *PPIA* according to a previously established mathematical model^3^. Primers are listed in Table 3.

## Ribosomal profiling

Polysomal transcript occupancy was analyzed by precipitating ribosomes via the ribosomal subunit RPL10A in the presence of 100 mg/mL Cycloheximide (Sigma, Germany), followed by mRNA isolation as previously described. Transcript were quantified as described for gene expression analysis.

## Plasmid construction

DNA was amplified from plasmids or cDNA using Q5 High-Fidelity DNA Polymerase (NEB). DNA was digested using restriction endonucleases (NEB, Germany) and purified using the NucleoSpin Gel & PCR Clean-up Kit (Macherey & Nagel, Germany). DNA was dephosphorylated using antartic phosphatase (NEB, Germany) and ligated using T4 DNA ligase (Thermo Scientific, Germany). Plasmids were cloned in DH5α (NEB, Germany) and purified with either NucleoSpin Plasmid Mini Kit or NucleoBond PC500 Kit (Macherey & Nagel, Germany). Point mutations were introduced using the QuickChange II XL Site-Directed Mutagenesis Kit (Agilent Technologies). Plasmids were sequenced by MWG Eurofins (Germany). Plasmids are listed in Table 6.

## Recombinant protein production

**Production**. Nogo-A-Δ20, Nogo-A-Δ20N-RFP, Nogo-A-Δ20C-RFP, Nogo-A-Δ20min-RFP, Nogo-A-ΔSCR, ECL-2-EGFP, ECL-3-EGFP, and PGDFB were produced as His-tagged proteins in BL21 bacteria (Novagen, Germany). TrxA-SPARC and TrxA-SPARC(del_Kazal) His-tagged fusion proteins lacking the SPARC signal peptide were produced in SHuffle bacteria (NEB, Germany). Full-length Nogo-A and Nogo-B were produced as His-tagged proteins in suspension cultures of CHO cells (provided by C Rösli, DKFZ, Germany). EGFP-tagged SPARC and its deletion mutants as well as ECL-2-EGFP and ECL-3-EGFP without His-tag were produced in HEK293 cells (ATCC, USA). Bacteria were grown in 2YT medium (Roth, Germany) at 30 °C until an OD_600_ of 0.6-0.8. Protein expression was induced using 1 M of IPTG (Roth, Germany) and expressed for 4 h at 30 °C. CHO cells were grown in suspension as described above. Plasmids used for expression are listed in Table S5.

**Purification**. Bacteria or CHO cells were resuspended in lysis buffer (5 ml/mg BugBuster (Novagen, Germany), Lysozyme, Benzonase) and lyzed for 30 min at room temperature. Lysates were centrifuged for 15 min at 7,000 × g and sequentially filtered through 0.45 µm and 0.20 µm cellulose acetate filters (Merck-Milipore, Germany). Proteins were purified with HisTrap FF columns (GE Healthcare, Germany) according to the manufacturer. Eluates were dialyzed in Slide-a-Lyzer cassettes with a 10 kDa cut-off (Thermo Fisher, Germany) against 4 L of filtered PBS (AppliChem, Germany) overnight at 4 °C.

## Immunoaffinity chromatography

**Sample preparation.** Cells or tissue were lyzed in lysis buffer (25 mM Tris (Roth, Germany), 150 mM NaCl (Roth, Germany), 1 mM EDTA (Roth, Germany), 1% NP-40 (Sigma Aldrich, Germany), 5% glycerol (Roth, Germany), 1× Complete Protease Inhibitor (Roche, Germany), 1× PhosSTOP (Roche, Germany), pH 7.4) on ice for 30 min and centrifuged at 16,100 × g, 4 °C for 15 min. Protein concentration was determined using the Protein Assay Dye (BioRad, Germany) according to the manufacturer.

**Purification.** Immune complexes were formed by mixing 1-2 mg of protein from cleared lysates with 1-2 µg of antibody. After overnight incubation at 4 °C, immune complexes were purified with protein G-conjugated agarose resin (Roche, Germany). Alternatively, antibodies were cross-linked to protein G-conjugated agarose using 5 mM of suberic acid bis(3-sulfo-N-hydroxysuccinimide ester) (Thermo Scientific, Germany) according to the manufacturer. The resin was washed with cold washing buffer (12.5 mM Tris (Roth, Germany), 75 mM NaCl (Roth, Germany), 0.5 mM EDTA (Roth, Germany), 0.5% NP-40 (Sigma Aldrich, Germany), 2.5% glycerol (Roth, Germany), and pH 7.4). Proteins were eluted at pH 2.8 with IgG elution buffer (Thermo Scientific, Germany). Eluates were adjusted to neutral pH using 1 M Tris, pH 8. For immunoblotting, 4% of input was used.

## Immobilized-metal affinity chromatography (IMAC)

**Sample preparation.** Cells were lyzed using Pierce Lysis Buffer (Thermo Scientific, Germany; supplemented with 1× Complete Protease Inhibitor (Roche, Germany)) on ice for 30 min and centrifuged at 16,100 × g, 4 °C for 15 min. Protein concentration was determined using the Protein Assay Dye (BioRad, Germany) according to the manufacturer.

**Purification.** Protein complexes were purified overnight at 4 °C using a cobalt-charged nitrilotriacetic acid-conjugated agarose resin (Clontech, USA). The resin was washed thrice using Pierce Lysis Buffer (Thermo Scientific, Germany) supplemented with 10 mM imidazole. Proteins were eluted using Pierce Lysis Buffer (Thermo Scientific, Germany) supplemented with 300 mM of imidazole. Eluates were dialyzed against PBS (AppliChem, Germany) using Amicon Ultra-0.5 filters with a 10 kDa cutoff (Merck-Millipore, Germany). For immunoblotting, 4% of input was used.

## Glutathione affinity chromatography

GTP-bound RHOA was purified from cell lysates via GST-tagged Rhotekin coupled to glutathione-conjugated agarose resin using the RHOA Pull-down Activation Assay Biochem Kit (Cytoskleteon, USA). GTP-bound RHOA was purified from cells lysates via GST-tagged p21 binding domain of PAK coupled to glutathione-conjugated agarose resin using the Rac1 Pull-down Activation Assay Biochem Kit (Cytoskeleton, USA). For immunoblotting, 4% of input was used.

## Lectin affinity chromatography (LAC)

**Sample preparation.** LN308 cells expressing either EGFP or RHOA^G14V^ were cultured in serum-free medium for 48 h. Conditioned medium was concentrated and dialyzed against equilibration buffer (20 mM Tris (Roth, Germany), 0.5 M NaCl (Roth, Germany), 1 mM MnCl_2_ (Sigma Aldrich, Germany), 1 mM CaCl_2_ (Roth, Germany), 1× Complete Protease Inhibitor (Roche, Germany), pH 7.4) using Amicon Ultra-15 centrifugal filters with a 10 kDa cutoff (Merck-Millipore, Germany). Samples were adjusted according to the cell number counted at harvest.

**Purification.** Concanavalin A-conjugated agarose resin (Sigma Aldrich, Germany) was washed with pre-wash buffer (20 mM Tris (Roth, Germany), 0.5 M NaCl (Roth, Germany), 1 mM MnCl_2_ (Sigma Aldrich, Germany), 1 mM CaCl_2_ (Roth, Germany), pH 7.4) and incubated with the dialysate overnight at 4 °C. The resin was washed with equilibration buffer and glycoproteins were eluted in elution buffer (20 mM Tris (Roth, Germany), 500 mM methyl-alpha-D-glucopyranoside (Sigma Aldrich, Germany), pH 7.4). Eluates were dialyzed against PBS (AppliChem, Germany) using Amicon Ultra-0.5 filters with a 10 kDa cutoff (Merck-Millipore, Germany). For immunoblotting, 4% of input was used.

## Mass spectrometry

**Sample preparation.** The concentration of proteins isolated by lectin affinity chromatography was determined with Pierce BCA protein assay Kit (Thermo Scientific, Germany). Samples were reduced using 10 mM of 1,4-dithiothreitol (Roche, Germany) for 30 min at 56 °C and alkylated using 30 mM of iodoacetamide (Sigma Aldrich, Germany) for 30 min. Samples were further processed by filter aided sample preparation as previously described^4^ with the following modifications: samples were diluted to 10 mM of SDS (Roth, Germany) using 8 M of urea (Sigma Aldrich, Germany) in 100 mM of Tris-HCl (ApplicChem, Germany), pH 8.5) and centrifuged through spin filters (VWR International, Germany) for 25 min at 13,500 × g. Spin filters were washed three times using 8 M of urea in 100 mM of Tris-HCl (ApplicChem, Germany), pH 8.5. Samples were then washed three times with 50 mM of triethylammonium bicarbonate (Sigma Aldrich, Germany). Samples were digested with trypsin (Promega, Germany) at a 1:20 ratio of trypsin to protein in reaction buffer (50 mM TEAB, 0.2 M guanidinium chloride (Sigma Aldrich, Germany), 2 M CaCl_2_ (Sigma Aldrich, Germany)) for 12 h at 37 °C. Peptides were eluted with 50 mM triethylammonium bicarbonate (Sigma Aldrich, Germany) in 1% of trifluoroacetic acid (Biosolve, Netherlands). Digestion efficiency was determined using monolithic reverse-phase separation as previously described^5^.

**Nano-LC-MS/MS**. Samples were separated using an Ultimate 3000 Rapid Separation Liquid Chromatography system (Dionex, Netherlands) coupled to a QExactive Plus system (Thermo Scientific, Germany) for peptide analysis, using data dependent MS/MS acquisition. Peptides were concentrated using Acclaim C18 PepMap100 columns (100 µm x 2 cm; Thermo Scientific, Germany) followed by separation using Acclaim C18 PepMap100 columns (75 µm x 50 cm; Thermo Scientific, Germany) along a binary gradient (solvent A: 0.1% formic acid (Biosolve, Netherlands); solvent B: 0.1% formic acid in 84% acetonitrile (Biosolve, Netherlands)) at a flow rate of 250 nL/min. The linear gradient increased from 5% to 45% of solvent B over 200 min. Full MS scans were acquired at a resolution of 70,000 full width at half maximum (FWHM), followed by MS/MS with a resolution of 17,500 FWHM of the 15 most abundant ions. For the full scan, the automatic voltage gain was at 3 × 10^6^ ions and the maximum injection time was 120 ms. For MS/MS scans the automatic voltage gain was at 10^4^ ions and the maximum injection time was 250 ms. Precursors with charge states between +2 and +5 were selected for fragmentation. The precursor were isolated at 0.70 u (FWHM), emitter voltage was set to 1,500 V and the temperature of the transfer capillary to 250°C.

**Label free data analysis.** Label free quantification was performed using the Progenesis LC-MS 4.1 (Nonlinear Dynamics, United Kingdom). MS raw data were aligned in automatic mode. The exported peak lists were searched using the search engines X!Tandem PILEDRIVER (2015.04.01; Global Proteome Machine, Canada) via searchGUI 2.0.2 (ISAS, Germany) and Mascot 2.4 (Matrix Science, United Kingdom), each using the concatenated target/decoy version of the human Uniprot database (UniProt Consortium, United Kingdom). PeptideShaker 1.0.1 (Compomics, Ghent University, Belgium) was used for combination and data analysis to maximize the number of identified peptides and proteins. Search parameters were as follows: trypsin as protease with a maximum of two missed cleavages; carbamido-methylation of cysteine as fixed and oxidation of Met as variable modification. MS and MS/MS tolerances were at 10 ppm and 0.02 Da. Combining and filtering the search results was at a false discovery rate of 1% on the protein level prior to export and re-import into Progenesis LC-MS 4.1 (Nonlinear Dynamics, United Kingdom). Peptide sequences containing oxidized methionine and pyro-glutamine (derived from X!Tandem 2^nd^ pass search) were omitted from further data analysis. For each protein, the average of the normalized abundances (obtained from three replicates processed with Progenesis) was calculated to determine the ratios between experiment and control. Only proteins which were commonly quantified in all six samples with unique peptides, an ANOVA p-value of < 0.05 and with an average ratio of < 0.5 or > 2 were considered as regulated.

## Immunoblotting

**Sample preparation.** For total protein isolation, cells were incubated in lysis buffer (50 mM Tris (Roth, Germany), 1% Triton-X (Sigma Aldrich, Germany), 100 mM NaCl, 1 mM DTT (Roth, Germany), 1 mM sodium orthovanadat (AppliChem, Germany), 50 mM sodium fluoride (Sigma Aldrich, Germany), 10 mM β-glycerophosphate (Sigma Aldrich, Germany), 5% glycerol (Roth, Germany), 1× Complete Protease Inhibitor (Roche, Germany), 1× PhosSTOP (Roche, Germany) , pH 7.5) on ice for 30 min and centrifuged at 16,100 × g, 4 °C for 15 min. Protein concentration was determined using the Protein Assay Dye (BioRad, Germany) according to the manufacturer.

**Transfer and Detection.** Proteins were boiled in sample buffer for 5 min at 95° C, separated by discontinued SDS-PAGE, and transferred onto poly-vinyl difluoride membranes with 0.45 µm pore size (Merck-Millipore, Germany) at 2.5 A/25 V for 30 min. Membranes were incubated in blocking buffer (TBS (Applichem, Germany) with 1% of Tween-20 (Sigma Aldrich, Germany) and 5% of skimmed milk (AppliChem, Germany)) for 30 min. Proteins were detected by overnight incubation at 4 °C with primary antibodies diluted in TBS (Applichem, Germany) containing 1% of Tween-20 (Sigma Aldrich, Germany). Primary antibodies were detected by incubation for 1 h with horseradish peroxidase-coupled secondary antibody (Dianova, Germany) diluted in blocking buffer. After adding Pierce SuperSignal West substrate (Thermo Scientific, Germany), chemiluminescence was detected using a C-DiGit scanner (Licor, Germany), recorded using Image Studio 3.1.4 (Licor, Germany) and processed using ImageJ v1.47 (National Institutes of Health, USA). Antibodies are listed in Table 5.

## *In vitro* migration assays

Transwells (Corning, USA) were coated overnight at 4°C with myelin extract or Nogo-A-Δ20 diluted in PBS (AppliChem, Germany). Transwells were washed twice using PBS (AppliChem, Germany). Per well, 30,000 cells were seeded in serum-free medium. Bottom chambers were filled with 500 µl of growth medium. Transwells were incubated at 37 °C for 8 h. Cells were fixed with PBS (AppliChem, Germany) containing 4% of formaldehyde (Roth, Germany) overnight at 4 °C and stained with 4',6-diamidino-2-phenylindole (Sigma Aldrich, Germany). Cells were imaged using a Cell Observer microscope (Zeiss, Germany), recorded using ZEN 2012 software (Zeiss, Germany) and counted using ImageJ v1.47 (National Institutes of Health, USA).

## Real time cell analysis (RTCA)

Electronically-integrated transwells (Acea Biosciences, USA) were coated overnight at 4 °C with 5 µg/cm2 of Nogo-A-Δ20 diluted in PBS (AppliChem, Germany). The bottom chamber was filled with 190 µl of growth medium (DMEM (Sigma Aldrich, Germany) containing 10% of fetal calf serum (Biochrome, Germany)). The upper chamber was washed using PBS (AppliChem, Germany) and filled with 50 µl of DMEM (Sigma Aldrich, Germany). Transwells were incubated at 37°C for 10 min, followed by a base line measurement using an xCELLigence RTCA DP analyzer (Acea Biosciences, USA). Starved cells were detached with Accutase (Sigma Aldrich, Germany) and resuspended in DMEM (Sigma Aldrich, Germany). In a volume of 100 µl, 80,000 cells were added to the upper chamber. Migration was continuously monitored for the indicated time periods by measuring the electric impedance.

Cell growth was analyzed using electronically-integrated chamber slide plates (Acea Biosciences, USA) using an xCELLigence RTCA DP analyzer (Acea Biosciences, USA) according to the manufacturer.

## Enzyme-linked immunosorbent assay (ELISA)

Microplates (Sarstedt, Germany) were coated overnight at 4 °C with proteins diluted in PBS (AppliChem, Germany). Wells were washed twice using PBS (AppliChem, Germany) and blocked for 1 h at room temperature with blocking buffer (5% of bovine serum albumin (AppliChem, Germany) in PBS (AppliChem, Germany)). Wells were washed twice using PBS (AppliChem, Germany) and coated overnight at 4 °C with 1 µg/ml of primary antibody (diluted in blocking buffer). Wells were washed twice with PBS (AppliChem, Germany) and coated for 1 h at room temperature with horse horseradish peroxidase-coupled secondary antibody (Dianova, Germany) diluted in blocking buffer. Wells were washed twice using PBS and incubated for 20 min at room temperature with tetramethylbenzidine (Biolegend, USA). The reaction was stopped using 2 N of H_2_SO_4_ (Sigma Aldrich, Germany). The optical density was measured at 450 nm was measured using an iMark microplate absorbance reader (BioRad, Germany). Antibodies are listed in Table 5.

## Animal models

**Xenografting.** LN308*^EGFP-2A-FLuc^* cells were harvested, washed using PBS (AppliChem, Germany), counted and adjusted to 7.5 × 10^4^ in 5 µl of PBS (AppliChem, Germany). Mice were anesthetized by intraperitoneal injection of 0.5 mg/kg body weight of medetomidin (Pfizer, Germany), 5.2 mg/kg body weight of midazolam (Ratiopharm, Germany) and 0.052 mg/kg body weight of fentanyl (Janssen, Germany). Mice were analgized by intraperitoneal injection of 4 mg/kg body weight of carprofen (Pfizer, Germany) 15 min before surgery. Mice were fixed in a stereotactic head frame (Kopf Instruments, USA). A 1 cm to 1.5 cm longitudinal incision was made and a hole was drilled through the skull 1 mm lateral and 2 mm posterior from the *bregma*. The tumor cells were injected over 2 min at a depth of 2.5 mm using a 22 gauge syringe (Hamilton, Switzerland). For monitoring cell invasion into the corpus callosum, a hole was drilled 1 mm lateral and 1 mm posterior from the *bregma* and cells were injected at a depth of 1 mm. After injection, the syringe was left in place for additional 2 min and then slowly retracted. The incision was closed with stitches (Braun, Germany) and tissue glue (UHU, Germany). Mice were recovered from anesthesia by intraperitoneal injection of 2.6 mg/kg body weight of atipamezol (Pfizer, Germany), 0.5 mg/kg body weight of flumazenil (Roche, Germany) and 1.2 mg/kg body weight of naloxon (Inresa, Germany).

**Chemotherapy**. APY-29 (Tocris Bioscience, USA) was administered intraperitoneal (8 µM in 100 µl of PBS (AppliChem, Germany)) and intracranial (8 µM in 5 µl of PBS (AppliChem, Germany)) to determine the toxicity over time. Mice were investigated daily and weighted twice a week. Over 4 months, mice did not develop atypical behavior or obvious physical health deficits. For the treatment with APY-29 (8 µM in 5 ml PBS (AppliChem, Germany)), mice were injected intracranial seven days after tumor cell inoculation (each cohort n = 13). Therefore, mice were anesthetized and analgized as described before and the original longitudinal incision was re-opened. Mice were fixed in the stereotactic head frame (Kopf Instruments, USA), the same hole was used to inject 8 µM of APY-29 (Tocris Bioscience, USA) in 5 µl of PBS (AppliChem, Germany) at the same place and same depth. The incision was closed and mice were recovered from anesthesia as described before. For treatment with 80 mg/ kg body weight of temozolomide (Sigma Aldrich, Germany), tumor bearing mice were injected intraperitoneally starting at day 7 after tumor cell inoculation. Injections were repeated at day 10, 14 and 17 post tumor cell injection. Mice developed neurological symptoms or weight loss of >20% were euthanized using 100 mg/kg body weight of ketamine (Zoetis, Germany) and 16 mg/kg body weight of xylazine (Bayer, Germany) and perfused using 20 ml of PBS (AppliChem, Germany) and 20 ml of 4 % formaldehyde (Roth, Germany).

## Molecular modelling

**Bioinformatics.** The HHpred web server ^6^ and the AMAS method of multiple sequence alignment analysis^7^ was used to predict the conserved region in the Δ20 domain of Nogo-A.

***Ab initio* modeling.** The Robetta^8^ and PHYRE2^9^ webservers were used. The best three models of the protein domains in terms of DOPE score^10^ and stereochemical quality^11^ were selected for molecular simulations. We used PROCHECK^11^ to select the best models which had over 90% of the backbone dihedral angles within the core region of the Ramachandran plot. These three models were used as educated guesses for the subsequent molecular simulations and by no means represented a structural prediction.

**Molecular simulations.** Replica-exchange Monte Carlo algorithm (REMC) simulations^12^ using the PROtein Folding and Aggregation Simulator (PROFASI) code^12^ were carried out for the three models. PROFASI uses an all-atom model for the protein including hydrogen atoms and implicit water solvent. The model assumes fixed bond lengths, bond angles and peptide torsion angles (180°), so that each amino acid has the Ramachandran dihedral angles *φ* and *ψ* as well as a number of side-chain torsion angles as its degrees of freedom. The interaction potential assumes the presence of a solvent environment for the proteins although solvent molecules are not explicitly represented in the simulations. To explore the conformation space, we used a Markov Chain procedure. At any step, a conformation update involving a change in one or more backbone and side-chain torsion angles is proposed and is either accepted or rejected using a Metropolis criterion^12^. Biased Gaussian Steps^13^ producing smooth local deformations of the chains was used at the lower temperatures to improve the sampling of compact structures. The interaction potential is composed of four terms: $E=E_{loc}+E_{ev}+E_{hb}+E_{sc}$. The term $E_{loc}$ is a local potential that accounts for interactions between atoms separated by a few covalent bonds, such as the electrostatic interaction between adjacent peptide units along the chain. The other three terms are nonlocal in sequence. The excluded volume term $E_{ev}$ is a $1/r^{12}$repulsion between pairs of atoms. $E_{hb}$ represents two kinds of hydrogen bonds: backbone-backbone bonds, and bonds between charged side chains and the backbone. The last term $E_{sc}$ represents simple pairwise additive approximations for hydrophobic attraction between nonpolar side chains and electrostatic interactions among charged side-chains such as those forming salt-bridges. In each case, the system was enclosed in a periodic cubic box of size (300 Å)^3^. We used a replica exchange procedure^14^ to enhance the sampling in our simulations. Simultaneous Monte Carlo simulations were performed at eight temperatures: 293.0 K, 299.7 K, 306.5 K, 313.5 K, 320.6 K, 327.9 K, 335.4 K and 343.0 K. Periodically, conformations of replicas at consecutive temperatures were swapped with a probability

$P_{swap}^{ij}=\exp\left( \left( \frac{1}{k_{B}T_{i}}-\frac{1}{k_{B}T_{j}} \right)\left( E_{i}-E_{j} \right) \right)$,

where *T_k_* is the temperature and *E_k_* is the energy of replica *k*, and *k_B_* is the Boltzmann constant. The exchange procedure improves sampling at the lower temperatures by stochastically seeding them with independent states obtained at high temperatures, and the above exchange probability ensures that the equilibrium is maintained at each temperature even for the replica exchange step. The length of the simulations was chosen to allow our replicas to reach both ends of the temperature range repeatedly, so that our histograms received data from uncorrelated sample conformations from different parts of the energy landscape. We calculated the cumulative averages^15^ of a global property, the radius of gyration (R_g_), and a local property, the hydrogen bonds (HBs), over the sampled trajectory. The cumulative average was defined by $\sum_{i=1}^{N} {X^{i}}/N$, where *N* was the total number of frames sampled, *i* was the frame number and $X^{i}$ was the property of interest (the R_g_ or the HBs) calculated for each frame. The cumulative averages appeared to converge rather than systematically drift. We performed 2×10^7^ cycles of simulations for each system and the first 10% of each run was discarded for thermalization. Production trajectories obtained at 293.0 K were extracted, which yielded approximately 7000 frames per protein for analysis. As any modeling study, this work has limitations. First, without explicit water molecules, some potentially important questions cannot be addressed, such as the effect of ionic strength. Here, however, we stress that this computational approach has been successfully applied to a variety of intrinsically disordered proteins^16, 17, 18^. Thirdly, our REMC simulations cannot provide hints regarding time scales, which are the disadvantages compared with RE molecular dynamics (MD) techniques^19^. However, our REMC technique has the advantage in sampling efficiency, which allows studying processes that, because of the long time scales involved, would be currently infeasible with REMD simulations.

**Data analysis.** The following properties were calculated with the production trajectory obtained for each model: (i) The average R_g_ was calculated using g_gyrate in Gromacs 4.5 ^20^. (ii) The secondary structure in each frame, as well as the average SS content in the trajectory, were calculated using the DSSP software^21^. (iii) The hydrogen bonds were calculated by g_hbonds in Gromacs 4.5 with of cutoff of 3.5 A on the donor-acceptor distance and 30 degrees for the angle. (iv) The clustering procedure was performed using the Gromos cluster algorithm^22^ with a 2-Å RMSD cutoff on of protein backbone. The g_cluster module of Gromacs v4.5 ^20^ were used.

# Supplementary Tables

## Supplementary Table 1

Mass spectrometry of secreted proteins from glioma cells. Related to Figure 2.

Supplementary_Table 1.xlsx

| CELL LINE | SOUCRE |
| --- | --- |
| CHO | C Rösli (DKFZ, Germany) |
| HEK293 | ATCC, USA |
| LN18 | ATCC, USA |
| LNT229 | W Wick (DKFZ, Germany) |
| LN308 | ME Hegi (Lausanne University, Switzerland) |
| LN443 | ME Hegi (Lausanne University, Switzerland) |
| NCH82 | C Herold-Mende (Heidelberg University, Germany) |
| NCH89 | C Herold-Mende (Heidelberg University, Germany) |
| NCH125 | C Herold-Mende (Heidelberg University, Germany) |
| NCH210 | C Herold-Mende (Heidelberg University, Germany) |
| NCH270 | C Herold-Mende (Heidelberg University, Germany) |
| NCH342 | C Herold-Mende (Heidelberg University, Germany) |
| NCH343 | C Herold-Mende (Heidelberg University, Germany) |
| NCH351 | C Herold-Mende (Heidelberg University, Germany) |
| NCH354 | C Herold-Mende (Heidelberg University, Germany) |
| NCH375 | C Herold-Mende (Heidelberg University, Germany) |
| NCH378 | C Herold-Mende (Heidelberg University, Germany) |
| NCH390 | C Herold-Mende (Heidelberg University, Germany) |
| NCH417 | C Herold-Mende (Heidelberg University, Germany) |
| NCH465 | C Herold-Mende (Heidelberg University, Germany) |
| NCH601 | C Herold-Mende (Heidelberg University, Germany) |
| NIH-3T3 | ATCC, USA |
| T98G | ATCC, USA |

## Supplementary Table 2

Cell lines.

## Supplementary Table 3

Short hairpin RNA

| NAME | TARGET | TARGET SEQUENCE 5’-3’ | Source |
| --- | --- | --- | --- |
| sh*ENTPD5* | *ENTPD5* | GATCCGACGAAGGCATATTAG | 23 |
| sh*EXOSC10* | *EXOSC10* | CGTGGACTCAAACAAGCAATA | 24 |
| sh*GNA13* | *GNA13* | GACACTTGTTGCCTAACTTTA | 25 |
| sh*ERN1* | *ERN1* | GAGAAGATGATTGCGATGGAT | 26 |
| sh*PTEN* | *PTEN* | CCACAGCTAGAACTTATCAAA | 27 |
| sh*S1PR2* | *S1PR2* | CCTCTCTACGCCAAGCATTAT | 28 |
| shS1pr2 | *S1pr2* | ACCAAGGAGACGCTGGACATG | 29 |
| sh*CTR* | *control* | CAACAAGATGAAGAGCACCAA | 29 |
| sh*SPARC* | *SPARC* | AACAAGACCTTCGACTCTTCC | 30 |

## Supplementary Table 4

Primers

| NAME | SEQUENCE 5’-3’ |
| --- | --- |
| F-*EEF2* | CTGGAGATCTGCCTGAAGGA |
| R-*EEF2* | GAGACGACCGGGTCAGATT |
| F-*PGK1* | CAGCTGCTGGGTCTGTCAT |
| R-*PGK1* | GCTGGCTCGGCTTTAACC |
| F-*PPIA* | ATGCTGGACCCAACACAAAT |
| R-*PPIA* | TCTTTCACTTTGCCAAACACC |
| F-*SPARC* | TTCCCTGTACACTGGCAGTTC |
| R-*SPARC* | AATGCTCCATGGGGATGA |
| F-*ERN1* | GCCACCCTGCAAGAGTATGT |
| R-*ERN1* | ATGTTGAGGGAGTGGAGGTG |
| F-*EXOSC10* | CTGGCTGATTTCATCCATCA |
| R-*EXOSC10* | TTCATATTGATAAGGATGTGCAAAC |

## Supplementary Table 5

Antibodies

| NAME | CLONE | CATALOGUE NO | MANUFACTURER |
| --- | --- | --- | --- |
| α-ACTB | C-4 | sc-47778 | Santa Cruz Biotechnologies, Germany |
| α-AKT1 | C67E7 | 4691S | Cell Signaling Technology, USA |
| α-AKT1^S473^ | D9E | 4060S | Cell Signaling Technology, USA |
| α-CANX | Ab1 | HPA009433 | Sigma Aldrich, Germany |
| α-DsRed | L-18 | sc-33353 | Santa Cruz Biotechnologies, Germany |
| α-EF2 | C-14 | sc-13004 | Santa Cruz Biotechnologies, Germany |
| α-EGFP | FL | sc-8334 | Santa Cruz Biotechnologies, Germany |
| α-ENTPD5 |  | MAB5297 | R&D Systems, USA |
| α-GFAP | Z 0334 | Z033429-2 | Dako, Germany |
| α-Gα_13_ |  | 21005 | NewEast Biosciences, USA |
| α-Gα_13_^GTP^ |  | 26902 | NewEast Biosciences, USA |
| α-His | 13/45/31 | DIA-900 | Dianova, Germany |
| α-IRE1α | H-190 | Sc-20790 | Santa Cruz Biotechnologies, Germany |
| α-IRE1α^S724^ |  | PA1-16927 | Thermo Scientific, Germany |
| α-MLC-2 | D18E2 | 8505 | Cell Signaling Technology, USA |
| α-MLC-2^S19^ |  | 3671 | Cell Signaling Technology, USA |
| α-Myc | 9E10 | sc-40 | Santa Cruz Biotechnologies, Germany |
| α-Nestin | 10C2 | MAB5326 | Merck-Millipore, Germany |
| α-Nogo-A |  |  | M Schwab, Zürich University, Switzerland |
| α Nogo-A-Δ20 | 11c7 |  | M Schwab, Zürich University, Switzerland |
| α-PTEN | D4.3 | 9188 | Cell Signaling Technology, USA |
| α-PTEN |  | 9352S | Cell Signaling Technology, USA |
| α-RAC1 |  | ARH03 | Cytoskeleton, Inc., USA |
| α-RHOA |  | ARH04 | Cytoskeleton, Inc., USA |
| α-RHOA^GTP^ |  | 26904 | NewEast Biosciences, USA |
| α-ROCK | C8F7 | 4035S | Cell Signaling Technology, USA |
| α-S1PR2 | F3 | sc-365583 | Santa Cruz Biotechnologies, Germany |
| α-SPARC |  | AF941 | R&D Systems, USA |
| α-SPARC |  | 5420S | Cell Signaling Technology, USA |
| α-SPARC | A3A8 | sc-73472 | Santa Cruz Biotechnologies, Germany |

## Supplementary Table 6

Plasmids

| NAME | DESCRIPTION |
| --- | --- |
| pET28-Δ20 | Nogo-A (nt 1630– 2175) |
| pET28-Δ20N-RFP | Nogo-A (nt 1630-1797) fused to the N-terminus of RFP |
| pET28-Δ20C-RFP | Nogo-A (nt 1798-2175) fused to the C-terminus of RFP |
| pET28-Δ20min-RFP | Nogo-A (nt 1900-2055) fused to the C-terminus of RFP |
| pET28-ΔSCR | Scrambled version of Nogo-A (nt 1630– 2175) |
| pET32-SPARC | SPARC CDS (nt 52-909) |
| pET32-SPARC(del_Kazal) | SPARC CDS (nt 52-276 fused to nt 460-909) |
| pLKO.1-sh*ENTPD5* | shRNA against *ENTDP5* |
| pLKO.1-sh*ERN1* | shRNA against *ERN1* encoding IRE1α |
| pLKO.1-sh*EXOSC10* | shRNA against *EXOSC10* |
| pLKO.1-sh*GNA13* | shRNA against *GNA13* encoding Gα_13_ |
| pLKO.1-sh*PTEN* | shRNA against *PTEN* |
| pLKO.1-sh*S1PR2* | shRNA against *S1PR2* |
| pLKO.1-sh*S1pr2* | shRNA against *S1pr2* |
| pLKO.1-sh*CTR* | Control shRNA |
| pLKO.1-sh*CTR*-EGFP-FLuc | Control shRNA; CDS fusion of EGFP and FLuc with porcine teschovirus-1 2A |
| pLKO.1-sh*SPARC* | shRNA against *SPARC* |
| pLKO.1-sh*SPARC*-EGFP-FLuc | Control shRNA; CDS fusion of EGFP and FLuc with porcine teschovirus-1 2A |
| pLVX-ECL-2-EGFP | ECL2 of S1PR2 fused to the C-terminus of EGFP |
| pLVX-ECL-3-EGFP | ECL3 of S1PR2 fused to the C-terminus of EGFP |
| pLVX-ECL-scr-EGFP | Scrambled version of ECL3 fused to the C-terminus of EGFP |
| pLVX-EGFP | EGFP |
| pLVX-ENTPD5^E127A^-myc | ENTDP5 with point mutation E127A and N-terminal myc-tag |
| pLVX-ENTPD5-myc | ENTDP5 with N-terminal myc-tag |
| pLVX-IRE1^S724A^-myc | IRE1α with point mutation S724A and N-terminal myc-tag |
| pLVX-IRE1^K907A^-myc | IRE1α with point mutation K907A and N-terminal myc-tag |
| pLVX-Nogo-A-His | Nogo-A with N- and C-terminal His-tag |
| pLVX-Nogo-B-His | Nogo-B with N- and C-terminal His-tag |
| pLVX-PTEN-myc | PTEN with N-terminal myc-tag |
| pLVX-RHOA^G14V^-myc | RHOA with point mutation G14V and N-terminal myc-tag |
| pLVX-S1PR2-myc | S1PR2 with N-terminal myc-tag |
| pLVX-SPARC-EGFP | SPARC fused to EGFP |
| pLVX-SPARC(del_AC)-EGFP | SPARC CDS (nt 1-52 fused to nt 208-912) fused to EGFP |
| pLVX-SPARC(del_EC)-EGFP | SPARC CDS (nt 1-459 fused to 909-912) fused to EGFP |
| pLVX-SPARC(del_EGF)-EGFP | SPARC CDS (nt 1-207 fused to nt 277-912) fused to EGFP |
| pLVX-SPARC(del_Kazal)-EGFP | SPARC CDS (nt 1-276 fused to nt 460-912) fused to EGFP |
| pLVX-SP-EGFP | SPARC-derived signal peptide fused to EGFP |

## Supplementary Table 7

Compounds

| NAME | MANUFACTURER |
| --- | --- |
| APY-29 | Tocris Bioscience, USA |
| Blebbistatin | Cayman Chemicals, USA |
| CYM-5520 | Sigma Aldrich, Germany |
| Dimethyl sulfoxide | Genaxxon, Germany |
| Jasplakinolide | Enzo Lifescience, USA |
| JTE-013 | Sigma Aldrich, Germany |
| Latrunculin A | Enzo Lifescience, USA |
| MK-2206 | Cayman Chemicals, USA |
| Pertussis toxin | Cayman Chemicals, USA |
| Y-27632 | Enzo Lifescience, USA |

# Supplementary References

1. Schwarz MK, Scherbarth A, Sprengel R, Engelhardt J, Theer P, Giese G. Fluorescent-protein stabilization and high-resolution imaging of cleared, intact mouse brains. *PLoS One* **10**, e0124650 (2015).

2. Breckwoldt MO*, et al.* Correlated magnetic resonance imaging and ultramicroscopy (MR-UM) is a tool kit to assess the dynamics of glioma angiogenesis. *Elife* **5**, e11712 (2016).

3. Pfaffl MW. A new mathematical model for relative quantification in real-time RT-PCR. *Nucleic Acids Res* **29**, e45 (2001).

4. Manza LL, Stamer SL, Ham AJ, Codreanu SG, Liebler DC. Sample preparation and digestion for proteomic analyses using spin filters. *Proteomics* **5**, 1742-1745 (2005).

5. Burkhart JM, Schumbrutzki C, Wortelkamp S, Sickmann A, Zahedi RP. Systematic and quantitative comparison of digest efficiency and specificity reveals the impact of trypsin quality on MS-based proteomics. *J Proteomics* **75**, 1454-1462 (2012).

6. Soding J, Biegert A, Lupas AN. The HHpred interactive server for protein homology detection and structure prediction. *Nucleic Acids Res* **33**, W244-248 (2005).

7. Livingstone CD, Barton GJ. Protein sequence alignments: a strategy for the hierarchical analysis of residue conservation. *Comput Appl Biosci* **9**, 745-756 (1993).

8. Kim DE, Chivian D, Baker D. Protein structure prediction and analysis using the Robetta server. *Nucleic Acids Res* **32**, W526-531 (2004).

9. Kelley LA, Sternberg MJ. Protein structure prediction on the Web: a case study using the Phyre server. *Nat Protoc* **4**, 363-371 (2009).

10. Eswar N*, et al.* Comparative protein structure modeling using Modeller. *Curr Protoc Bioinformatics* **Chapter 5**, Unit 5 6 (2006).

11. Laskowski RA, MacArthur MW, Moss DS, Thornton JM. PROCHECK: a program to check the stereochemical quality of protein structures. *Journal of Applied Crystallography* **26**, 283-291 (1993).

12. Lyubartsev AP, Martsinovski AA, Shevkunov SV, Vorontsov‐Velyaminov PN. New approach to Monte Carlo calculation of the free energy: Method of expanded ensembles. *The Journal of Chemical Physics* **96**, 1776-1783 (1992).

13. Favrin G, Irbäck A, Sjunnesson F. Monte Carlo update for chain molecules: Biased Gaussian steps in torsional space. *The Journal of Chemical Physics* **114**, 8154-8158 (2001).

14. Hukushima K, Nemoto K. Exchange Monte Carlo Method and Application to Spin Glass Simulations. *Journal of the Physical Society of Japan* **65**, 1604-1608 (1996).

15. Johansen AM. Monte Carlo Methods A2 - Peterson, Penelope. In: *International Encyclopedia of Education (Third Edition)* (ed^(eds Baker E, McGaw B). Elsevier (2010).

16. Cong X*, et al.* Role of Prion Disease-Linked Mutations in the Intrinsically Disordered N-Terminal Domain of the Prion Protein. *J Chem Theory Comput* **9**, 5158-5167 (2013).

17. Jonsson SA, Mohanty S, Irback A. Distinct phases of free alpha-synuclein--a Monte Carlo study. *Proteins* **80**, 2169-2177 (2012).

18. Li DW, Mohanty S, Irback A, Huo S. Formation and growth of oligomers: a Monte Carlo study of an amyloid tau fragment. *PLoS Comput Biol* **4**, e1000238 (2008).

19. Baillod P, Garrec J, Colombo MC, Tavernelli I, Rothlisberger U. Enhanced sampling molecular dynamics identifies PrP(Sc) structures harboring a C-terminal beta-core. *Biochemistry* **51**, 9891-9899 (2012).

20. Pronk S*, et al.* GROMACS 4.5: a high-throughput and highly parallel open source molecular simulation toolkit. *Bioinformatics* **29**, 845-854 (2013).

21. Kabsch W, Sander C. Dictionary of protein secondary structure: pattern recognition of hydrogen-bonded and geometrical features. *Biopolymers* **22**, 2577-2637 (1983).

22. Daura X, Gademann K, Jaun B, Seebach D, van Gunsteren WF, Mark AE. Peptidfaltung: Wenn die Simulation das Experiment erreicht. *Angewandte Chemie* **111**, 249-253 (1999).

23. Fang M*, et al.* The ER UDPase ENTPD5 promotes protein N-glycosylation, the Warburg effect, and proliferation in the PTEN pathway. *Cell* **143**, 711-724 (2010).

24. Li Y, Masaki T, Yamane D, McGivern DR, Lemon SM. Competing and noncompeting activities of miR-122 and the 5' exonuclease Xrn1 in regulation of hepatitis C virus replication. *Proc Natl Acad Sci U S A* **110**, 1881-1886 (2013).

25. Buch TR*, et al.* G13-dependent activation of MAPK by thyrotropin. *J Biol Chem* **283**, 20330-20341 (2008).

26. Liu J*, et al.* Virus-induced unfolded protein response attenuates antiviral defenses via phosphorylation-dependent degradation of the type I interferon receptor. *Cell Host Microbe* **5**, 72-83 (2009).

27. Kluth M*, et al.* Genomic deletion of MAP3K7 at 6q12-22 is associated with early PSA recurrence in prostate cancer and absence of TMPRSS2:ERG fusions. *Mod Pathol* **26**, 975-983 (2013).

28. Liu R*, et al.* Conjugated bile acids promote cholangiocarcinoma cell invasive growth through activation of sphingosine 1-phosphate receptor 2. *Hepatology* **60**, 908-918 (2014).

29. Kempf A*, et al.* The sphingolipid receptor S1PR2 is a receptor for Nogo-a repressing synaptic plasticity. *PLoS Biol* **12**, e1001763 (2014).

30. Chang W, Wei K, Jacobs SS, Upadhyay D, Weill D, Rosen GD. SPARC suppresses apoptosis of idiopathic pulmonary fibrosis fibroblasts through constitutive activation of beta-catenin. *J Biol Chem* **285**, 8196-8206 (2010).
